# Cancer gene identification from RNA variant allelic frequencies using *RVdriver*

**DOI:** 10.1186/s13059-025-03557-y

**Published:** 2025-06-13

**Authors:** James R. M. Black, Thomas P. Jones, Carlos Martínez-Ruiz, Maria Litovchenko, Clare Puttick, Charles Swanton, Nicholas McGranahan

**Affiliations:** 1 Cancer Research UK Lung Cancer Centre of Excellence, University College London Cancer Institute, London, UK; 2 Cancer Genome Evolution Research Group, University College London Cancer Institute, London, UK; 3 Cancer Evolution and Genome Instability Laboratory, the Francis Crick Institute, London, UK

## Abstract

Existing approaches to identifying cancer genes rely overwhelmingly on DNA sequencing data. Here, we introduce *RVdriver*, a computational tool that leverages paired bulk genomic and transcriptomic data to classify RNA variant allele frequencies (VAFs) of non-synonymous mutations relative to a synonymous mutation background. We analyze 7882 paired exomes and transcriptomes from 31 cancer types and identify novel, as well as known, cancer genes, complementing other DNA-based approaches. Furthermore, RNA VAFs of individual mutations are able to distinguish “driver” from “passenger” mutations within established cancer genes. This approach highlights the value of multi-omic approaches for cancer gene discovery.

**Supplementary Information:**

The online version contains supplementary material available at 10.1186/s13059-025-03557-y.

## Background

Tumors evolve in a Darwinian fashion, whereby cancer cells acquire heritable variation that acts as the substrate for selection in the context of its environment. Cancer cell phenotypes are profoundly different from those of cells found in non-cancer tissues, and different cancers often share common phenotypes, or hallmarks [1]. This divergence from normal cell phenotype occurs as selection acts upon sequentially acquired, heritable variation [2]. The best studied form of such variation is genetic point mutations.

A central goal of cancer research has been to identify which somatic alterations are the key drivers of cancer initiation, development, and metastasis. As such, multiple algorithms and computational tools have been developed to identify patterns of excessive mutagenesis and thus “cancer genes” which are causally implicated in cancer. These include approaches to define genes that are more frequently mutated than would be expected by chance [3, 4]; harbor more nonsynonymous mutations relative to an expected background derived by considering the observed mutational processes in a given set of cancer genomes [5–8]; mutations that tend to cluster within genes [9–12], recur in loci encoding specific regions of the 3D protein confirmation [13], or occur in domains which might be expected to be functionally relevant to the encoded protein [14, 15]. Additionally, there have been other efforts to study genes according to whether their constituent mutations are more recurrently clonal, or are preserved or gained more frequently in the context of somatic copy-number alterations, than would be expected by chance [16–18].

Such efforts are relatively advanced, and a decade of gradual improvement has facilitated the development of machine-learning approaches that leverage genomic output and deliver interpretable assessments of cancer genes and driver mutations [19].

However, it is notable that the vast majority of tools and approaches to identify cancer genes and driver mutations focus almost exclusively on genomic data. Many genomic studies also collect transcriptomic data [20]. Usage of this data to uncover novel cancer genes is relatively nascent [21]. Some studies utilize paired or unpaired gene expression data, either to alleviate the burden of testing for repeated measures by pruning lists of putative cancer genes to exclude those that are never expressed [5], or by leveraging the observation that cancer genes tend to be more uniformly expressed than non-cancer genes [22, 23]. However, approaches that incorporate transcriptomic information as a window into tumor phenotype through which cancer genes can be identified are lacking.

We hypothesized that RNA information may enhance and improve our power to identify the drivers of cancer development and shed light on their key features. Using 7882 paired whole exome-RNA-seq samples taken from tumors across 31 cancer types from the Cancer Genome Atlas (TCGA), we set out to understand the capacity of transcriptomic data to contribute to cancer gene identification. We present “RNA VAF driver” (*RVdriver*), a cohort-level bioinformatic algorithm to identify cancer genes, which exploits paired WES/WGS and RNA-seq data. Leveraging *RVdriver*, we revealed that the variant allele frequencies of somatic mutations, when observed in RNA, were capable of distinguishing cancer genes from non-cancer genes across multiple cancer types with comparable sensitivity and specificity to a suite of best-in-class tools which leverage DNA information. Furthermore, within individual mutations, signals from RNA VAF provided meaningful information to enhance existing “driver” mutation discovery tools.

## Results

### Mutation expression in solid tumors

We collated a pan-cancer cohort of tumors with paired whole-exome and RNA sequencing from the Cancer Genome Atlas (TCGA). Hypermutator tumors, samples which had a significant degree of RNA degradation, samples previously flagged based on pathology, and samples without a single expressed mutation were removed [24]. In total, the cohort comprised 7957 samples across 31 cancer types (Additional file 1: Figure S1; Additional file 2: Table S1). Mutations from these samples were derived from the publically available “MC3” mutation table [25]. In total, these tumors collectively harbored 969,177 somatic mutations, which were further filtered to remove single-nucleotide variants within the ENCODE blacklist [26], leaving 911,592 mutations across 7882 samples. Existing usage of transcriptomic data in tools to identify cancer genes has typically focussed on information relating to gene expression amplitude, or inter-sample heterogeneity. Instead, we processed samples to obtain information relating to the expression of individual mutations.

In the first instance, this facilitated analysis of patterns of mutation expression in solid tumors. Overall, 39% of somatic point mutations were expressed (defined as > 1 non-duplicated read aligning to the mutated allele at the mutated position) across all samples, while at 41% of mutated positions, there was no expression from either the wild-type or mutated allele (defined as < 2 non-duplicated reads aligning to the mutated or wild-type allele at the mutated position). The remaining 20% corresponds to mutated positions at which only the wild-type allele is expressed (Additional file 1: Figure S2A).

**Fig. 1:**
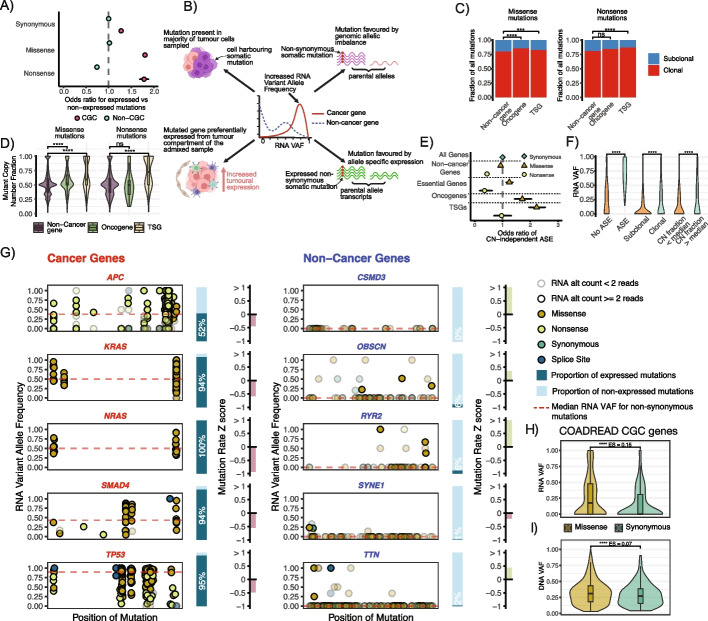
RNA VAF across cancer and non-cancer genes. **A** Odds ratios for the expression of mutations in genes found, or not found, within the COSMIC cancer gene census (CGC) list. Mutations were identified within the “MC3” mutation table, within samples containing paired DNA-RNA sequencing information. For a mutation to be classified as expressed, two or more molecules mapping to the mutated allele had to be identified within RNA. Odds ratios were estimated using the number of expressed and non-expressed mutations observed of a given type, relative to synonymous mutations in non-CGC genes using Fisher’s exact test; bars indicate 95% confidence intervals for the odds ratio estimate. **B** Mechanisms of elevated variant allele frequency observed in RNA.** C **Proportion of all mutations within the MC3 mutation table which were estimated to be present in 100% of tumor within the admixed bulk sample; split by mutation type, and by the function of the gene containing the mutation. **D** Ratio of the estimated mutated allele copy-number relative to the total copy-number at that position; split by mutation type, and by the function of the gene containing the mutation. **E** Odds ratio of missense and nonsense mutations exhibiting allele-specific expression, beyond that expected given the copy-number status of the mutation if there were 100% expression from the tumor compartment of the admixed sample. Odds ratio and 95% confidence intervals defined relative to synonymous mutations among all genes. **F** RNA VAFs of somatic mutations across conditions in **C**, **D**, and **E**. **** indicates *p* < 0.0001; Wilcoxon test. **G** RNA VAFs of somatic mutations within putative cancer genes in colorectal tumors, and RNA VAFs of somatic mutations within putative non-cancer genes in colorectal tumors. Barplot percentages indicate the proportion of nonsynonymous mutations that are expressed. *Z*-scores for gene mutation rates compared to all other genes are also shown. **H** RNA VAF of synonymous versus missense mutations within COSMIC cancer gene census genes; **** indicates *p* < 0.0001; Wilcoxon test. ES: effect size. **I** DNA VAF of synonymous versus missense mutations within COSMIC cancer gene census genes; **** indicates *p* < 0.0001; Wilcoxon test. ES: effect size

This relationship differed across synonymous, missense, and nonsense mutations (Figs. 1A, Additional file 1: S2B), as well as across genes within or outside of the COSMIC Cancer Gene Census (CGC) list of cancer genes [24, 27] (mutations within these genes might be more likely to be functionally impactful), and was broadly consistent across cancer types (Additional file 1: Figures S3, S4). In non-CGC genes, nonsense mutations were less likely to be expressed than synonymous or missense mutations, in keeping with the degradation of these mutated transcripts through nonsense-mediated decay (Fig. 1A). Synonymous or missense mutations in these genes were expressed to a similar extent. Of note, though, within CGC genes, synonymous mutations were less likely to be expressed than missense mutations or nonsense mutations within these genes (Fig. 1A). This might relate to concurrent copy-number loss of wild-type alleles in the context of deleterious mutations in tumor suppressor genes, where paradoxical overexpression of mutated alleles has previously been described [28]. Regardless of the mechanism, within these genes the likely degradation of truncated transcripts through nonsense-mediated decay does not prevent functionally impactful nonsynonymous mutations, independent of whether they encode a simple base change or a frameshift, from being more frequently expressed than non-impactful mutations. This analysis highlights that mutation expression data can encode information relating to functional relevance of genes.

### RNA VAF

Mutation expression can be considered in conjunction with expression of the wild-type allele. This has utility because in most biological settings, expression of one parental allele positively correlates with expression of the other parental allele [29]. It follows that if functionally important information is encoded in deviation from this pattern, mutated allelic expression changes might be best observed in the context of wild-type allelic expression.

To that end, we leveraged *RVdriver* to calculate RNA variant allele frequencies (RNA VAF) for each mutation, by dividing the number of non-duplicated reads mapping to the variant allele by the total number of non-duplicated reads mapping to either parental allele at that position.

The RNA VAF of each somatic mutation might plausibly be influenced by a variety of factors, some of which relate to the specific mutation, and others that are gene-level or global (Fig. 1B). Mutation-level factors that would influence RNA VAF include the proportion of sampled tumor cells containing the somatic mutation (cancer cell fraction, CCF); the degree of allelic imbalance in genomic copy number state between mutated and non-mutated alleles; and allele-specific expression (where mutated and non-mutated alleles are differentially expressed relative to their genomic copy-number). Gene or sample-level factors include the fraction of the admixed bulk sample derived from the tumor (purity); the degree to which the mutated gene is expressed; and global differences in RNA content between the tumor and non-tumor components of the admixed sample [22, 30, 31]. Thus, a multitude of information might be encoded within a simple, continuous metric, RNA VAF, which to-date has not been used to identify cancer genes.

### RNA VAF differentiates between mutations in cancer genes and non-cancer genes

We next evaluated the extent to which these mutation-level variables influencing RNA VAF differed between mutations in cancer genes and non-cancer genes.

First, we compared CCF, measuring the clonal status of mutations, between nonsynonymous mutations within oncogenes, tumor suppressor genes (both defined by Bailey et al. [24]), and non-cancer genes (genes not found within CGC list or Bailey list). This information has previously been shown to be informative when identifying cancer genes [17]. CCF was calculated using a previously published approach [16]. A large proportion of mutations were estimated to have a CCF of 1 (i.e., estimated to be present within 100% of tumor cells within the admixed bulk sample). Missense mutations within oncogenes and tumor suppressor genes typically exhibited higher CCF compared to those within non-cancer genes (Fig. 1C), while nonsense mutations had elevated CCF within tumor suppressor genes.

Second, we evaluated whether allelic imbalance tended to favor the mutated allele (i.e., mutated alleles were preferentially gained or preserved in copy number events relative to the wild-type allele) more in cancer genes than non-cancer genes. This has been demonstrated to be the case previously in oncogenes [32], and wild-type copies of tumor suppressor genes are lost canonically in carcinogenesis [33–35]. For each mutation, we calculated its copy-number, estimated using its variant allele frequency in DNA as well as purity and the total copy-number at that position (calculated using ASCAT [36]; for equation see “ Methods”). We then divided this by the total copy-number at the position to obtain a mutant copy-number fraction. Missense mutations within oncogenes, and missense and nonsense mutations within tumor suppressors were typically more likely to be favored in allelic imbalance, relative to mutations within non-cancer genes (Fig. 1D).

Third, we investigated the extent of copy-number independent allele-specific expression (ASE) in mutations within cancer genes relative to non-cancer genes. The observation that expression relative to genomic content is imbalanced between the tumor and non-tumor components of the admixed sample (made in multiple recent papers [22, 30, 31]) could plausibly confound a direct measurement of allelic expression relative to DNA allele frequencies. We therefore adopted a conservative approach to detect ASE, sensitive to differences between tumor purity and tumor transcript fraction. We considered ASE within non-CGC genes, cancer-cell essential genes, oncogenes, and tumor suppressor genes. When measured this way, as expected, ASE favoring the expression of the mutated allele was seen more commonly within missense than nonsense mutations. Missense mutations more frequently showed ASE in oncogenes and tumor suppressor genes than essential genes, while nonsense mutations showed ASE more frequently in tumor suppressor genes than essential genes (Fig. 1E). In this context, repression of the wild-type allele of a canonical tumor-suppressor gene represents a copy-number independent mechanism of allelic inactivation, and highlights that ASE can generate functional variation in tumors [22, 37].

Thus, measurements of the clonal status of the mutation (measured as CCF), allelic imbalance, and allele-specific expression all exhibit differences between cancer genes and non-cancer genes, and provide rationale for the use of RNA VAF to identify cancer genes. Indeed, clonal mutations, mutations in allelic imbalance, and mutations showing allele-specific expression exhibited elevated RNA VAF (Fig. 1F).

We therefore tested the ability of RNA VAF to differentiate between cancer genes and non-cancer genes. We initially considered five canonical cancer genes (*APC, KRAS, NRAS, SMAD4 *and *TP53*) and five non-cancer genes (*CSMD3, OBSCN, RYR2, SYNE1 *and* TTN*) in colonic and rectal tumors from TCGA. Clear differences were observed comparing the distributions of RNA VAFs between cancer genes and non-cancer genes, with non-cancer genes tending to harbor lower RNA VAFs than cancer genes (Fig. 1G). Importantly, there did not appear to be an obvious bias in the data such that genes with a high mutation rate might falsely exhibit patterns of positive selection.

We then assessed whether RNA VAF might encode differences between functional and non-functional mutations within putative cancer genes. To assess this signal, we considered mutations in CGC genes within colorectal cancer. We postulated that relative differences in RNA VAF between non-synonymous and synonymous mutations within cancer genes might represent a signal of selection captured by this metric. Indeed, there was a significant difference in RNA VAF between non-synonymous and synonymous within CGC genes (Fig. 1H, Wilcoxon *p* < 2.2e − 16). This difference was more pronounced than when considering DNA VAF (Wilcoxon effect size 0.16 compared to 0.08; Fig. 1I). This pattern was observed across the majority of cancer types tested, as well as pan cancer (Additional file 1: Figures S5A-C, S6, S7).

### RVdriver

To leverage RNA VAF information for the identification of cancer genes more systematically, we developed an approach, *RVdriver*, to test the observed RNA VAFs of mutations against an expected background (Methods) and implemented this across each of the cancer types within TCGA.

Briefly, within the cohort of tumors of each cancer type, we considered each gene that harboured, across all tumours of that cancer type, a sufficient number of nonsynonymous mutations to test against an expected background. This threshold was a minimum of 2 nonsynonymous mutations within a given gene at a minimum required depth of 8 non-duplicated reads.

A background set of mutations was generated for each comparison. We utilized a number of synonymous mutations that scaled with the number of available nonsynonymous mutations in the gene of interest, sampled without replacement from across all genes. In rare cases where insufficient synonymous mutations exist, this was supplemented by nonsynonymous mutations (Methods, Additional file 1: Figure S8).

The RNA VAFs of all nonsynonymous mutations within a given gene (regardless of RNA depth) were then tested against the background mutations. Sampling of consistent numbers of synonymous mutations across tumors was performed to minimize the potential confounding impact of inter-tumor differences in the distribution of RNA VAFs. Furthermore, the impact of lowly expressed genes was mitigated by selecting only synonymous mutations with RNA coverage > 7 non-duplicated reads (Methods).

We then scaled the observed RNA VAFs of the mutations of interest to account for nonsense-mediated decay (NMD). This process asymmetrically affects nonsense mutations [38] (Additional file 1: Figures S9A-C, “ Methods”). Further scaling was performed to account for systematic differences between the RNA VAFs of populations of nonsynonymous mutations and synonymous (background) mutations that were tested (Methods).

A linear model was then computed, comparing RNA VAFs for the set of nonsynonymous mutations within the gene of interest to those of the “background” set of synonymous mutations from across genes. This model was weighted by the RNA coverage at the mutated position to mitigate potential false positives arising from mutations in lowly expressed genes (Methods). This selection of synonymous mutations and statistical test was bootstrapped 25 times in order to limit the impact of sampling, given VAFs are variable across mutations. A *p*-value was obtained when considering each (i) missense mutations only within the gene; (ii) nonsense mutations only within the gene; (iii) splice site mutations only within the gene; and (iv) all nonsynonymous mutations within the gene. For cancer type specific analyses, the lowest *p*-value of i–iii was extracted; for pan-cancer analysis, iv was extracted. Genes with a q-value < 0.25 were considered to be putative cancer genes.

### Performance of RVdriver relative to genomic approaches

We applied *RVdriver* to our cohort of 7882 tumors. Across the 31 cancer types, it identified 235 significant genes, of which 186 were CGC genes (here, as elsewhere [7], used as a proxy for the true-positive rate), acting as putative cancer genes in a cancer type, compared to 49 non-CGC genes, highlighting its ability to identify meaningful biological patterns.

**Fig. 2:**
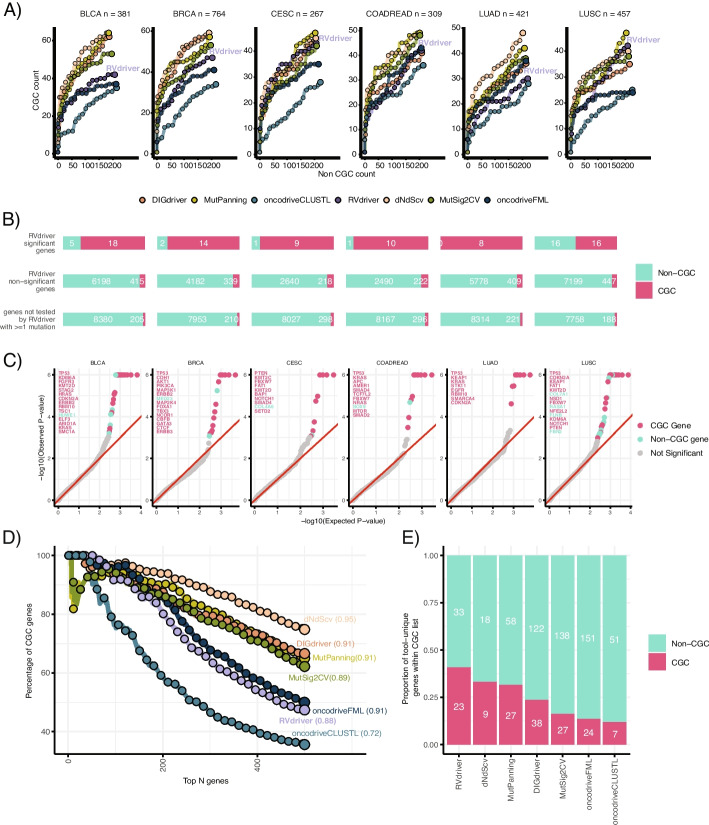
Performance of *RVdriver* identifying cancer genes.** A** Benchmarking of *RVdriver* in six cancer types (BLCA = bladder adenocarcinoma; BRCA = breast carcinoma; CESC = cervical squamous cell carcinoma; COADREAD = colorectal carcinoma; LUAD = lung adenocarcinoma; LUSC = lung squamous cell carcinoma) against six other established tools leveraging DNA information, *DIGdriver*, *dNdScv*, *MutPanning, MutSig2CV, oncodriveCLUSTL*, and *oncodriverFML*. Genes within the COSMIC cancer gene census list were treated as true positive results, and other genes as true negative results. The figure displays the number of CGC genes (*y*-axis), versus non-CGC genes (*x*-axis), identified within the top hits by the tools. Dots indicate the CGC to non-CGC gene ratio at every 15th gene ranked by increasing *p-value*. *n* represents the number of samples tested per cancer type. **B** The number of genes assigned as putative cancer genes by *RVdriver* **(**top); the number of genes tested by *RVdriver* that were not assigned as putative cancer genes (middle); the number of genes with ≥ 1 mutation that were not tested by *RVdriver* (bottom). Each panel is split into CGC and non-CGC genes. **C** QQ plots for *RVdriver*. Significant genes (*q* value < 0.25) are highlighted in red (CGC gene) and green (Non-CGC gene). Gene names are displayed in descending order of statistical significance until the 15th most significant gene. **D** Pan-cancer enrichment for CGC genes across the 7 tested tools. The CGC score for each tool, shown in brackets, is a ranking score for the proportion of CGC genes within the top 250 genes (see “ Methods”). **E** Stacked barplot showing putative gene-cancer-type combinations uniquely identified by each tool. The bars are ordered by the ratio of CGC:non-CGC genes. The number of genes identified uniquely by each method is shown within the bars

We next sought to understand whether our approach provided additional information in the context of cancer gene identification compared to methods leveraging DNA data alone. In order to do this, we computed output from six tools, *DIGdriver* [8], *dNdScv* [5], *MutPanning* [7], *MutSig2CV* [4], *oncodriveCLUSTL* [10], and *oncodriveFML* [15], which were run separately on the cohort of tumors for which paired DNA and RNAseq were available. These tools, respectively, consider local variability of somatic mutation rates, the difference between synonymous and nonsynonymous mutations at their putative sites, the nucleotide context of mutations, mutation rates of genes relative to a background, clustering of mutations around putative hotspots, and the predicted functional impact of mutations. We evaluated the relative performance of these tools by assessing the number of CGC genes compared to non-CGC genes discovered by each tool (using thresholds specified by each tool, see “ Methods”). Within our cohort, *RVdriver* exhibited comparable performance relative to other tools (Fig. 2A, Additional file 1: Figure S10). Of note, given a minimum requirement of two mutations per cancer type, it is possible that our approach would be predisposed to discover more CGC genes than a random background, given the propensity of these genes to be more frequently mutated. The number of genes not considered by *RVdriver* is displayed in Fig. 2B and Additional file 1: Figure S11. Additionally, the analysis performed in Fig. 2A was repeated using a unified geneset tested by all tools, again showing comparable performance by *RVdriver* (Additional file 1: Figure S12).

We next tested the expected and observed distribution of *p*-values derived from *RVdriver*. As evidenced by quantile–quantile (QQ) plots, the majority of genes followed the expected distribution of random *p*-values, aside from a minority of genes in which RNA VAF indicated a significant deviation from this background (Fig. 2C, Additional file 1: S13, S14).

We compared quantitatively the ability of *RVdriver* and the DNA-based tools to identify preferentially CGC genes. To do this, we leveraged a previously published approach that computes a “CGC score” [39] (Methods). This revealed that *RVdriver* could reliably identify cancer genes (Fig. 2D).

We studied the degree to which putative cancer gene-cancer type combinations identified by *RVdriver* overlapped with those identified by *DIGdriver* [8], *dNdScv* [5], *MutPanning* [7], *MutSig2CV* [4], *oncodriveCLUSTL* [10], and *oncodriveFML* [15]. The *RVdriver* unique gene-set exhibited the highest ratio of CGC genes compared to non-CGC genes relative to other tools (Fig. 2E). *RVdriver* uniquely identified 23 CGC gene-cancer type combinations, and 33 non-CGC gene-cancer type combinations. This suggests *RVdriver* can identify *bona fide* novel cancer genes. The degree of overlap between *RVdriver* and the DNA-based approaches is shown in Additional file 1: Figure S15A. Of note, there was significant enrichment for X chromosome gene-cancer type combinations in *RVdriver* unique drivers compared to the suite of DNA-based tools, 15/56 and 28/482, respectively (Fisher’s exact test, *p* = 2.6e − 06, Additional file 1: Figure S15B), with 6/13 of the unique genes identified being CGC genes.

### Putative cancer genes identified by RVdriver

**Fig. 3:**
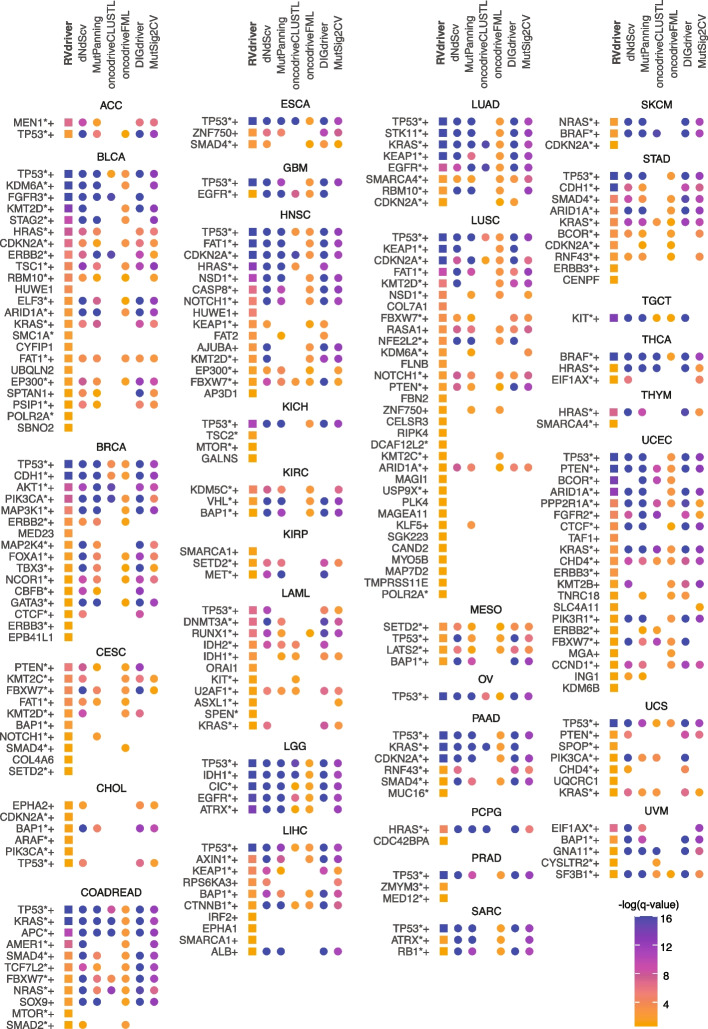
Cancer genes identified by *RVdriver* and DNA approaches. Using paired whole-exome and RNA sequencing from 7882 tumors across 31 cancer types, *RVdriver*, *dNdScv*, *MutPanning*, *DIGdriver*, *MutSig2CV*, *oncodriveCLUSTL*, and *oncodriverfml* were used to identify putative cancer genes. Point color indicates the negative log of the *q* value outputted by the tool. Only genes with a *q* value of less than 0.25 in *RVdriver* are plotted. + indicates gene identified as significant by Bailey et al. within that cancer type. * indicates gene contained within the CGC list

The output from *RVdriver* across the 31 cancer types in TCGA is displayed in Fig. 3. This analysis highlights specific cancer genes that were identified by the MC3 group within a near-identical (excluding approximately 1300 samples with DNA information available only) dataset [24], as well as CGC genes.

Many canonical cancer genes were identified using *RVdriver*. For example, *TP53* is highlighted as a cancer gene in 22 cancer types. However, there are other putative cancer genes identified by *RVdriver* that are not identified by the DNA tools. These include cancer genes that were not highlighted as such within that cancer type, such as *ERBB3* (breast); *SMC1A* and *POLR2A* (bladder); *BAP1* and *SETD2* (cervical); *MTOR* (colorectal); *USP9X* and *POLR2A* (lung squamous cell carcinoma); *MED12* and *ZMYM3* (prostate); *CDKN2A* (melanoma); *ERBB3* (stomach); and *ERBB3* (endometrial). Importantly, *RVdriver* also implicated several novel cancer genes that would be worthy of further investigation and analysis to determine their role in cancer development and progression. Certain such examples are overviewed in the “ Discussion.”

### Validation of RVdriver and putative cancer genes

**Fig. 4:**
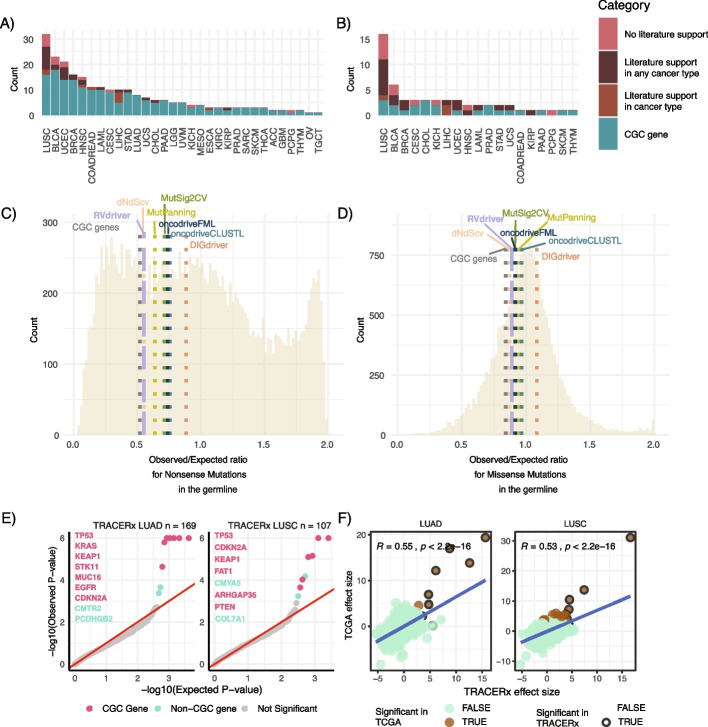
Validation of *RVdriver* and putative cancer genes. **A** Putative cancer genes identified by *RVdriver*. Colors within the bars reflect the level of support for the putative cancer gene-cancer type combination. CGC gene: gene identified within the Cancer Gene Census. Literature support was defined using CancerMine. The majority of *RVdriver* non-CGC putative cancer genes had literature support. **B** Putative cancer genes identified by *RVdriver* and not by any of *DIGdriver*, *dNdScv*, *MutPanning*, *MutSig2CV*, *oncodriveCLUSTL*, and *oncodriveFML*. Colors within the bars reflect the level of support for the *RVdriver* putative cancer gene-cancer type combination. CGC gene: gene identified within the Cancer Gene Census. Literature support was defined using CancerMine. The majority of non-CGC putative cancer genes identified by *RVdriver* alone (and not identified by DNA based approaches) had literature support. **C**, **D** Mean “essentiality” score for putative cancer genes identified uniquely by each tool. Essentiality scores were calculated as the Observed-Expected upper bound fraction for germline nonsense mutations (**C**) and missense mutations (**D**). Background histograms represent the scores across all genes. The average scores for CGC genes are also shown. **E** QQ plots for *RVdriver* in the TRACERx lung cancer cohort. Significant genes (*q* value < 0.25) are highlighted in red (CGC gene) and green (Non-CGC gene). Gene names are displayed in descending order of statistical significance. *n* represents the number of samples tested per cancer type. **F** Concordance between TCGA and TRACERx results for LUAD and LUSC tumors. The effect sizes output by *RVdriver* are compared across 5052 genes tested in both cohorts

We sought to validate the putative cancer genes identified by *RVdriver*. First, we leveraged CancerMine [40] to evaluate the degree of experimental and clinical literature support for significant hits from *RVdriver*, and in particular those not found within the CGC list. We stratified genes into four levels on the strength of literature support: (i) CGC genes; (ii) genes with literature support within the cancer type in which the association was observed; (iii) genes with literature support in any cancer type; (iv) genes without literature support. *RVdriver* hits were more likely to be CGC than non-CGC genes, but among non-CGC genes, there was some literature support for 35 out of 49 putative cancer genes (Fig. 4A). Indeed, when considering putative cancer genes identified uniquely by *RVdriver* (among the 7 tools tested), literature support was found for 22 out of 33 putative cancer genes.

Next, we leveraged the gnomAD database [41] to query the “essentiality” of *RVdriver* putative non-CGC cancer genes. Cancer genes, known to disrupt core cellular pathways, typically have higher “essentiality” scores for cell viability compared to other genes, reflective of negative selection in the germline (Additional file 1: Figure S16A). We investigated the essentiality scores, reflecting the tolerance of genes to missense and nonsense germline events and thereby their necessity for cell viability. Reassuringly, the putative novel cancer genes highlighted by RVdriver exhibited very low essentiality scores, suggesting independently and orthogonally that they are of high cellular importance, supporting a role in an aberrant tumor phenotype (Fig. 4C, D, Additional file 1: Figures S16B-C). Indeed, the mean score for nonsense mutations (0.552) and missense mutations (0.889) of *RVdriver* putative cancer genes was lower than 5 out of 6 other tools (Fig. 4C and D).

We would expect *bona fide* cancer genes to be recurrently identified across cohorts, reflecting consistent positive selection in cancer. As such, we interrogated the TRACERx 421 cohort [22], for which paired multi-region DNA and RNAseq was available for 276 tumors (169 LUADs and 107 LUSCs), to further validate our observations previously made within the lung adenocarcinoma and lung squamous cohorts of TCGA. This revealed significant concordance between the genes called as significant by the different approaches (among genes able to be tested in both cohorts, 20 of 28 had a *p* value < 0.05 in TRACERx). Additionally, the effect size of 5052 genes tested by *RVdriver* in both cohorts showed significant correlation in both LUAD (R = 0.55, *p* < 2.2e − 16) and LUSC (R = 0.53, *p* < 2.2e − 16), highlighting that the method is able to produce consistent calls across cohorts (Fig. 4E, F).

Finally, we assessed strategies for recovering cancer genes that are “missed” by *RVdriver*. Combining results from RVdriver and other tools generally resulted in an improved CGC score (Additional file 1: Figures S17A-C). Furthermore, when genes that were identified by two or more other tools, but not by RVdriver were considered in isolation and subjected to restricted hypothesis testing, 154 of 287 genes became significant at *q* < 0.25 (Additional file 1: Figure S17D).

### RNA VAF can predict driver and passenger mutations within established cancer genes

Given the ability of *RVdriver* to identify established and putative cancer genes, we investigated whether the underlying data, namely the RNA variant allele frequencies of somatic mutations, might enable functional driver mutations to be distinguished from non-functional passenger mutations in cancer genes. Distinguishing between driver and passenger mutations has been a significant research focus, and efforts such as Intogen have utilized machine learning approaches leveraging multiple features that describe tumorigenesis within different genes and cancer types. This has resulted in detailed atlases of probable driver mutations within putative cancer genes, including saturation analyses generated from next-generation DNA sequencing data that outperform experimental mutation saturation analyses, notably BoostDM [19].

**Fig. 5:**
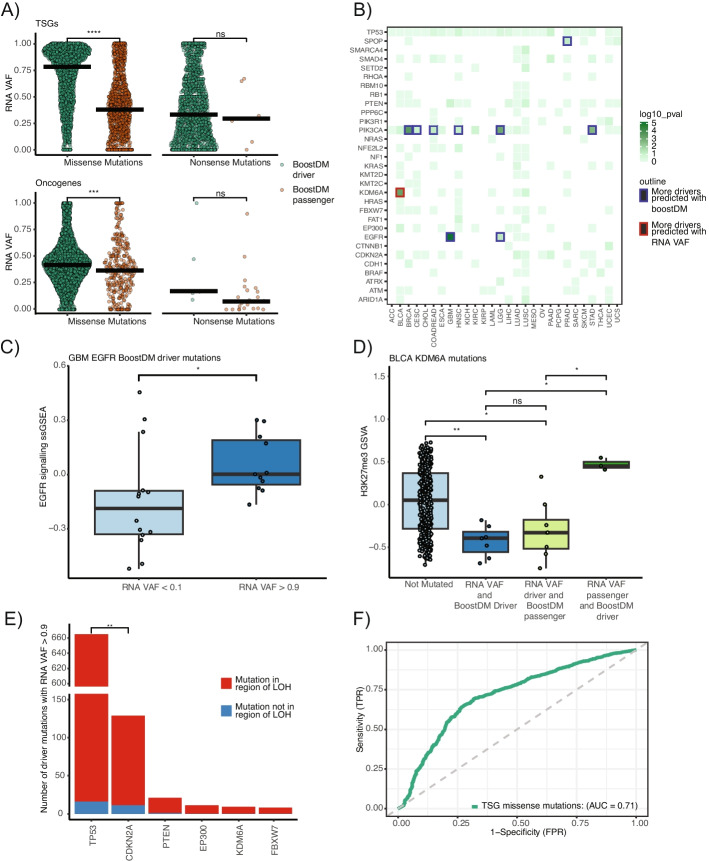
RNA VAF in driver and passenger mutations within established cancer genes.** A** RNA VAFs of mutations identified within a mutation saturation analysis of cancer genes [19]. Mutations were split by oncogene/tumor suppressor gene status, and whether they were missense or nonsense mutations. Mutations are colored by BoostDM driver status (green: BoostDM driver; orange: BoostDM passenger). **B** Focussed analysis of concordance of classification of driver and passenger mutations between BoostDM and RNA VAF. Genes harboring more (*p* < 0.1) mutations identified as drivers by BoostDM, and passengers by RNA VAF (i.e., having an RNA VAF < 0.1) have a blue outline. Tile color indicates the negative log of the *p* value for significance of Fisher’s exact test, comparing the number of driver mutations and passenger mutations identified within the gene by BoostDM and RNA VAF. Only genes mutated in >1 cancer type are shown. **C** ssGSEA scores for REACTOME “EGFR signalling in cancer” gene set among glioblastomas harboring a BoostDM driver, split by RNA VAF < 0.1 or RNA VAF > 0.9. Wilcoxon test was used to estimate significance. **D** ssGSEA scores for the “altered tri-methylation of lysine 27 on histone H3” gene set from Schlesinger et al. [42] among bladder carcinomas. These are split by whether they harbor a *KDM6A* driver mutation, as defined by BoostDM or RNA VAF (passenger: RNA VAF < 0.1 or driver: RNA VAF > 0.9). Wilcoxon test was used to estimate significance. **E** High RNA VAF (> 0.9) mutations within commonly mutated tumor suppressor genes. The majority of such mutations were found within a region of loss-of-heterozygosity (LOH). However, a small fraction of such instances are LOH-independent. More such instances were observed within *CDKN2A* than *TP53* (Fisher’s exact test). **F** RNA VAFs were computed for mutations identified within the BoostDM in silico saturation analysis of cancer genes. A total of 8833 mutations were expressed with more than 8 reads at that position. Scores from BoostDM were utilized as ground truth. Thus, an AUC of 1 would indicate perfect alignment between RNA VAF and BoostDM scores

We first sought to establish the degree to which RNA VAF was able to independently identify putative driver and passenger mutations as defined by BoostDM. RNA VAF was computed for each mutation with a minimum depth of 8 non-duplicated reads at that position within individual cancer types. Within missense mutations in tumor suppressor genes, there was a strong correlation between the driver/passenger annotation in BoostDM and the RNA VAF of the mutation, while a weak correlation was seen within such mutations in oncogenes (Fig. 5A). Similar relationships were not observed in nonsense mutations, in keeping with the tiny fraction of such mutations that represent probable passengers in tumor suppressor genes, or drivers in oncogenes.

Given that there are multiple competing factors that influence the RNA VAF of a somatic mutation, it is possible that further information of biological interest might be encoded within cases where there is disagreement between BoostDM and RNA VAF predicted driver mutations. We therefore sought to evaluate such cases. Among all missense mutations, 97 were predicted to be drivers by BoostDM, and high-confidence passengers by RNA VAF (i.e., RNA VAF less than 0.1), while the reverse was true (BoostDM passengers with RNA VAF greater than 0.9) in 93 cases.

We performed Fisher’s exact test for each cancer type to compare the numbers of putative driver and passenger mutations within each gene and cancer type identified by BoostDM and RNA VAF. Results for each gene and cancer type combination are displayed in Fig. 5C, where genes and cancer types for which BoostDM and RNA VAF disagreed about the numbers of driver and passenger mutations are outlined in blue or red.

There were nine instances where the RNA VAF classified significant numbers of mutations as passengers where BoostDM had classified them as drivers. All of these were in oncogenes. For example, of fifty-five BoostDM driver mutations within *EGFR* in glioblastoma multiforme, nineteen had RNA VAF < 0.1, compared to only eleven with RNA VAF > 0.9. This either suggests that these mutations are not bona fide drivers, or that within cancers harboring such a mutation, there is an absence of selective pressure for elevated expression of the mutated allele.

Within tumors containing a BoostDM *EGFR* driver mutation and an RNA VAF > 0.9, ssGSEA (single sample gene set enrichment analysis) revealed significantly higher expression of the REACTOME “EGFR signalling in cancer” gene set, when compared to BoostDM driver mutations with an RNA VAF < 0.1 (*P* = 0.025, Fig. 5C).

Similarly, six of the nine instances of RNA VAF repeatedly classifying BoostDM drivers as passenger mutations involved the gene *PIK3CA* (i.e., this happened separately across six cancer types). This either suggests that *PIK3CA* mutations are not bona fide drivers or adds to evidence supporting its activity acting in a dose-dependent manner, with cancers only being able to tolerate a certain level of mutated *PIK3CA* activity [43].

There was one instance (i.e., a combination of gene and cancer type) where RNA VAF repeatedly identified (Fisher’s exact test, *p* < 0.1) putative driver mutations among BoostDM passengers: *KDM6A* in urothelial carcinoma of the bladder. Here, it is plausible that BoostDM might have underestimated the putative functional impact of these mutations. In order to test this hypothesis, we computed ssGSEA scores for the impact of *KDM6A* across bladder tumors: altered tri-methylation of lysine 27 on histone H3 (H3K27me3)^38^. This analysis revealed a consistent trend towards reduced H3K27me3 gene set activity in tumors with mutations that BoostDM and RNA VAF agreed were drivers (i.e., RNA VAF > 0.9), relative to those without *KDM6A* mutations (Fig. 5D). However, where RNA VAF disagreed with BoostDM, gene set activity suggested that RNA VAF results tended to be more consistent with tumor phenotype than BoostDM (i.e., in BoostDM passengers that were RNA VAF drivers, H3K27me3 activity was similar to BoostDM drivers that were RNA VAF drivers; while in BoostDM drivers that were RNA VAF passengers, H3K27me3 activity was more similar to non-*KDM6A*-mutated tumors).

Significantly elevated RNA VAF (i.e., > 0.9) in tumor suppressor genes is consistent with non-expression of the wild-type allele. Canonically, this can result from loss-of-heterozygosity (LOH) of the wild-type allele. However, allele-specific copy-number profiling from SNP array data allows identification of instances where wild-type allele inactivation occurs independently of LOH (i.e., its expression is repressed). The frequency of LOH-independent inactivation of the wild-type allele was compared across tumor suppressor genes. While for many genes there were not sufficient available mutations to power this analysis, this did reveal that LOH-independent wild-type allelic inactivation was more common in *CDKN2A* than *TP53* (Fig. 5E).

Finally, we evaluated the performance of RNA VAF as a metric for identifying driver mutations within cancer genes by comparing its sensitivity and specificity to scores from BoostDM from Intogen as a set of true positive calls.

We considered all (8833) mutations where RNA coverage was 8 or more reads within the 68 genes analyzed by BoostDM, using the median RNA VAF of mutations that recurred within cancer types. The AUC for all mutations was 0.58. However, this was not evenly distributed across all mutation types. For example, the vast majority of nonsense mutations within tumor suppressor genes were considered by BoostDM to be driver mutations. However, for missense mutations within tumor suppressor genes, where functional impact is typically less certain, the AUC of RNA VAF was 0.71 (Fig. 5F). This indicates that RNA VAF encodes significant information about the functional impact of mutations within cancer genes. Of note, within oncogenes the AUC for missense mutations was 0.53, and for nonsense mutations it was 0.79. Overall, this analysis highlights that RNA VAF might be more useful in predicting putative driver mutations within tumor suppressor genes than oncogenes.

## Discussion

The identification of cancer genes has been an important endeavor, leveraging patterns of mutagenesis within tumor genomes to identify signals of positive selection and recurrence. However, to date, the role for bulk RNA sequencing in this process has been limited.

Here, we have proposed a novel approach for the identification of cancer genes. *RVdriver* utilizes RNA variant allele frequencies from somatic mutations and identifies clear signals in patterns of mutation expression between cancer genes and non-cancer genes. It exhibits comparable performance to DNA-based tools and importantly identifies putative novel cancer genes, not highlighted to date by DNA approaches, that might merit further investigation.

Certain cancer genes appear more amenable to discovery by *RVdriver* than DNA-based tools. For example, *ERBB3*, which is an established cancer gene, is identified as such by *RVdriver* in breast invasive carcinomas, stomach adenocarcinomas, and uterine corpus endometrial carcinomas, but not by DNA-based tools in any of those instances. Indeed, *RVdriver* highlights 23 instances of genes within the COSMIC cancer gene census list acting as cancer genes within cancer types which are not picked up by DNA approaches. Similarly, *RVdriver* uniquely identifies 33 instances of genes outside of the COSMIC cancer gene census list acting as cancer genes. While it is possible that some such genes, particularly those called with lower confidence, might represent false positives. Other candidates, such as *SMARCA1*, *KDM6B*, *IRF2*, *PLK4*, and *HUWE1*, may well have functional relevance to cancer development in particular cancer types, and be worthy of further investigation.

*SMARCA1* was detected in kidney papillary cell carcinoma (KIRP) and liver hepatocellular carcinoma (LIHC). *SMARCA1* is a member of the SWI/SNF chromatin remodelling complex, which regulates the transcription of genes by altering chromatin structure. Other members of the SWI/SNF complex, namely *ARID1A/B* and *ARID2* are well-established tumor suppressor genes [24]. Epigenomic disruption is common in both KIRP and LIHC. In KIRP, mutations in chromatin remodelling genes occur in ~ 53% of tumors [44]. Similarly, in LIHC, ARID1A (7%), ARID2 (5%), and BAP1 (5%) mutations are frequently observed [45]. The presence of putative, functional *SMARCA1* mutations in both cancer types might point to a broader disruption of chromatin remodelling driving tumor development through altered gene expression in tumor cells.

*HUWE1* was detected in head and neck squamous cell carcinoma (HNSC) and bladder adenocarcinoma (BLCA). *HUWE1* (HECT, UBA, and WWE domain containing E3 ubiquitin protein ligase 1) encodes an E3 ubiquitin ligase that regulates the degradation of multiple proteins including *MYC*, *TP53*, and components of the *WNT* signalling pathway. As such, it has been implicated in regulation of genomic stability and cellular proliferation [46, 47] and has previously been suggested to be a tumor suppressor gene specifically in HNSC [24].

*IRF2* was detected in LIHC. *IRF2* (Interferon Regulatory Factor 2) disruption has been associated with immune evasion phenotypes through a lack of MHC-I antigen presentation [48] and was detected as a pan-cancer tumor suppressor gene in Bailey et al. [24]. LIHC tumors frequently arise in the context of chronic inflammation driven by factors such as viral infections (e.g., hepatitis B and C) and alcohol consumption [49]. Mutations in genes regulating the immune response such as *IRF2* may weaken immune surveillance in this inflammatory environment, facilitating tumor formation and progression.

*PLK4* was detected in lung squamous cell carcinoma (LUSC). *PLK4* (Polo-Like Kinase 4) is a member of a broader family of genes which are critical regulators of the cell cycle, with *PLK4* required for centriole replication [49, 50]. Overexpression of PLK4 has been shown to induce supernumerary centrosomes, driving genomic instability and an invasive phenotype [51]. LUSC tumors are characterized by extreme genomic instability, with genome doubling events occurring in 83% of LUSC tumors within the TRACERx 421 cohort [52]. Activating mutations of *PLK4* may contribute to this instability, facilitating tumorigenesis and progression by enabling chromosomal missegregation and aneuploidy.

Interestingly, 15 out of the 56 uniquely identified gene-cancer type combinations are found on the X chromosome, with 6 out of 13 of such genes being within the COSMIC cancer gene census. This is a significant enrichment compared to the DNA-based approaches, suggesting *RVdriver* is able to capture a signal specific to this chromosome, plausibly relating to X chromosome inactivation, for the detection of cancer genes.

Conversely, *RVdriver* fails to identify a number of established cancer genes in certain instances. While this clearly, and perhaps unsurprisingly, indicates that *RVdriver* is not a panacea in terms of its ability to identify cancer genes, it might also provide insight into important tumor biology. For example, *RVdriver* fails to identify *PIK3CA* as a putative cancer gene in thirteen tumor types in which it is identified by DNA approaches, and only successfully identifies it as such in three cancer types. The possibility that *PIK3CA* might represent an outlier in terms of its patterns of mutation expression is supported by orthogonal analysis, in which *PIK3CA* mutations across six cancer types exhibit low RNA VAF despite being predicted by BoostDM to be driver mutations. This highlights that *RVdriver* can reveal facets of tumor biology (in this case, plausibly the previously reported lack of tolerance of constitutively active *PIK3CA* signalling [43]) that might be missed by existing approaches.

*RVdriver* does not, then, provide an exhaustive list of cancer genes. However, our analysis suggests that through combining its results with those of other tools, a more comprehensive list would be defined. Importantly, this relationship appeared to be reciprocal, with *RVdriver* adding value (as measured with the CGC score) to other approaches. Similarly, bigger cohorts might help to uncover other bona fide cancer genes which approached significance in our analysis.

We also demonstrate the RNA VAF of mutations can highlight differences between driver and passenger mutations among missense mutations within tumor suppressor genes. It could therefore act as an important orthogonal adjunct to attempts seeking to distinguish between functional and non-functional mutations in cancer. Indeed, by phenotyping tumors according to relevant gene set expression scores, we show that RNA VAF identifies instances of possible driver-passenger misclassification events within the cancer genes *EGFR* and *KDM6A*.

The differences in the ability of RNA VAF to identify driver mutations among oncogenes and tumor suppressor genes might hold important implications for *RVdriver*. It suggests there may be asymmetry in its ability to identify putative oncogenes and putative tumor suppressor genes. Further work could characterize the relative ability of different tools to identify oncogenes and tumor suppressors; combinations leveraged strategically could help to maximize the biological insight from these approaches.

RNA VAF likely reflects the activity of different processes in tumors. First, somatic mutations that are more likely to be associated with a clonal sweep (i.e., driver mutations) are more likely to be present in a greater fraction of cancer cells, and hence exhibit higher RNA VAF. Second, in many oncogenes, amplification of the mutated allele is associated with increased oncogenic activity; while retention of a mutated allele in the context of a loss-of-heterozygosity is classical in tumor suppressor genes. Third, in tumors, expression preferentially is derived from the tumor component of the admixed sample [30], and particularly so for many cancer genes which are often highly expressed in epithelial tissues. Fourth, non-mutational mechanisms underpinning allele-specific expression, such as methylation, might plausibly favor the non-mutated allele in the presence of a driver mutation [33].

## Conclusions

Irrespective of the mechanism underpinning this phenomenon, we have presented a simple approach, encapsulated in *RVdriver*, that adds value to analyses seeking to identify new cancer genes. This may be of value to genomic-transcriptomic analyses of cancer genes; and future studies should also attempt to interrogate the genome and transcriptome in parallel to understand in full the landscape of functional variation in tumors.

## Methods

### Determining the RNA VAF of somatic mutations

Each BAM file was first pre-processed following GATK’s best practices, marking and removing duplicate reads and applying base quality score recalibration (GATK v4.1.7.0) using default parameters. After lifting over the position (using the liftOver R package) of each mutation from hg19 to hg38, the RNA reference and alternate counts for each position containing a single-nucleotide variant were calculated using the bam2r function from the R package deepSNV using default parameters (v1.42.0) [53]. The RNA variant allele frequency was calculated as the sum of non-duplicated reads aligning to the alternate allele divided by the sum of non-duplicated reads aligning to both the reference and alternate alleles at that position.

### RVdriver

RVdriver was run separately on individual cancer types and involved the following steps.

1. Mutation table preprocessing:
  a Filter the MAF file for synonymous and nonsynonymous single-nucleotide variants only (non-synonymous mutations comprised nonsense, missense, splice site, and non-stop mutations).
  b Detect and remove dinucleotide/trinucleotide mutations.
  c Limit the number of mutations per gene, per sample (default: 3).
  d Scale the RNA VAF of nonsense mutations to account for nonsense-mediated decay (see NMD scaling).
  e Assign a weight to each mutation based on the RNA depth at the mutated position. This mitigates against potential false positives arising from mutations in lowly expressed genes. Additionally, to prevent biasing towards extremely highly expressed genes, the RNA depth of mutations are capped at the 90th percentile. Then the weight is calculated as:

$$log\_coverage = log2(capped\_RNA\_depth + 1)$$

$$mutation\_weight = log\_coverage / max(log\_coverage)$$

Finally, to ensure the RNA VAFs at positions with 0 RNA coverage still contribute to the model fitting, these mutations are assigned the same weight as a mutated position with an RNA depth of 1 non-duplicated read.

f Calculate which genes to test within a given cancer type. A gene was tested if, within a single cancer type, it had > 1 non-synonymous mutations at an RNA depth > 7 non-duplicated reads.
g The observed RNA VAFs within the background set of synonymous mutations differ systematically from the nonsynonymous mutations within genes analyzed by *RVdriver.* To address this, we calculate a synonymous mutation scaling factor. Firstly, we calculate the mean RNA VAF of nonsynonymous mutations in each gene being tested (calculated in step f), that are not CGC genes. This distribution of mean RNA VAFs is then compared to the RNA VAFs of all synonymous mutations that will contribute to the background (i.e., those with > 7 non-duplicated reads). The scaling factor is then determined by finding an optimal value (*k*) that minimizes the differences between the mean and median RNA VAFs of the synonymous and nonsynonymous mutations. The scaling factors applied to the TCGA cohorts can be found in Additional file 3: Supplementary Table 2.
h To determine the number of synonymous mutations to be sampled per nonsynonymous mutation in the genes of interest, an exponential function “rate” is derived. This rate is computed such that, for a gene mutated in 5% of the cohort, 10 synonymous mutations will be sampled per nonsynonymous mutation.

$$rate = log(10) / 0.05 * cohort\;size$$

To avoid an extreme rate in smaller cohorts, the rate is capped at 0.202 (this is the highest value that ensures 1 synonymous mutation is sampled per non-synonymous mutation when only 2 mutations are present within a gene across the cohort. The minimum of the above calculation and 0.202 is taken.

The rate is then applied to each test as described below.

(2) Iterate through each gene being tested, performing the following.
  a Filter for all non-synonymous mutations within the gene of interest
  b Calculate the number of synonymous mutations to be sampled per nonsynonymous mutation.

$$n=min(round(exp(rate\times n_{samples}),\;2\times n_{samples})$$

where $rate$ is the cohort specific rate described above and ${n}_{samples}$ is the number of samples with a nonsynonymous mutation in the gene being tested. The number of synonymous mutations sampled is capped at the minimum of the exponential curve function or double the number of mutated samples.

c. Sample *n* number of synonymous mutations without replacement (at an RNA depth > 7 non-duplicated reads) from each tumor sample containing a nonsynonymous mutation within the gene of interest. The sampling process uses the following hierarchical stepwise approach:
  i. Consider synonymous mutations only within the tumor harboring a mutation within the gene of interest.
  ii. Consider synonymous mutations within all tumors harboring a mutation within the gene of interest.
  iii. Consider all synonymous mutations from tumors within the cancer type being considered.
  iv. Consider mutations of the same type (e.g. missense mutations) from within that sample.
  v. If sufficient mutations have not been sampled at this point, proceed with the incompletely sampled background.
  vi.  Optionally, any synonymous mutations from the gene of interest can also be included in the background.

Additional file 1: Figure S8 summarizes the proportions of tests where each of these sampling steps are required.

This approach ensures that, where possible, we control for differences in sample-sample variables such as estimated tumor content as well as differences in overall expression from the tumor compartment relative to the non-tumor compartment of the admixed bulk sample.

d. Generate a weighted linear model comparing the RNA VAF distribution of nonsynonymous mutations within the gene of interest against the background set of synonymous mutations using the following formula:

$$lm(RNA\_VAF\sim mutation\_function, weights = mutation\_weight)$$

This model includes a one-sided test to assess the directional relationship between the RNA VAF of the nonsynonymous mutations compared to the background.

e Bootstrap the sampling and model fitting 25 times to limit the impact of sampling given RNA VAFs are variable across mutations.
f Derive a *p* value by computing the geometric mean of the *p* values of the 25 previous tests.
g In cases where a gene has a sufficient number of multiple mutation types, e.g., nonsense and missense mutations, then these are tested separately, as well as together.

For the cancer type-specific analyses presented in the manuscript, a single gene-cancer type p-value was derived by taking the lowest p-value of the tests performed in step g. For the pan cancer analysis (Additional file 1: Figure S14), the p-value was taken from the test where all gene mutations were tested together. Multiple hypothesis testing correction was then performed using the Benjamin Hochberg method. A gene is classified as a putative driver if the q value is < 0.25.

Code to run RVdriver is available here: https://github.com/McGranahanLab/RVdriver.

### NMD scaling

Premature termination codons can result in the production of truncated proteins or the degradation of messenger RNAs by NMD. To account for NMD, we scaled the observed RNA VAFs of nonsense mutations by the degree in which it was predicted to lead to NMD. The degree of NMD, or “NMD score” for a given mutation was derived from a previously published approach [38]. Then, nonsense mutations were binned into deciles of NMD score, and the mean RNA VAF from mutations within each decile was divided by the mean RNA VAF of missense mutations to derive a nonsense mutation scaling factor. The differences in NMD scores across the TCGA cohort and its influence on RNA VAFs of nonsense mutations can be found within Additional file 1: Figures S9A and S9B.

### Classification of known cancer genes

Cancer genes were classified as those present in the Cancer Gene Census (CGC) list (v.96) [27]. This classification was used in the analyses contained within Figs. 1, 2, and 3.

Oncogenes and tumor suppressor genes (TSGs) were assigned as outlined in a previous pan-cancer analysis [24]. In cases where a gene had a cancer-specific oncogenic or tumor suppressive function, this was prioritized over the pan cancer assignment for that gene [24]. This classification was used within the analyses conducted within Figs. 1, 3, and 4.

### Somatic copy number assignment

Affymetrix SNP 6.0 arrays were downloaded from the TCGA (see above). ASCAT was run to obtain allele-specific copy numbers as well as purity and ploidy estimates for each sample [36].

### Estimating the cancer cell fraction of mutations

The cancer cell fraction (CCF) was estimated using the following formula as per McGranahan et al. [16].

$$VAF/p\:\ast\:((1\:-\:p)\:\ast\:Ncn\:+\:(p\:\ast\:Tcn))$$

where *p* is the tumor purity derived using ASCAT, *Ncn* is the normal copy number at the mutated position (assumed to be 2 within this analysis), and *Tcn* is the copy number within the tumor at the mutated position, derived from ASCAT. Point estimates for CCF and their associated confidence intervals were then calculated using a binomial distribution as previously described [16]. A mutation was defined as clonal when the 95% confidence interval overlapped 1, and subclonal when this was not the case.

### Estimating the mutant copy number fraction

The mutant copy number (the number of chromosomal alleles harboring the mutation) was estimated using the following formula.

$$({VAF}/{p})*(({p }*{ CNt}) +{ Ncn}* (1 -{ p}))$$

The mutant copy number fraction was estimated by dividing the mutation copy number by the total copy number within the tumor at the mutated position, and capped at 1.

### Identifying allele-specific expression

Allele-specific expression was calculated using the following approach. In accordance with the expected distribution of allelic expression, a beta-binomial test was used to test for allele-specific expression [54] using the pbetabinom function from the R package VGAM (v1.1.1) [55] and an over-dispersion parameter of 0.05. To assess the probability of observing allele-specific read counts at least as disparate as the observed distribution given the estimated mutant copy number and the total copy number, the following beta binomial test was performed.

$$Betabin(X\:\geq\:m,\;t,\;CPNratio)$$

where *m* represents the RNA-seq read counts of the mutated allele, *t* is the total RNA reads at that position and CPNratio is the estimated mutant copy number divided by the total copy number at the mutated position. Genes with a *p* value < 0.05 from this test were considered to show copy number independent allele-specific expression.

### DNA-based tools to detect cancer genes

Six different tools utilizing patterns observed within genomic data were used to benchmark the performance of RVdriver. All 6 tools were run on default settings and on all mutations available for a given sample.

1. DIGdriver builds genome-wide maps of somatic mutation rates in cancer genomes to test for an excess of observed mutations compared to the expected numbers based on a neutral mutation rate [8].
2. dNdScv looks for excess numbers of nonsynonymous mutations at nonsynonymous sites compared to synonymous mutations at synonymous sites to infer positive selection for mutations in putative cancer genes [5].
3. OncodriveFML uses scores for the deleteriousness of mutations (in this case CADD scores [56]) to identify genes where there are an excess of more functional mutations in a gene than would be expected by chance [15].
4. OncodriveCLUSTL looks for clustering of mutations in specific hotspots within a given gene, identifying putative drivers as those that have an increased number of hotspots than would be expected by chance [10].
5. MutPanning identifies cancer genes as those with an excess number of mutations above the background rate as well as an excess number of mutations in unusual trinucleotide contexts [7].
6. MutSig2CV identifies genes mutated more often than expected by chance given cancer-specific, inferred background mutation processes. In this analysis, only the “CV” (abundance) measure of significance was utilized.

When benchmarking RVdriver against these tools, we used *q* value thresholds to call putative driver genes as outlined in each respective publication. These were as follows:

- DIGdriver: q = 0.01
- dNdScv: q = 0.05
- oncodriveFML: q = 0.25
- oncodriveCLUSTL: q = 0.05
- MutPanning: q = 0.25
- MutSig2CV: q = 0.1

We used the R package ComplexUpset [57] (v1.3.3) to investigate the overlap between putative cancer-type-specific cancer genes determined by each tool at their respective *q* values. We split this analysis to investigate the overlap between each tool within cancer genes as defined by the CGC list and non-CGC list genes.

A “CGC score” for each DNA-based tool and RVdriver was computed as described by a previous approach [39]. Briefly, the CGC score was generated using the following equation:

$$E(R)={\Sigma }_{i=1}^{N} \frac{Pi}{log(i+1)}$$

where $Pi$ is the proportion of genes with greater significance than $i$ which are within the CGC list. *N* is a user-defined threshold to consider only “top ranked” genes. For our pan-cancer plot (Fig. 2D), we computed the CGC score using *N* = 250. For the cancer type-specific CGC scores used in Additional file 1: Figures S17B and S17C, *N* was set to 40. In cancer types where RVdriver tested fewer than 40 genes, *N* was set as the total number of genes tested within that cancer type.

### Validation of RVdriver using CancerMine

We leveraged CancerMine [40] to evaluate the degree of experimental and clinical literature support for significant hits from *RVdriver.* A comprehensive list of cancer genes described in literature was downloaded from Zedono at 10.5281/zenodo.7689627 [58] (v50, cancermine_collated.tsv). The cancer types were edited to fit those found in the TCGA, and literature support was stratified into the following categories. (i) CGC gene; (ii) non-CGC gene with literature support in the cancer type of interest; (iii) non-CGC gene and literature support in any cancer type; (iv) no support.

### Validation of RVdriver using gnomAD

Gene level constraint data was downloaded from https://gnomad.broadinstitute.org/downloads#v2-constraint. As suggested by the gnomAD group, the observed / expected upper bound fraction score was used as a measure of the level of germline intolerance for missense and nonsense mutations. As it is somewhat dependent on gene and sample size, the use of the upper bound fraction ensures the score is a conservative estimate of the observed / expected ratio. For each tool, the mean observed / expected upper bound fraction was calculated for all non-CGC genes detected uniquely by that tool.

### Validation of RVdriver using TRACERx

Mutation level data for the TRACERx 421 cohort were generated as previously described [22]. To make this multi-region dataset compatible with *RVdriver*, a single region was randomly selected to represent a given sample. This resulted in a cohort of 107 LUSC and 169 LUAD tumors, with 32,543 and 54,920 mutations respectively. *RVdriver* was then applied to this cohort in the same way as described above.

### Driver annotation using BoostDM

boostDM is a machine learning method for in silico saturation mutagenesis. Utilizing different features of mutations it has generated a cancer-type-specific atlas of probable driver and passenger mutations [19]. This pan-cancer atlas was downloaded (https://www.intogen.org/boostdm/downloads) and edited to match tumor types with those present within our dataset. This resulted in a mutation table of 10,608 SNVs with matched, tumor type-specific, boostDM driver/passenger annotation (binarized about a boostDM score of 0.5).

### Gene set enrichment analysis

To perform geneset enrichment analysis, BLCA and GBM expression count tables were downloaded from the TCGA portal (see above). Lowly expressed genes (where > 20% of the cohort had < 5 counts) were removed. This filtered counts table was then transformed into VST counts using the vst command from the R package DESeq2 [59] (v1.34.0).

To investigate the activity of EGFR signalling in GBM and KDM6A in BLCA, the reactome gene sets “REACTOME_SIGNALIN_BY_EGFR_IN_CANCER” and “SCHLESINGER_H3K27ME3_IN_NORMAL_AND_METHYLATED_IN_CANCER” [42] were downloaded respectively from the reactome pathway knowledgebase [60]. We performed a single sample GSEA (ssGSEA) for the above gene sets using the R package fgsea (v1.10.1) [61] on VST counts using a Gaussian distribution and with default parameters.

### Analyses

RVdriver was run in the R environment (v4.2.3). RVdriver was run using tidyverse (v2.0.0), argparse (v2.2.2), ggplot2 (v3.4.2), and ggpubr (v0.6.0).

All plots were generated in the R environment (v4.4.2) using ggplot2 (v3.5.1), cowplot (v1.1.3), ggpubr (v0.6.0), ggbreak (v0.1.4), patchwork (v1.3.0), ROCit (v2.1.2), ComplexUpset (v1.3.3), ggforce (v0.4.2), ggsignif (v0.6.4) and MoMAColors (v0.0.0.9000). All Wilcoxon tests performed are two-sided, using the function wilcox.test() in base R, with the effect size obtained using wilcox_effsize() from the package rstatix (v0.7.2).

## Supplementary Information

Additional file 1: Supplementary Figures

Additional file 2: Supplementary Table 1

Additional file 3: Supplementary Table 2

Additional file 4. Review history

## Data Availability

RNA sequencing data (BAM files and processed gene expression counts) for 8053 samples across 31 cancer types was downloaded via the GDC data portal (https://portal.gdc.cancer.gov/) using the GDC data transfer tool. For a full list of samples and the specific RNA-seq bam file used in this analysis, see Additional file 2: Table S1. A publicly available mutation annotation file (MAF) file compiled by the MC3 working group [25] was downloaded (https://gdc.cancer.gov/about-data/publications/mc3-2017). This MAF file was filtered to include only the samples investigated in this study (Additional file 2: Table S1). In addition, variants assigned as “PASS” were selected, removing potentially artifactual variants. In ovarian cancer (OV) and acute myeloid leukemia (LAML), variants that had only the “wga” filter were also kept as samples within this cancer type were primarily sequenced using whole genome amplification. This is in keeping with previous work [24]. Samples that had been excluded from previous studies based on pathology review, samples excluded from previous studies as “hypermutators” or samples flagged as having degraded RNA were also removed [24]. Finally, mutations lying within the ENCODE blacklisted regions were also removed [26]. The RNA VAFs for all 911,592 single-nucleotide variants across the 7882 tumors, as well as all the other data required to generate the analyses presented in this manuscript can be found on Zenodo at 10.5281/zenodo.14679440 [62]. The code used in the manuscript, including the code to run RVdriver, can be found at https://github.com/McGranahanLab/RVdriver [63] and is released under the 3-Clause BSD License. A copy of the code is also deposited on Zenodo [62]. CancerMine data (v50, cancermine_collated.tsv) was downloaded 18th October 2023 from 10.5281/zenodo.7689627 [58]. TRACERx data was downloaded from 10.5281/zenodo.7603386 [64].
