# Supplementary material for: Cancer gene identification from RNA variant allelic frequencies using RVdriver
Source: Genome Biol. 2025 Jun 13;26:165. doi: 10.1186/s13059-025-03557-y (PMC12164115; doi:10.1186/s13059-025-03557-y)
Supplement: Supplementary file 1 — Additional file 1: Supplementary Figures [file 13059_2025_3557_MOESM1_ESM.pdf]

Figure S1

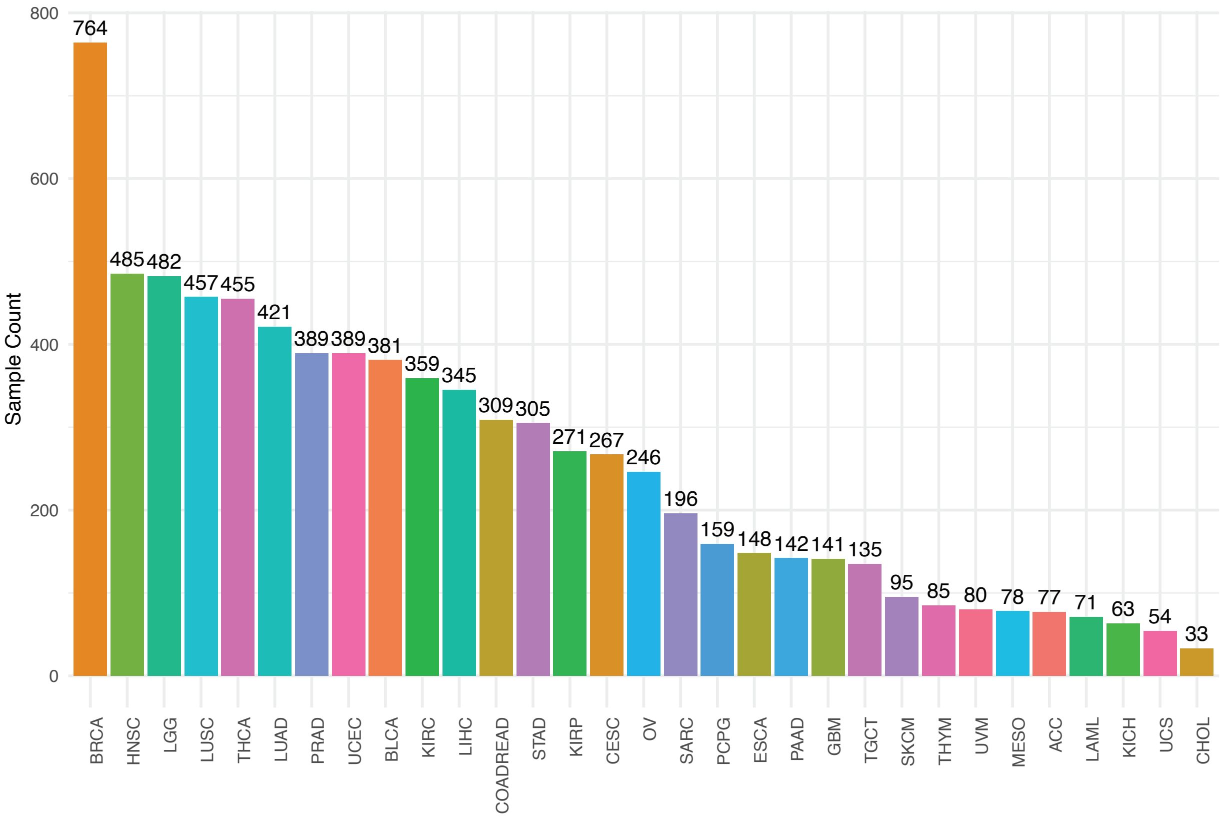

Figure S1: Cohort overview. Bars represent the number of samples with paired DNA/RNA-sequencing data across the available cancer types within the TCGA cohort.

Figure S2

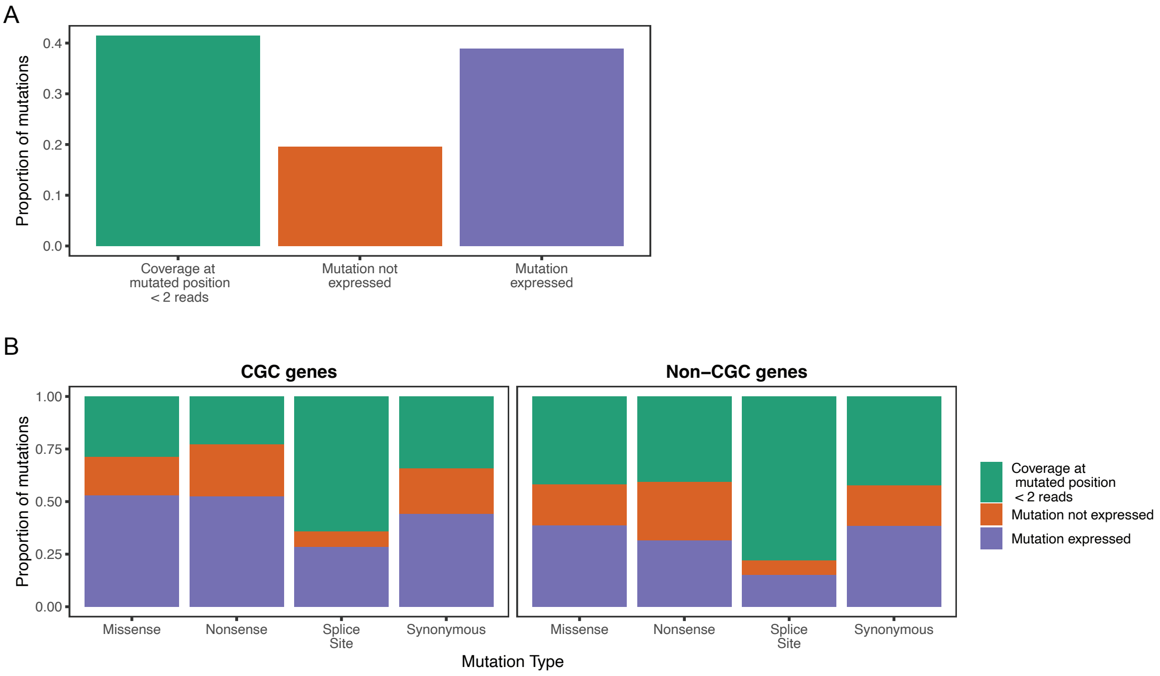

Figure S2: A. Proportion of expressed mutations across all mutations within the cohort mutation table. A mutation was considered expressed when the alternate allele had 2 or more non-duplicated reads aligned at the mutated position. A mutation was considered non-expressed when the total RNA depth at the position was >= 2 non-duplicated reads and the RNA alternate read count was < 2 non-duplicated reads. The gene in which the mutation was found was considered not expressed when the RNA depth at the mutated position was < 2 non-duplicated reads. B. Portion of expressed mutation across different mutation types and across genes present or not present in the Cancer Gene Census list. Definitions for mutant expression are the same as above.

Figure S3

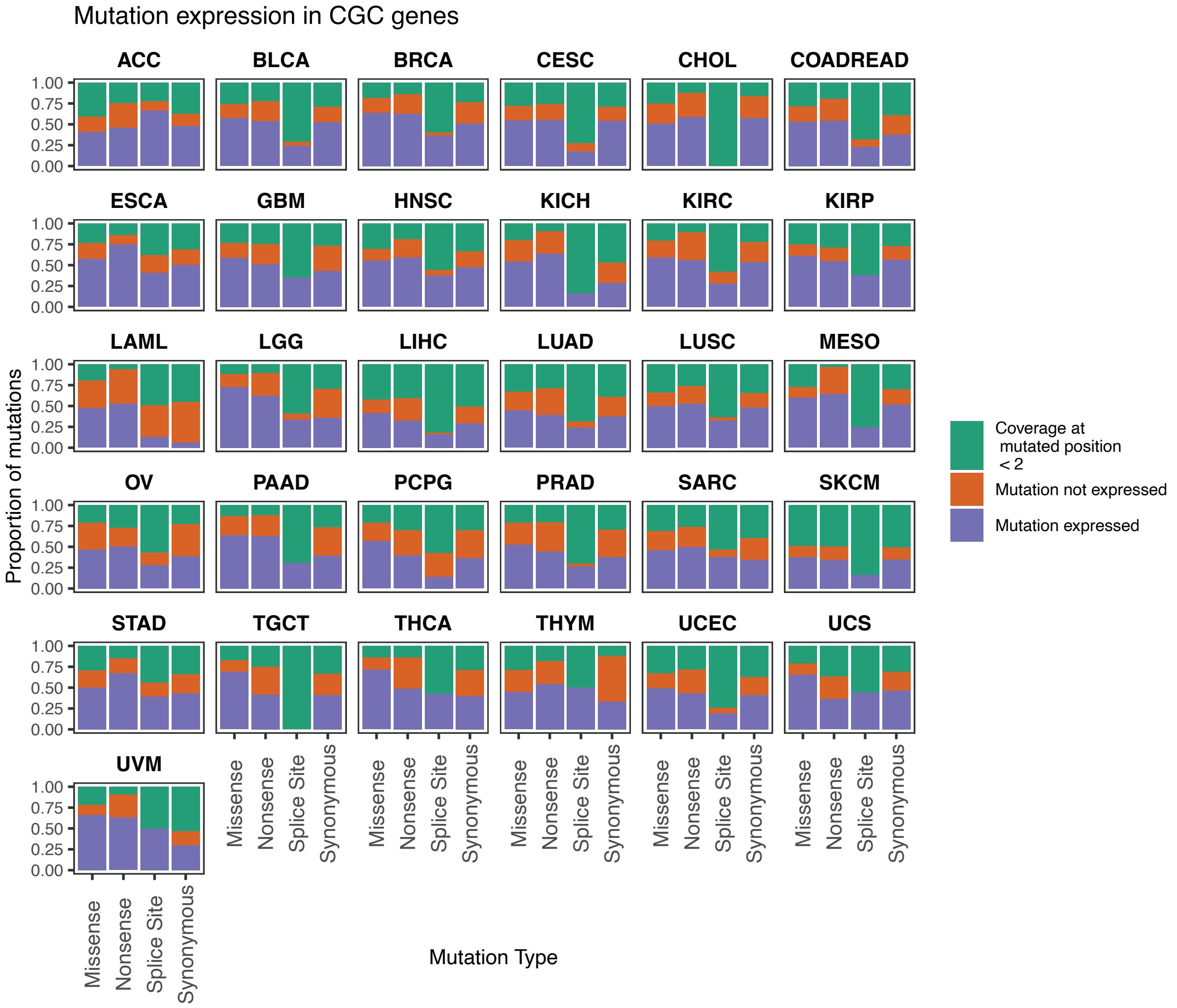

Figure S3: Proportion of expressed mutations within genes in the COSMIC cancer gene census list, and across different mutation types and cancer types. Definitions for mutant expression are the same as figure S2.

Figure S4

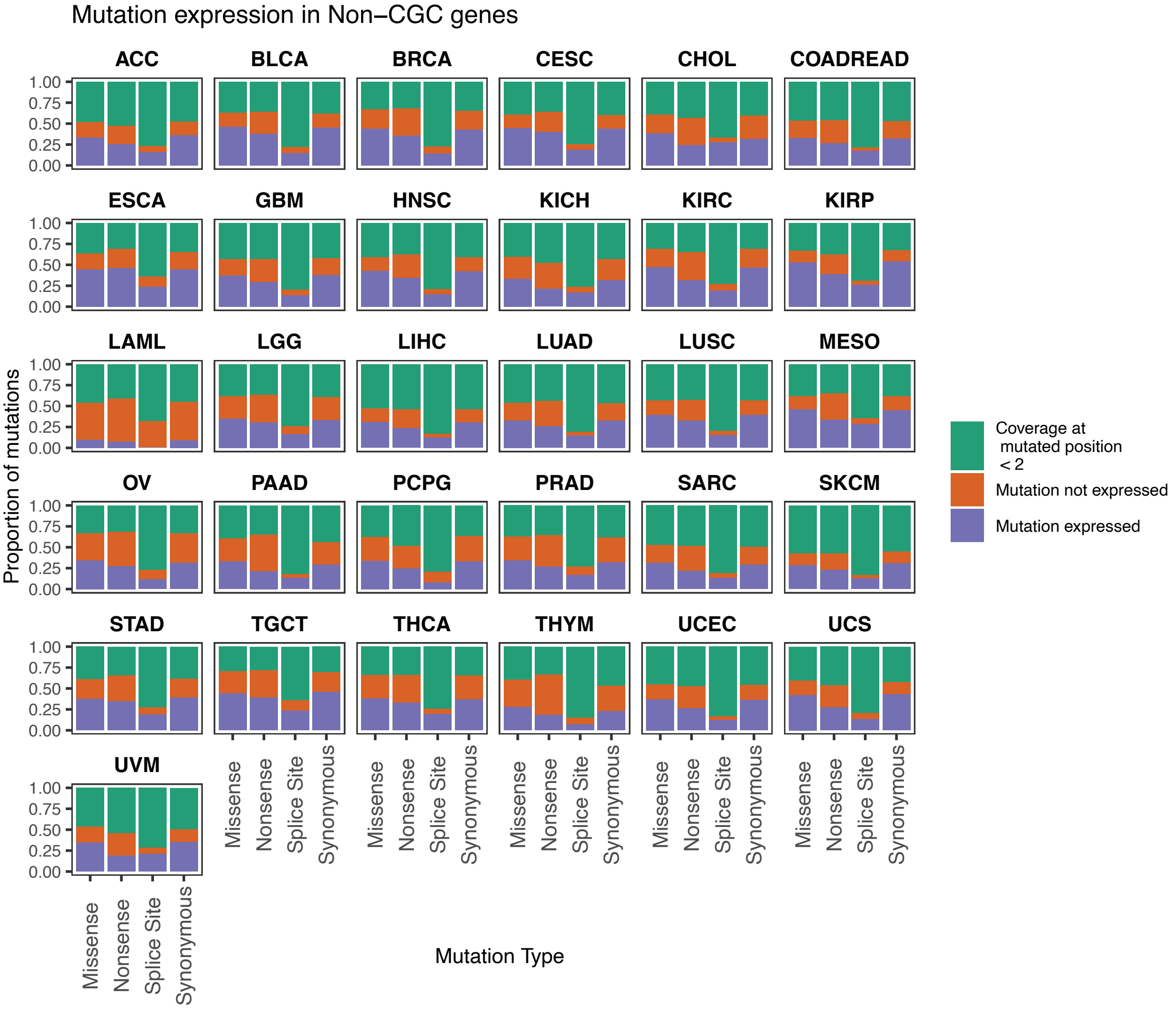

Figure S4: Proportion of expressed mutations within genes not in the COSMIC cancer gene census list, and across different mutation types and cancer types. Definitions for mutant expression are the same as figure S2.

Figure S5

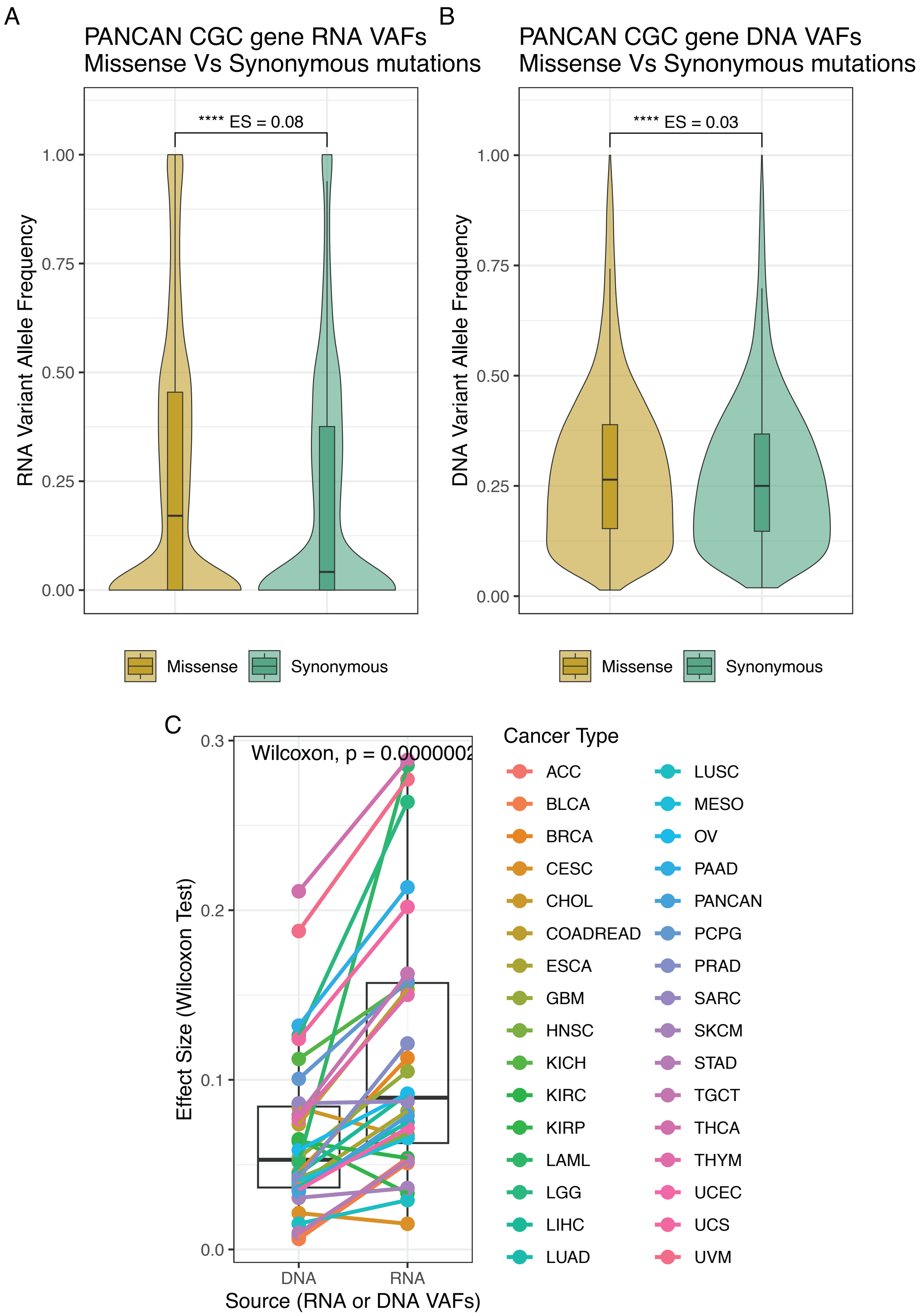

*Figure S5: Comparison of RNA and DNA VAFs between missense and synonymous mutations in CGC genes across cancer types in the TCGA cohort. A,B. Pan Cancer RNA VAFs (A) and DNA VAFs (B) of synonymous versus missense mutations within COSMIC cancer gene census genes; **** indicates p<0.0001; Wilcoxon test. ES: effect size. C. Paired comparison of Wilcoxon test effect sizes across multiple cancer types, comparing RNA VAFs (right) and DNA VAFs (left). Each line represents a different cancer type.*

Figure S6

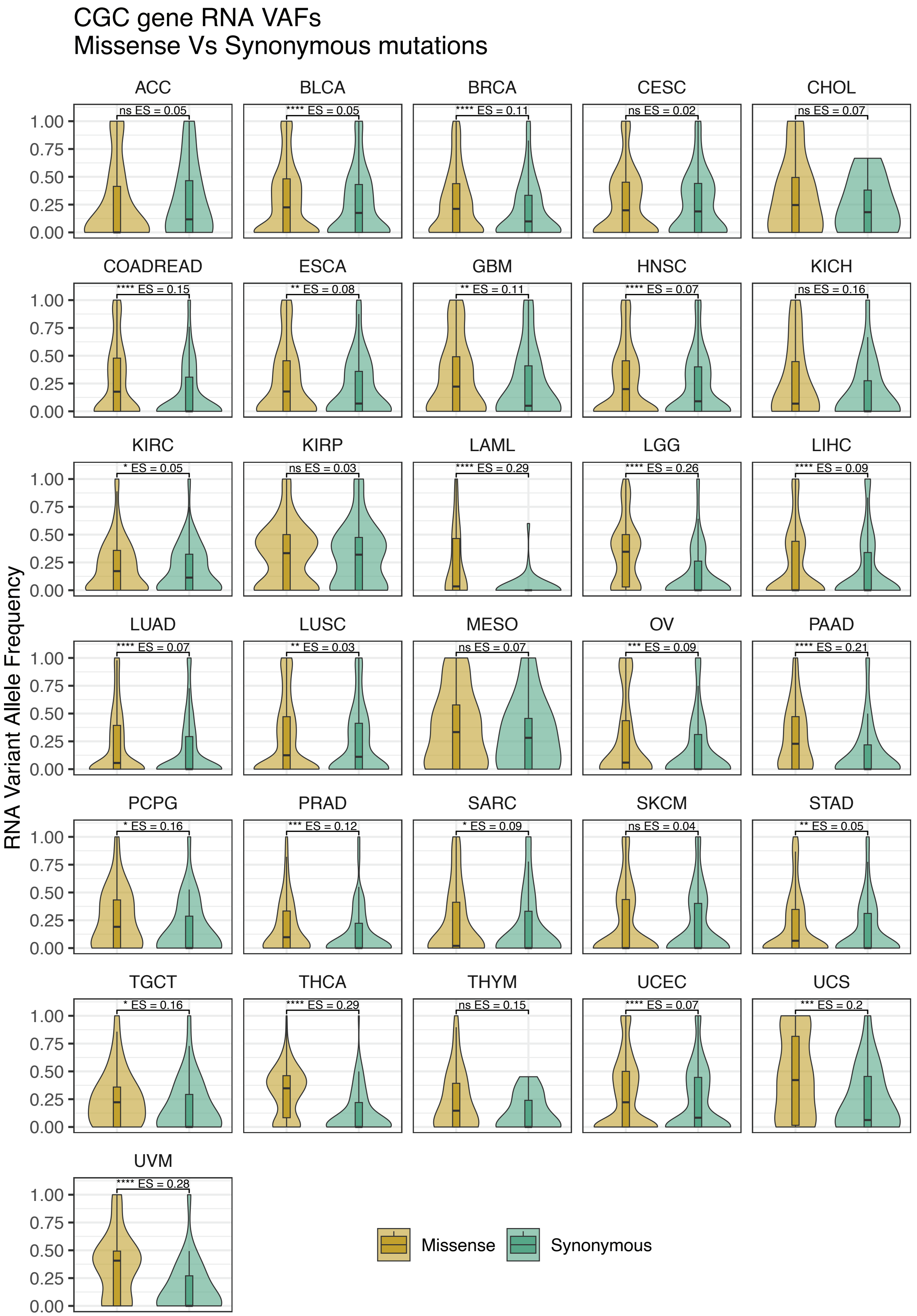

*Figure S6: Comparison of RNA VAFs between missense and synonymous mutations in CGC genes across individual cancer types in the TCGA cohort; ns indicates p > 0.05, * indicates p < 0.05, ** indicates p < 0.01, *** indicates p < 0.001, **** indicates p < 0.0001; Wilcoxon test. ES: effect size*

Figure S7

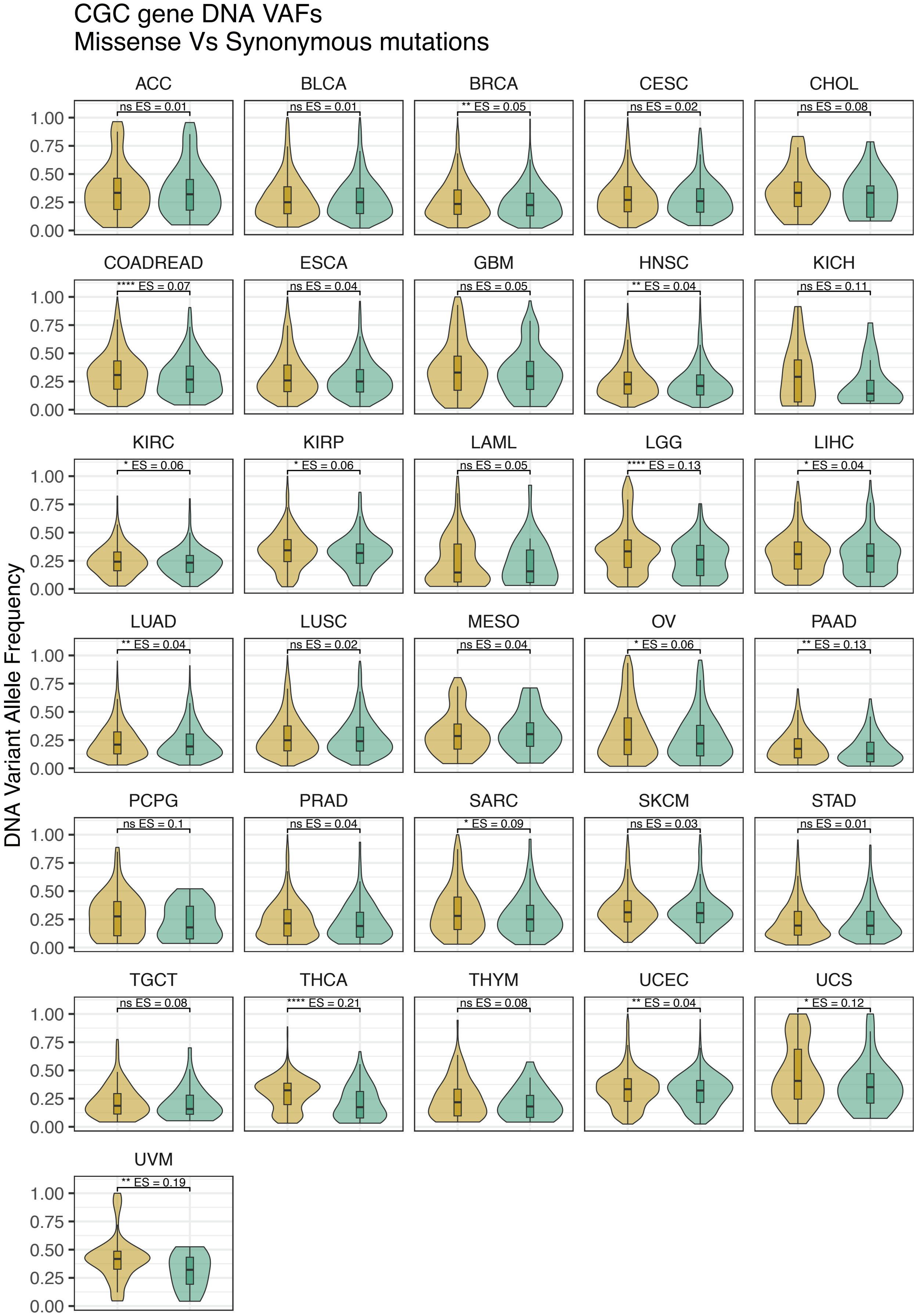

*Figure S7: Comparison of DNA VAFs between missense and synonymous mutations in CGC genes across individual cancer types in the TCGA cohort; ns indicates p > 0.05, * indicates p < 0.05, ** indicates p < 0.01, *** indicates p < 0.001, **** indicates p < 0.0001; Wilcoxon test. ES: effect size*

Figure S8

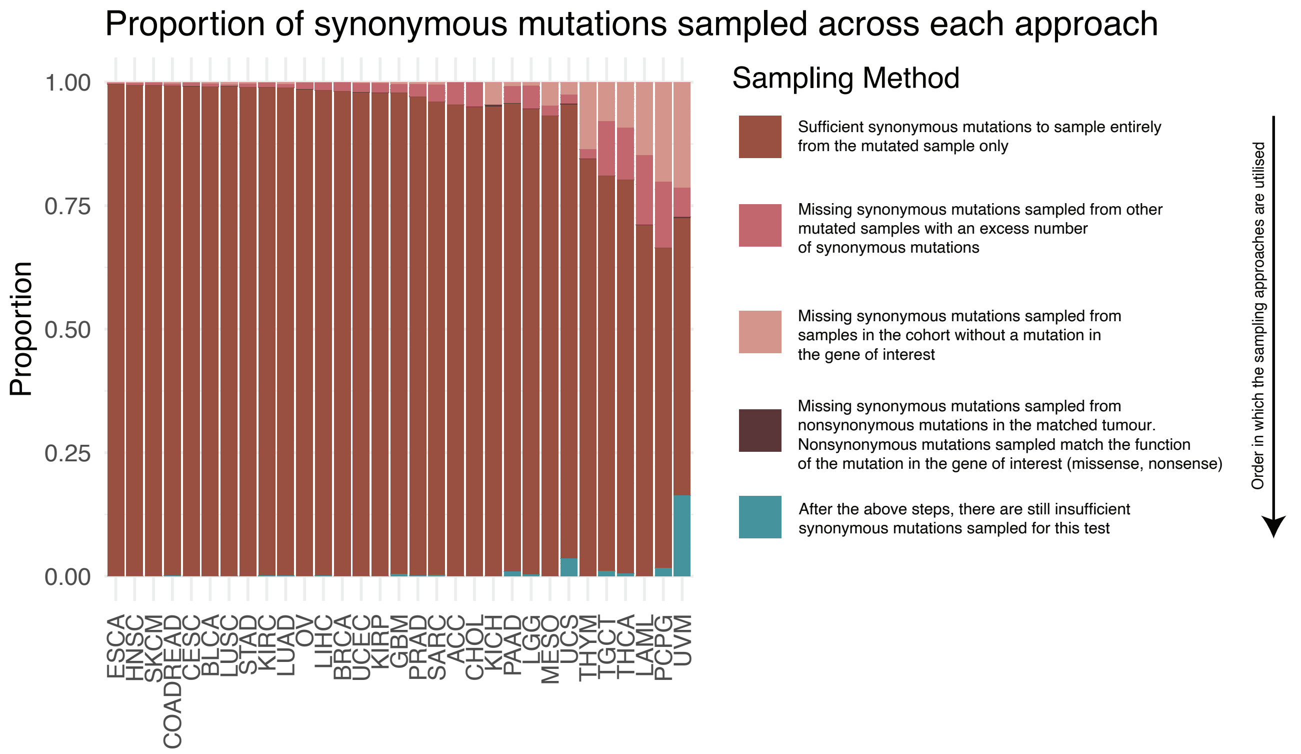

Figure S8: A summary of the average proportion of synonymous mutations sampled from i) within the mutated sample; ii) within other mutated samples; iii) from the global synonymous mutations; iv) from the same mutation type, within the mutated sample. This hierarchical stepwise approach is described in detail within the methods.

Figure S9

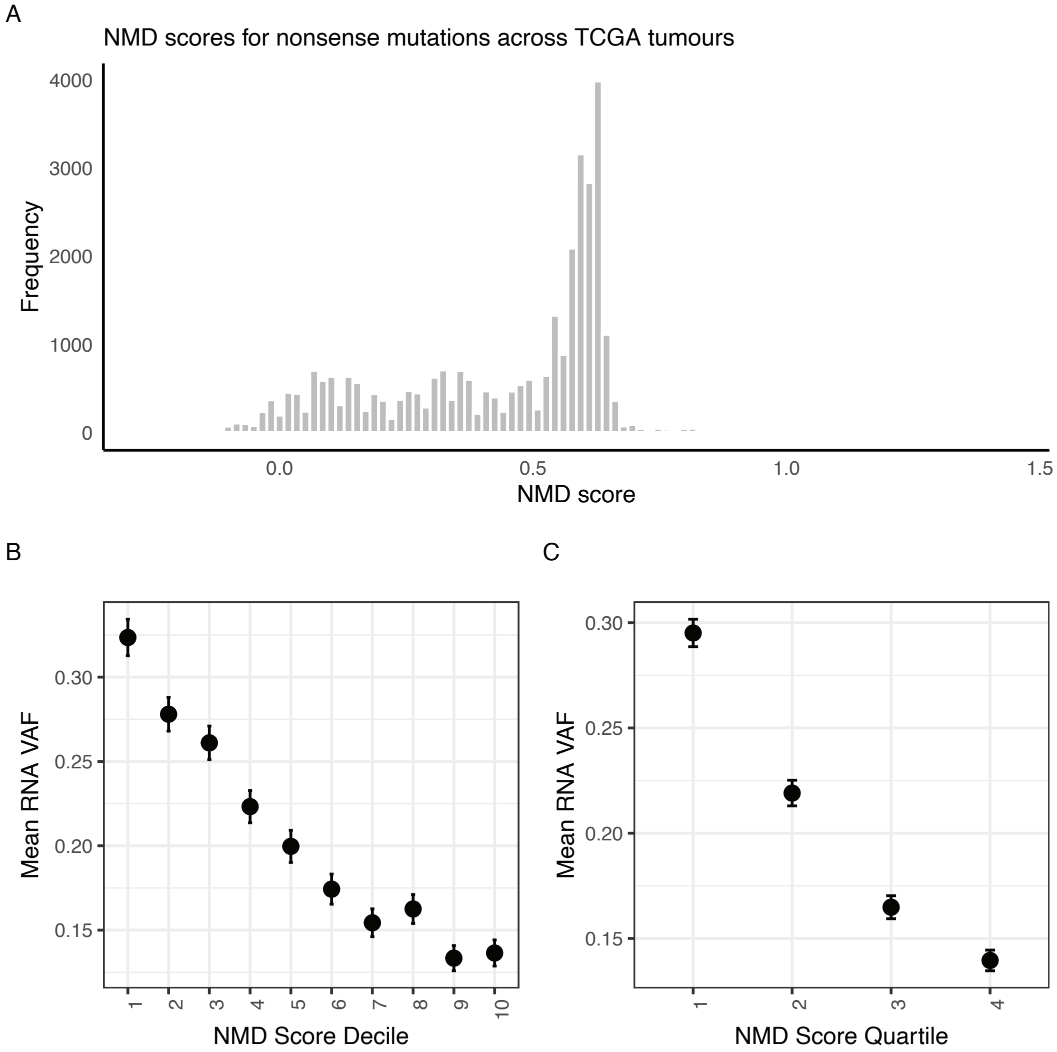

Figure S9: RNA VAF among mutations subject to nonsense-mediated decay (NMD). A shows the distribution of NMD scores among nonsense mutations across all cancer types in TCGA. NMD scores were derived from Lindeboom et al. B shows RNA VAFs of mutations within each NMD score decile. The mean and 95% confidence intervals of RNA VAFs of mutations within each decile are displayed. These results were then used for scaling of nonsense mutations. C. NMD scores were binned into quartiles instead of deciles.

Figure S10

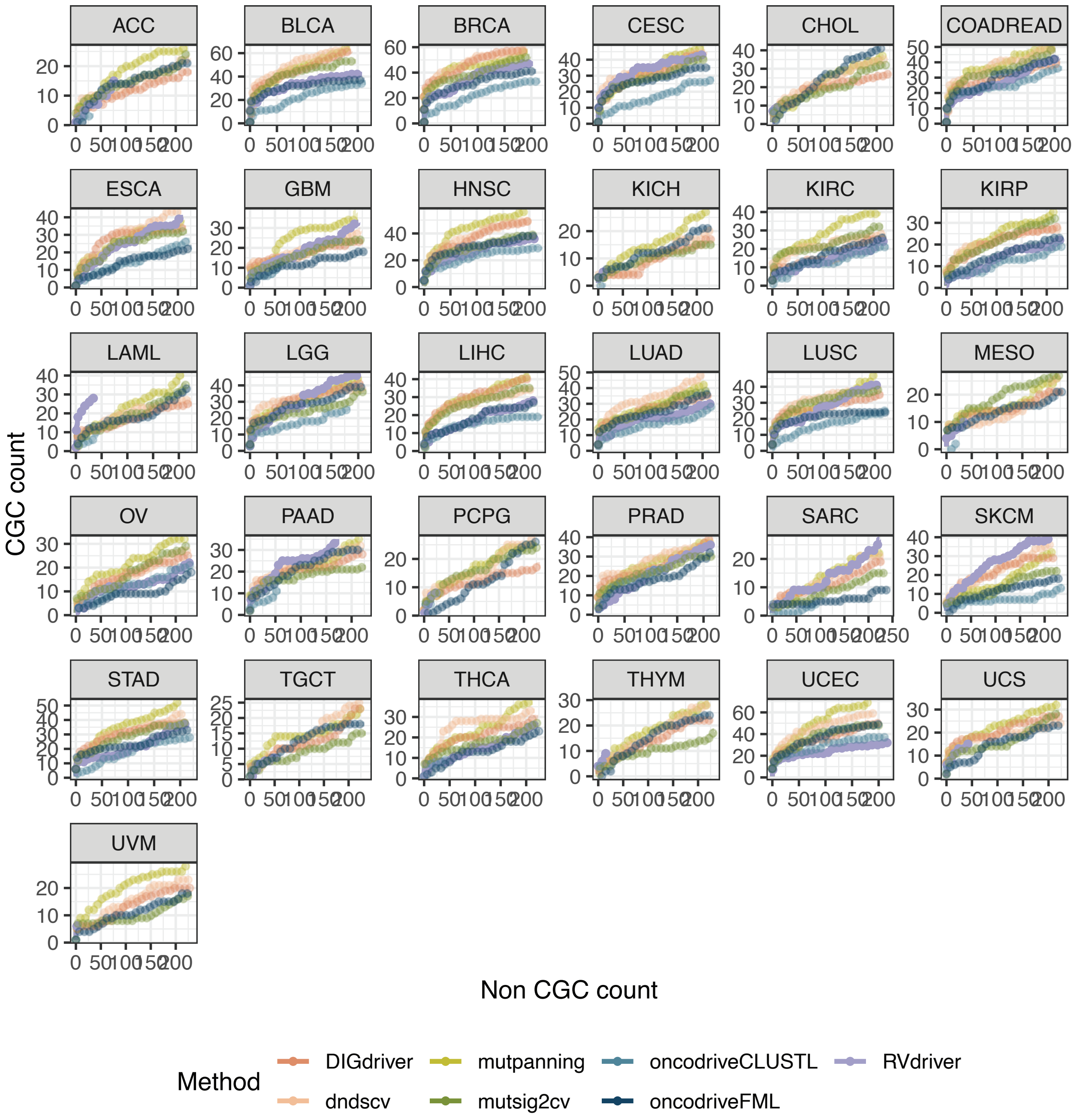

Figure S10: Benchmarking of RVdriver across all cancer types against six other established tools leveraging DNA information, DIGdriver, dNdScv, mutpanning, MutSig2cv, oncodriveclustl, and oncodriverfml. Genes within the COSMIC cancer gene census list were treated as true positive results, and other genes as true negative results. The figure displays the number of CGC genes (y-axis), versus non-CGC genes (x-axis), identified within the top hits by the tools until 250 non-CGC genes were reached.

Figure S11

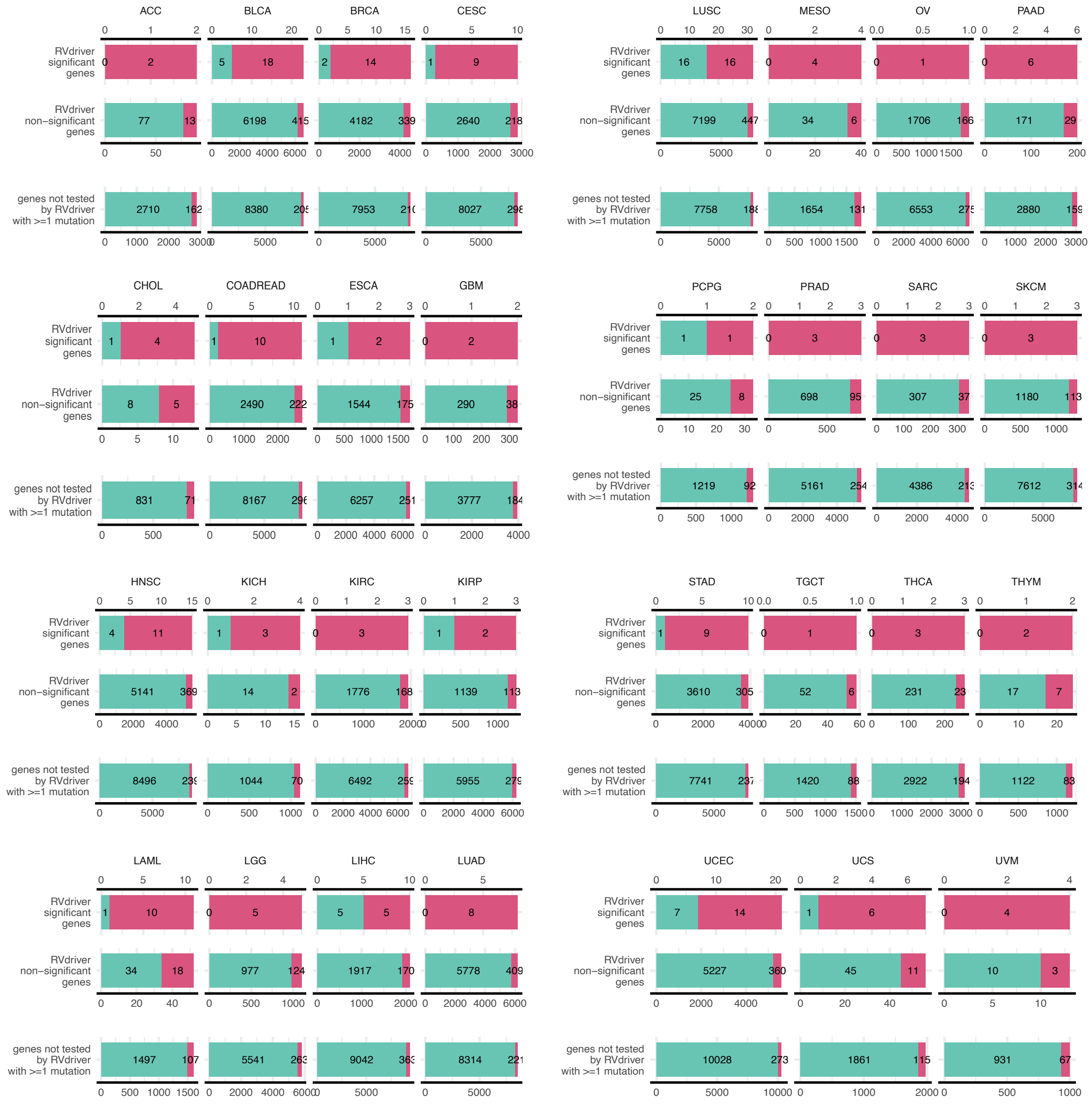

Figure S11: The number of genes assigned as putative cancer genes by RVdriver (top); the number of genes tested by RVdriver that were not assigned as putative cancer genes (middle); the number of genes with >= 1 mutation that were not tested by RVdriver (bottom). Each panel is split into CGC and non-CGC genes

Figure S12

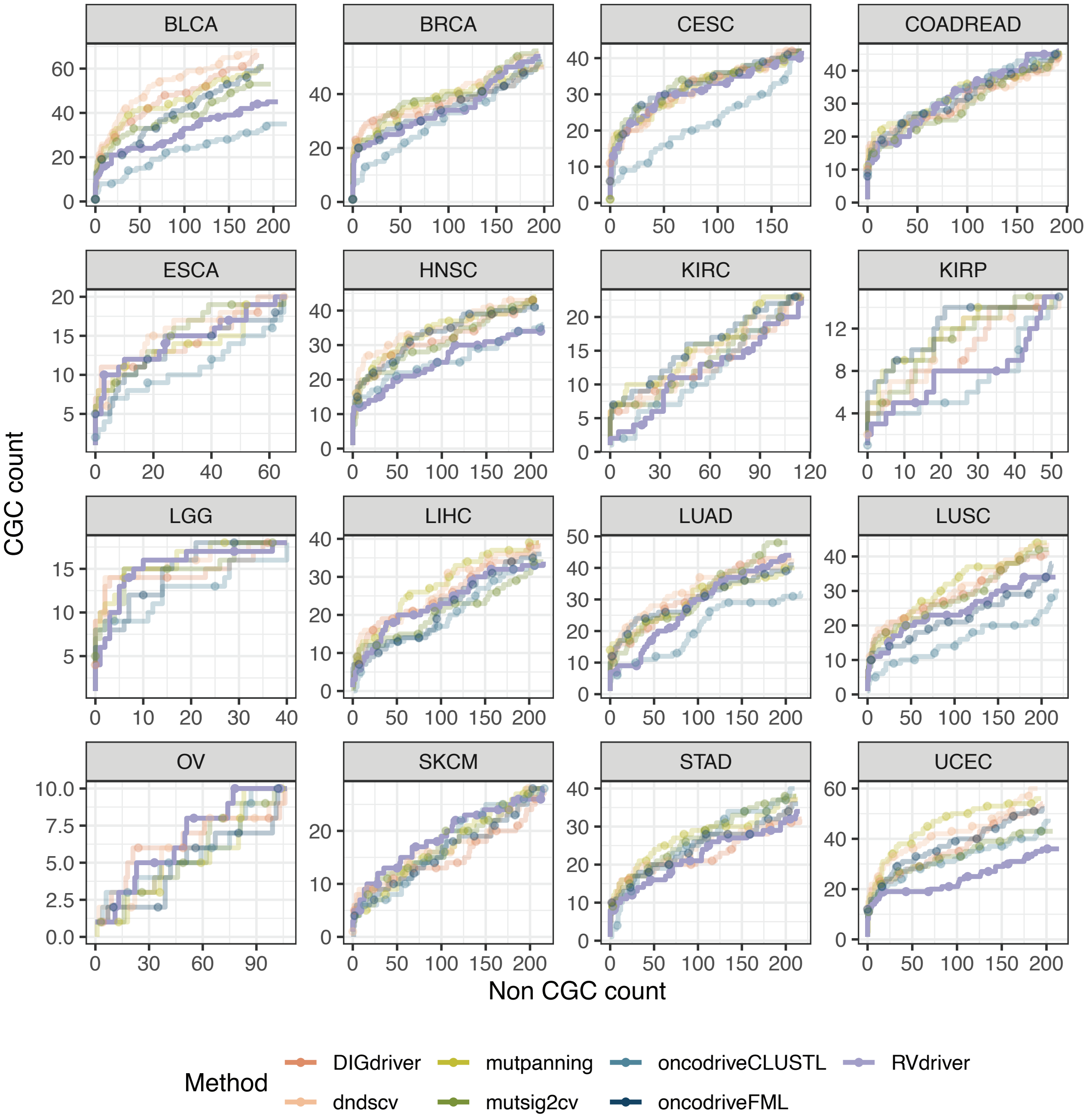

Figure S12: Benchmarking of RVdriver using a unified geneset of genes tested by all six other established tools leveraging DNA information, DIGdriver, dNdScv, mutpanning, MutSig2cv, oncodriveclustl, and oncodriverfml. 16 cancer types are shown that had a unified geneset > 50 genes tested. Genes within the COSMIC cancer gene census list were treated as true positive results, and other genes as true negative results. The figure displays the number of CGC genes (y-axis), versus non-CGC genes (x-axis), identified within the top hits by the tools until a maximum of 250 non-CGC genes were reached.

Figure S13

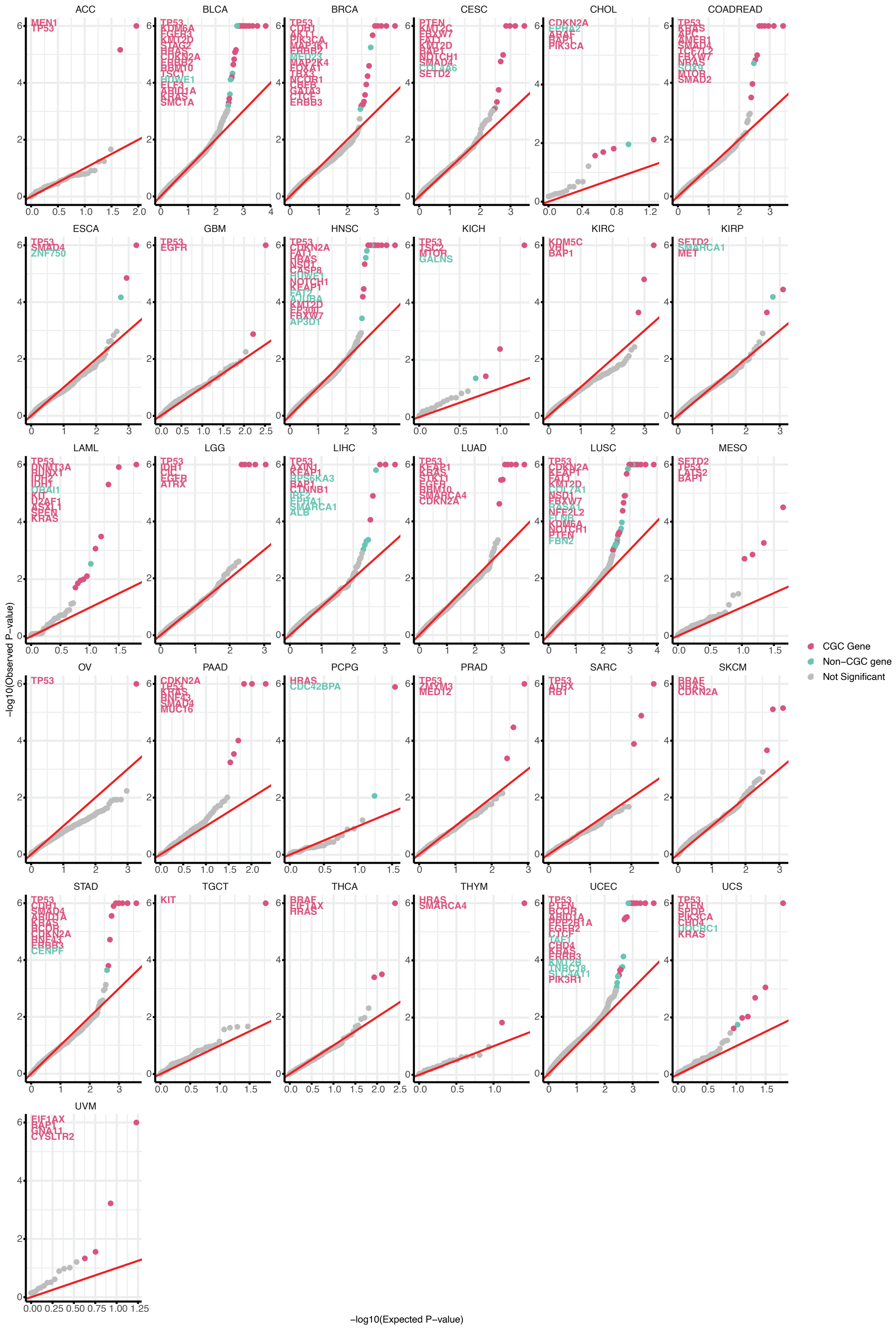

Figure S13. QQ plots for RVdriver. Significant genes (q value < 0.25) are highlighted in red (CGC gene) and green (Non-CGC gene). Gene names are displayed in descending order of statistical significance until the 15th most significant gene. The p-value for each gene was derived by taking, in cases with multiple mutation functions tested separately, the most significant test per gene.

Figure S14

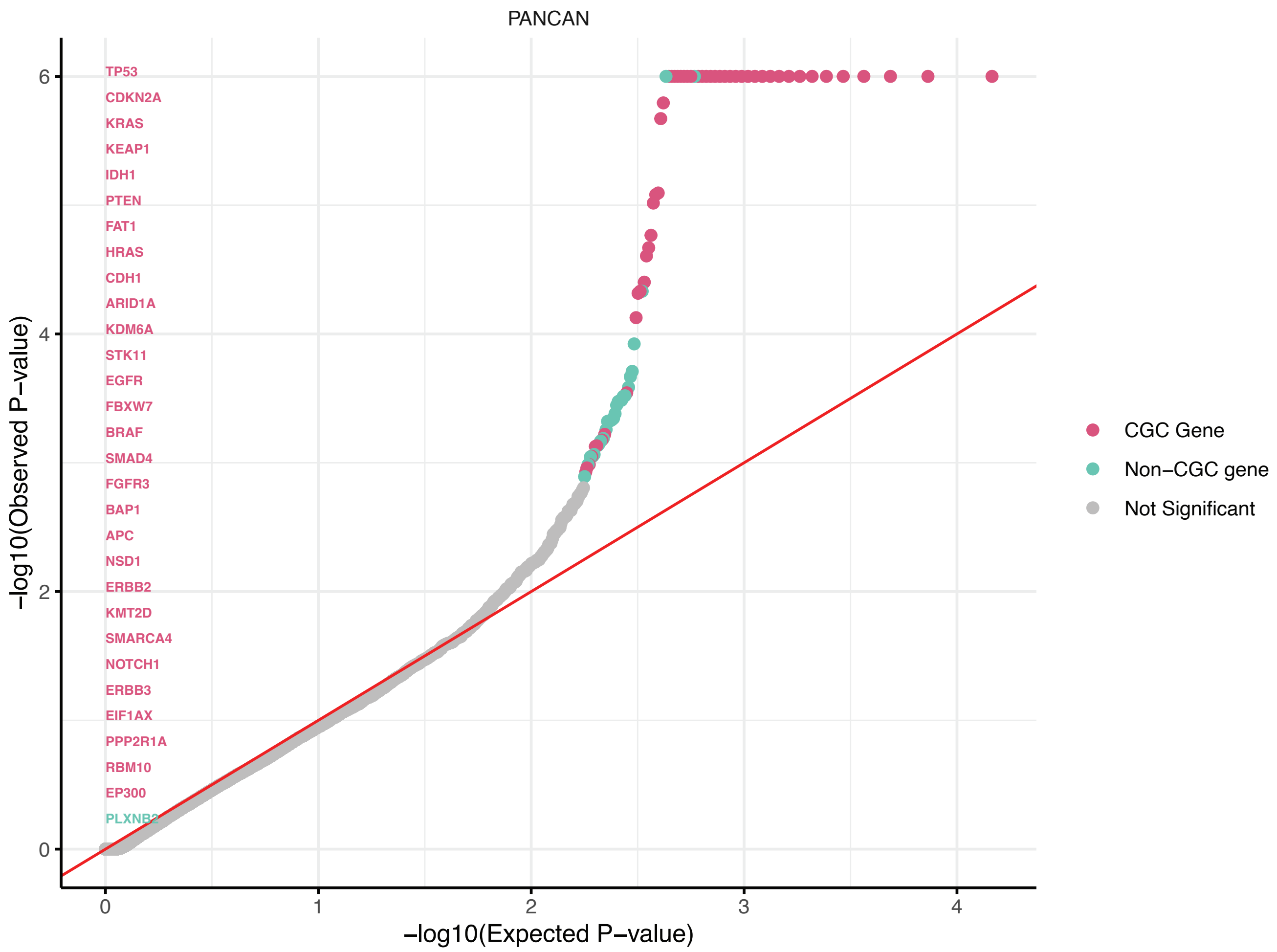

Figure S14. QQ plot for RVdriver, run on the pan cancer TCGA cohort described in this manuscript of 7,882 tumours. Significant genes (q value < 0.25) are highlighted in red (CGC gene) and green (Non-CGC gene). Gene names are displayed in descending order of statistical significance until the 30th most significant gene. P-values for each gene were derived from the test for all nonsynonymous mutations present within the gene of interest.

Figure S15

A

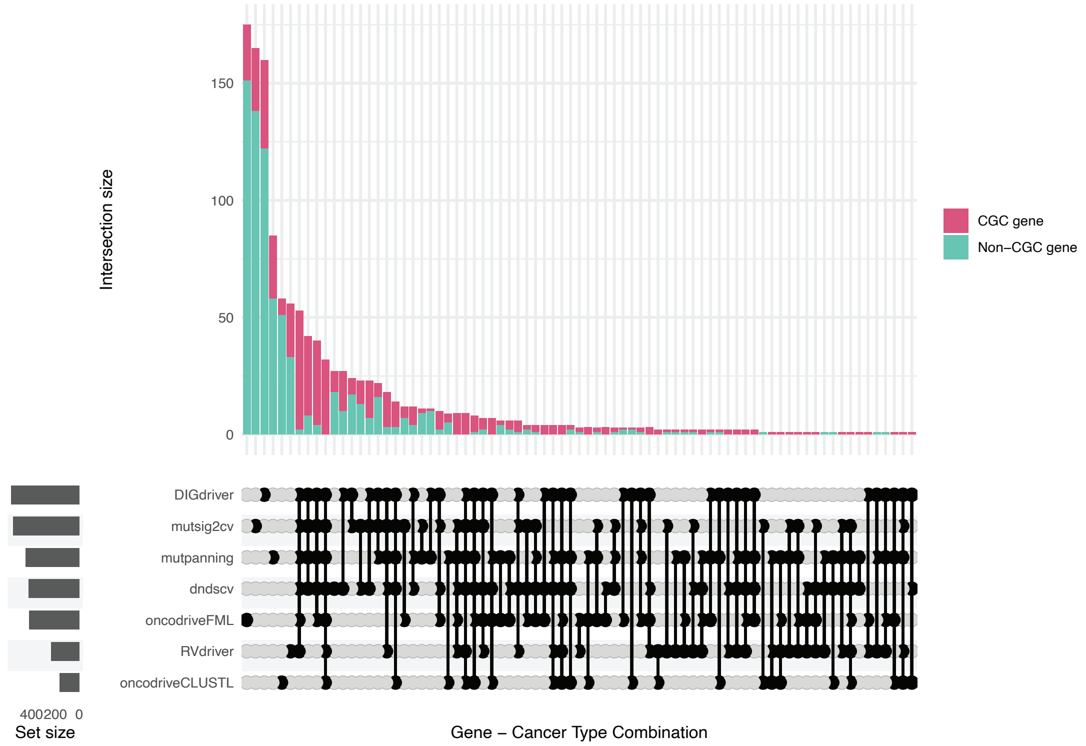

B

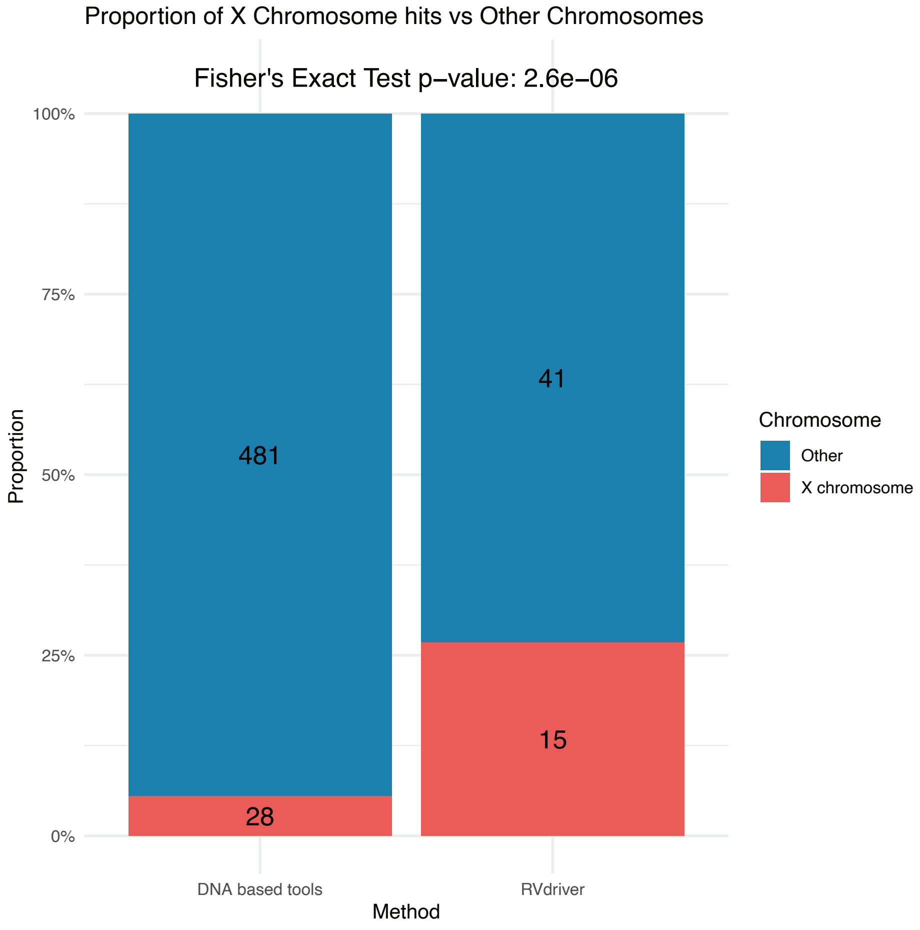

Figure S15: A. UpSet plot showing overlap between the CGC and non-CGC genes discovered by RVdriver and the six DNA based approaches. B. Enrichment for genes on the X chromosome for putative cancer gene, cancer type combinations uniquely identified by RVdriver compared to 5 DNA based tools (DIGdriver only tests mutations lying on autosomes so was not included in this analysis).

Figure S16

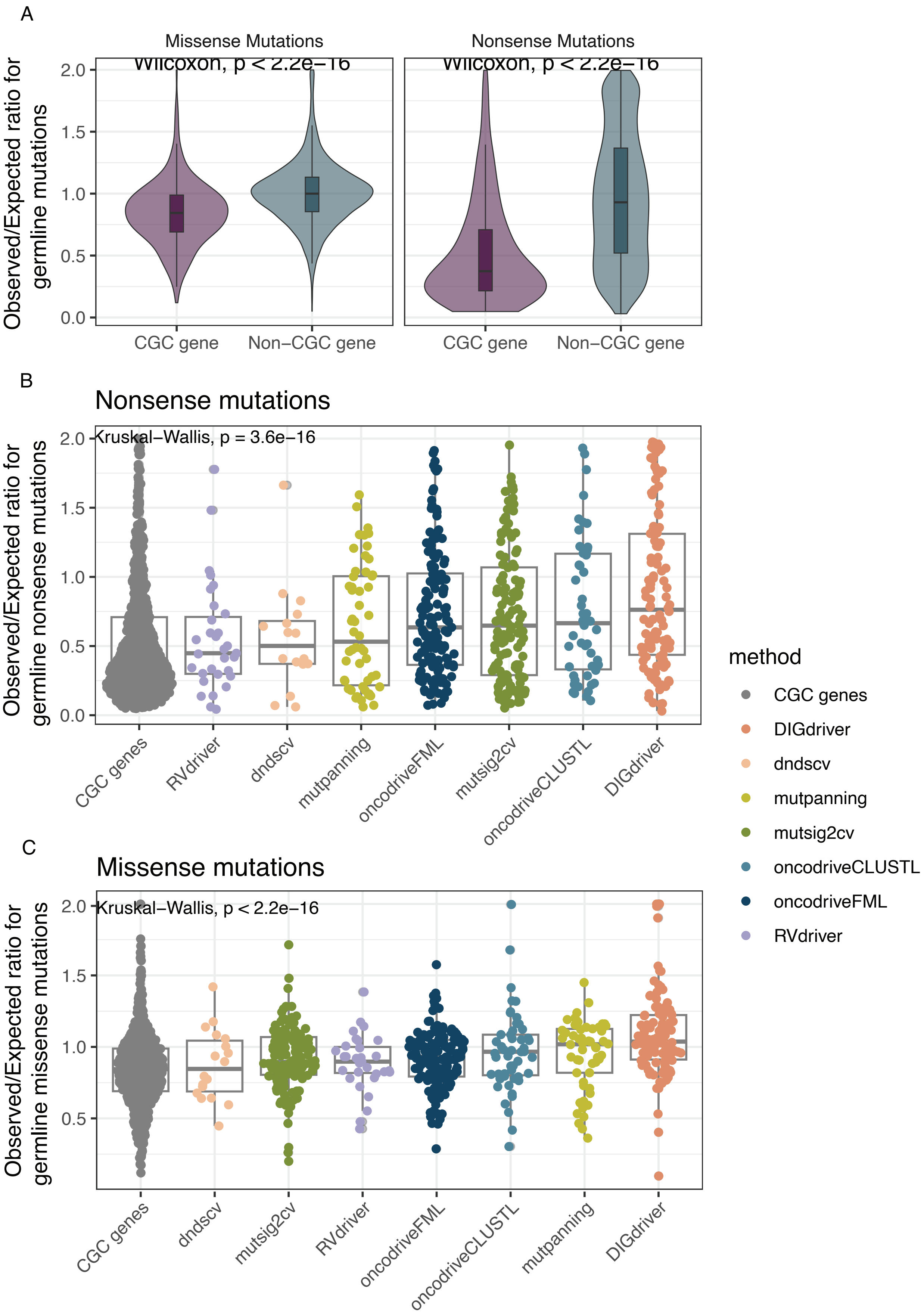

Figure S16: A. Essentiality scores for genes in the COSMIC cancer gene census. Essentiality scores were derived from the Observed-Expected upper bound fraction for germline missense (left) and nonsense (right) mutations. B,C: Comparison of the distributions of essentiality scores for putative cancer genes identified uniquely by each tool. Essentiality scores were derived from the Observed-Expected upper bound fraction for germline nonsense (B) and missense (C) mutations. Essentiality scores for CGC genes are also shown. Boxplots are ordered by ascending median of the observed/expected upper bound fraction of germline mutations.

Figure S17

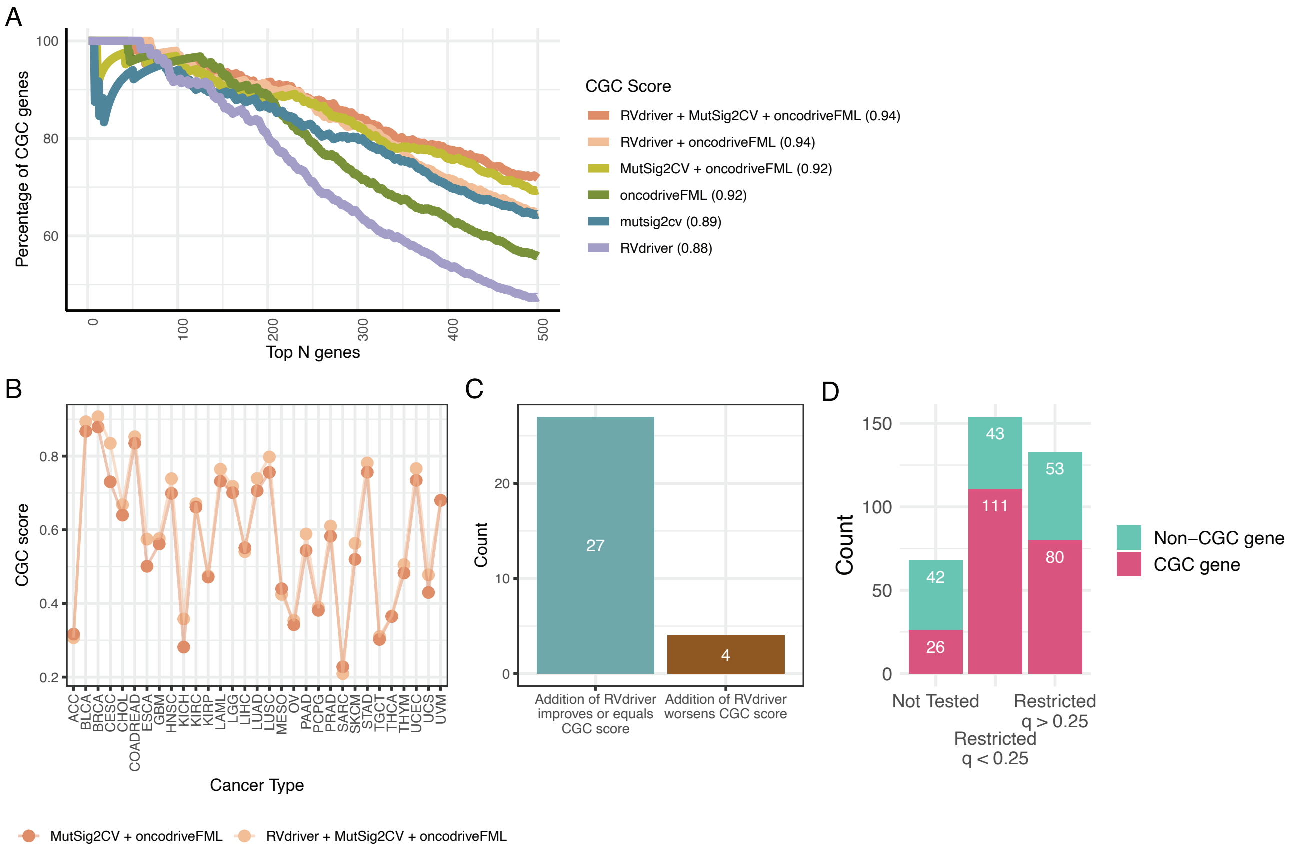

Figure S17. A. Pan-cancer enrichment for CGC genes, (CGC score) among selected tools, and combinations of tools. The CGC score is a ranking score for the proportion of CGC genes within the top 250 genes (see Methods). B. CGC score within each cancer type. CGC score calculated at n=40, unless less than 40 genes were tested within the cancer type. Dots correspond to CGC score from the combination of MutSig2CV and OncodriveFML; and the addition of RVdriver to those approaches. C. Cancer types among which the addition of RVdriver to MutSig2CV and OncodriveFML equals or improves the CGC score, compared to those in which it worsens the score. D. Results from putative cancer genes identified by at least two of DIGdriver, dNdScv, mutpanning, MutSig2cv, oncodriveclustl, and oncodriverfml, and not by RVdriver. Restricted hypothesis testing was performed on RVdriver results among these genes. 154 of 287 such genes were significant with restricted hypothesis testing.
