# Supplementary material for: Cancer gene identification from RNA variant allelic frequencies using RVdriver
Source: Genome Biol. 2025 Jun 13;26:165. doi: 10.1186/s13059-025-03557-y (PMC12164115; doi:10.1186/s13059-025-03557-y)
Supplement: Supplementary file 4 — Additional file 4. Review history [file 13059_2025_3557_MOESM4_ESM.docx]

Review History

**First round of review**

**Reviewer 1**

**Were you able to assess all statistics in the manuscript, including the appropriateness of statistical tests used?**

Yes, and I have assessed the statistics in my report.

**Comments to author:**

This article proposes a computational tool, RVdriver, in order to identify putative cancer genes by analyzing RNA variant allele frequencies from bulk genomic-transcriptomic data across multiple cancer types. Unlike most of existing tools for drive gene annotation that are DNA-based approaches, RVdriver leverages RNAseq data, which provides information about gene expression levels and alternative splicing events, to identify functional driver mutations that may be missed by DNA-based approaches. The authors applied RVdriver to frequently mutated genes in TCGA across 7,948 paired exomes and transcriptomes from 30 cancer types. The reviewer has the following major concerns.

1. Introduction: "approaches that incorporate transcriptomic information as a bridge between the frequentist approaches to cancer gene discovery that focus exclusively on the genome, and tumor phenotype, are lacking". This statement presents a logic that is faulty in the reviewer's opinion. There are a huge literature when it comes to incorporating transcriptomic information to annotate genetic mutation, as in eQTL, allele-specific expression, etc. On the other hand, does a formal definition of cancer gene exist? For example, DNA-based approaches are developed to identify cancer gene/driver mutations because they start with an assumption that cancer genes are those that are frequently mutated and those are driver mutations would lead to changes in tumor outcomes. With such assumption, DNA-based approaches find genes that are potentially interesting. Now is this paper introducing a new definition of cancer gene, so as to bring in the transcriptomic activity as a property? If so, then shouldn't the methods in literature on connecting transcriptome and genetic mutations all be evaluated here? What those studies didn't do or say, which is claimed to be lacking here, was re-defining cancer gene, which is more conceptual than what the tools actually do. So what exactly is new here? Of course, if one expands the definition of cancer gene, one could show some new findings. But those findings need to be further validated.
Overall, the reviewer thinks the premise of the entire paper is a moving target, which is a major weakness.

2. Other than mutation-level data, the total # of Cosmic Cancer Gene Census (CGC) genes and the total # of non-CGC genes are never shown. This prevents a proper evaluation of false positive and false negative rates across the paper. Along this line, none of the reported new putative cancer genes, as compared to existing methods, are independently validated to be cancer genes (based on the old+new definition, or what definition?). For example, no survival outcomes, which are available in TCGA, have been evaluated. Shouldn't driver mutations in cancer genes lead to some tractable biological/clinical outcome differences?

3. Figure 1F is misleading as the sidebar represents 1 standard deviation, so the total length of the bar here corresponds to only ~70%CI, as compared to the usual 95%CI for measure of uncertainty. This means the variation of RNA VAF is much higher than what is shown in 1F. With that in mind, the reviewer is not convinced this conclusion: "clonal mutations, mutations in allelic imbalance and mutations showing allele-specific expression exhibited elevated RNA VAF (Figure 1F). Furthermore with Figure 1H, even though the difference in RNA VAF between missense and synonymous mutations within CGC genes is statistically significant, there seem to a similarly wide range of RNA VAF for each category. This plot actually argues against defining only mutations that present statistically significantly higher/lower RNA VAF than the background (synonymous mutations) as driver mutations, as there are also a lot of mutations that would be missed that way. To the reviewer, this plot only suggests there are some mutations in cancer genes that will present strong signals in RNA VAF. This does not necessarily lead to the next step, to use RNA VAF to annotate functional vs. nonfunctional mutations.

4. Since RVdriver requires a minimum of 4 mutations within a given gene across all tumors and a q-value <0.01 as strong enough evidence, the reviewer would like to see in the TCGA data, in each cancer type, how many genes actually had enough data to run RVdriver and how many of them came out as non-cancer genes, i.e., q-value > 0.05. This data is not currently provided in the manuscript.

5. "Across the 30 cancer types, it identified 136 instances of CGC genes." This number seems very low. How many "instances of CGC genes" are there in total as ground truth? Are we talking about a sensitivity of <10%?

6. "Compared to 50 non-CGC genes (identified by RVdriver), highlight its ability to identify meaningful biological patterns". The reviewer does not follow the logic here. RVdriver has not been independently validated so what it finds may not be true.

7. The reviewer has a lot of problems with Figure 2. 2A, it is really the insert small panel that is more meaningful as it shows the beginning of the curve, where the inclusion of non-CGC genes (as false positives) is less than 100. Across cancer types, RNA VAF does not do as well as dndscv (no.1) or mutpanning (no.2). 2B, all ROC curves look odd, as if very few data points were used. This can be proven by showing the actual number of genes (should be very small) and attach 95%CIs (should be very wide, hence really no power to compare across methods). 2C, not clear how these ROC curves are calculated and why are they comparable since the n (not sure what this sample size is about) listed is quite different across methods. 2D, here non-CGC genes are switched from serving as true negatives, to serving as an unknown pool for discovery. If following such logic, then oncodrivefml and mutpanning find a lot more putative cancer genes than RNA VAF. Overall, It is odd to have the same data serving both logics. Analysis using independent truth data should be performed.

8. Figure 3 shows dndscv is doing consistently well, finding more CGC genes than RNA VAF. The reviewer counted the total number of cancer genes annotated by RNA VAF vs. dndscv and found that across 30 cancer types, dndscv found the same or more cancer genes than RNA VAF in 23 cancer types. It seems RNA VAF could further annotate more CGC genes that were missed by dndscv but by itself it does not do as well, hence not an independent tool to be used.

9. A similar logic loop as above was applied to studying driver vs. passenger mutations as compared to BoostDM, hence the results are not convincing.

**Reviewer 2**

**Were you able to assess all statistics in the manuscript, including the appropriateness of statistical tests used?**

Yes, and I have assessed the statistics in my report.

**Comments to author:**

Summary. The "RVdriver" algorithm employs RNA variant allele frequency (VAF) as a signal for positive selection to identify and classify mutations in cancer-associated genes. Black et al. first provide motivation for focusing upon instances of elevated RNA VAF. The authors proceed to validate, among canonical cancer and non-cancer genes, higher RNA VAFs in cancer versus non-cancer genes, and differences between RNA VAF from non-synonymous and synonymous mutations. A mathematical description of RVdriver is then presented. RVdriver is based upon fitting RNA VAF from nonsynonymous SNVs from a filtered MAF file with a linear mixed effects model. A mutation's function is given fixed effect size, and patient ID is given random effect size, for hierarchical dependence between RNA VAF and factors such as tumor purity and expression level. Each linear mixed model is fitted to 50 bootstrap samples of synonymous mutations, a t-value is calculated for mutation function effect size, and the median t-value is used to compute a p-value (from Student's t-distribution). After multiple test correction, a cancer-associated mutation is classified with high-confidence (q < 0.01) or low-confidence (0.01 < q < 0.05). RVdriver's performance is then evaluated (i) with AUC-ROC for cancer gene detection versus a panel of four DNA-based algorithms, and (ii) in terms of pan-cancer driver/passenger classification sensitivity/specificity versus BoostDM in silico saturation mutagenesis predictions (Muiños et al., 2020). RVdriver identified more COSMIC Cancer Gene Census (CGC) genes than non-CGC genes and produced comparable AUC versus the selected algorithms. Several gene-cancer type combinations recurred between RVdriver and DNA-based methods, and several novel combinations were also identified. Driver/passenger classification performance versus BoostDM revealed the following: (1) a strong correlation between driver/passenger annotation and RNA VAF among missense mutations in tumor suppressors; (2) notable disagreements between RNA-VAF and BoostDM (e.g., EGFR, PIK3CA); and (3) higher overall performance for driver gene identification among tumor suppressors than oncogenes.

Summary of Assessment. RVdriver offers several important contributions yet has several critical limitations. Discrepancies between RVdriver and Intogen's BoostDM for driver/passenger classification uncover closer correspondence between RNA VAF-based classification and tumor phenotype than for BoostDM-based classification. Greater VAF differences between nonsynonymous and synonymous mutations from RNA versus DNA suggest that transcriptomics provides additional information regarding mutation classification and phenotype. Black et al. also note the discovery of unique cancer-associated genes/phenotypes with RVdriver; e.g., reduced EGFR signaling in glioblastoma.
RVdriver also has several impediments to publication in Genome Biology. First, higher performance has been observed from deep learning-based algorithms. It would enhance the manuscript to compare to the recent DIG method/tool (Sherman, Yaari et al., Nature Biotech, 2022), since it has also been applied to coding regions, as focused on in the current manuscript. DIG offers performance and flexibility advantages versus the analyzed method panel (e.g., dNdScv), enabling genome-wide mutation rate mapping and driver gene discovery for intergenic or noncoding elements. Use of convolutional neural networks in tandem with a Gaussian Process also allows for uncertainty quantification.
RVdriver may further benefit from more explicit modeling of a mutation's functional impact. Methods such as DriverPower incorporate both mutational burden and functional impact (averaged metrics from established methods), which can reweight burden-based driver gene identification (Shuai et al., Nat Comm, 2020). The RVdriver method may also benefit from combining genomic, transcriptomic, and functional impact data, as well as comparison of performance upon use of each data type alone. As the authors note, RVdriver may generate false positive results during cancer gene identification / driver mutation discovery. Lawrence et al., Nature (2013) note that assuming a constant background mutation rate generates numerous false positive findings due to somatic mutational heterogeneity. It would seem that RVdriver's aggregation of RNA VAF from bootstrap sampling of synonymous mutations assumes constant background RNA VAF and thus does not consider mutational heterogeneity or elevated false positive rate. Moreover, MutSigCV includes pooling of mutation counts and seq. coverage between genes that are proximal in high-dimensional covariate space. RVdriver's aggregation of sampled synonymous RNA VAFs does not incorporate such consideration.

Major Comments:

RVdriver provides several important biological insights:

* Greater discrepancies between the VAF of nonsynonymous vs. synonymous mutations using RNA VAF, rather than DNA VAF.
* Closer phenotypic correspondence for RNA VAF than DNA VAF.
* Discovery of novel mutation-tumor type combinations in CGC and non-CGC genes.
* Discovery of EGFR signaling phenotypes in glioblastoma.

Important concerns regarding the RVdriver method include:

* Deep learning-based cancer gene discovery methods can attain higher performance (measured with AUC-ROC), allow for genome-wide discovery, and quantify predictive uncertainty. They should be compared with.
* More explicit modeling of functional impact, along with genomic and transcriptomic data, may improve cancer gene discovery / classification.
* Bootstrap sampling and aggregation of RNA VAF from 10 selected synonymous mutations can produce false positive findings. Previous studies have shown that false positive findings arise from assumption of constant background mutation rate in the presence of mutational heterogeneity.
* RVdriver could also benefit from aggregation of data from sampled synonymous mutations with similar values for covariates of background mutation rate (e.g., replication timing or expression level).

Minor Comments:

* Calculation of pan-cancer statistics for driver/passenger classification relative to the Cancer Gene Census, as well as relative to BoostDM, may be helpful for more clearly interpreting performance.
* The Discussion might benefit from an additional portion that considers avenues for recovering cancer genes that were not resolved by RVdriver, e.g. PIK3CA.
* The authors note higher performance (AUC-ROC) for missense mutations in tumor suppressor genes than in oncogenes. The implications of using RVdriver alone or with other methods could be considered in the context of such performance differences.

**Authors Response**

**Point-by-point responses to the reviewers’ comments:**

**Rebuttal for manuscript ‘RNA allelic frequencies encode substantial functional variation in cancers’**

We thank the Reviewers for their detailed comments. We were very pleased that our manuscript was viewed as offering an important contribution to the field.

The Reviewers both highlighted a lack of thorough validation of the putative novel cancer genes identified by RVdriver. In addition, our use of AUC curves to assess the performance of RVdriver was criticised.

Reviewer 2 was positive about the scope and aims of the work. We feel that some of the comments made by Reviewer 1 reflect a lack of clarity within the text and a failure on our part to crystallise succinctly the aspects of the original manuscript that we felt to be the most novel. We have therefore made an effort to correct the basis for what we feel are several misunderstandings about the method and aims of the manuscript in order to address these concerns.

In addition to these changes to the text of the paper, which are detailed in our point-by-point response, we have made the following extensive changes to the manuscript:

1. Alteration of RVdriver model and methodology to improve performance and reliability

In response to a suggestion from Reviewer 2, we investigated ways we could adjust for differential RNA VAFs of background mutations. We have made extensive changes to the method which have improved performance relative to the previous version of the manuscript. We have altered our background model of mutation RNA VAF, and now implement a linear model, rather than a linear mixed effects model; and leverage a multi-step sampling approach of background mutations including a scaling approach to control for mutation type, an adjustment to control for the impact of nonsense mediated decay and a weighting that adjusts for the RNA coverage. This has enhanced performance relative to the version of RVdriver that was presented previously.

2. Addition of multiple layers of validation to provide reassurance of added value provided by RVdriver

It can be difficult to validate putative novel cancer genes, given that they by definition have not hitherto been described as such, but must surely exist. Nonetheless, we have added multiple steps of validation to provide assurance that the novel hits we describe might indeed be cancer genes. These include an assessment of the degree of disruption to these genes in the germline (with functionally impactful genes typically tolerating less disruption), a literature search to evaluate whether other publications have suggested a role for the putative cancer genes in cancer, and an addition of a validation cohort in lung adenocarcinoma and lung squamous cell carcinoma to underscore that our method is capable of describing consistent hits across cohorts.

3. Addition of 2 further tools (DIGdriver and MutSig2cv) for comparison and benchmarking of RVdriver output

In response to comments from Reviewer 2, we have implemented 2 additional tools to assess the relative performance of RVdriver, and we have explicitly evaluated the relative impact of tools that assess mutation functional impact above and beyond that of frequentist tools, and where RVdriver is capable of adding to performance.

4. Changes to approach to evaluating performance of RVdriver and other tools

Our use of Area Under the ROC curve plots, with sensitivity and specificity for discovery of Cancer Gene Census genes as the basis for assessing performance, was criticised. As such, we have changed our approach to now rank the gene cancer type combinations by their significance and plot the percentage of CGC genes across the top 500 genes for each method. We also compute a ‘CGC score’, utilised in the intogen pipeline to evaluate the performance of each tool. The CGC score is computed using the following equation:

$$E(R)=\Sigma_{i=1}^{N} \frac{Pi}{log(i+1)}$$

Where $Pi$ is the proportion of genes with greater significance than $i$which are within the CGC list. N is a user defined threshold to consider only ‘top ranked’ genes. For our pan-cancer plot, we computed the CGC score using N=250. In doing so, we evaluate and compare the performance of RVdriver to other DNA based methods in a more clear and meaningful way.

**Point by point rebuttal:**

Reviewer reports:

Reviewer #1: This article proposes a computational tool, RVdriver, in order to identify putative cancer genes by analyzing RNA variant allele frequencies from bulk genomic-transcriptomic data across multiple cancer types. Unlike most of existing tools for drive gene annotation that are DNA-based approaches, RVdriver leverages RNAseq data, which provides information about gene expression levels and alternative splicing events, to identify functional driver mutations that may be missed by DNA-based approaches. The authors applied RVdriver to frequently mutated genes in TCGA across 7,948 paired exomes and transcriptomes from 30 cancer types. The reviewer has the following major concerns.

1. Introduction: "approaches that incorporate transcriptomic information as a bridge between the frequentist approaches to cancer gene discovery that focus exclusively on the genome, and tumor phenotype, are lacking". This statement presents a logic that is faulty in the reviewer's opinion. There are a huge literature when it comes to incorporating transcriptomic information to annotate genetic mutation, as in eQTL, allele-specific expression, etc. On the other hand, does a formal definition of cancer gene exist? For example, DNA-based approaches are developed to identify cancer gene/driver mutations because they start with an assumption that cancer genes are those that are frequently mutated and those are driver mutations would lead to changes in tumor outcomes. With such assumption, DNA-based approaches find genes that are potentially interesting. Now is this paper introducing a new definition of cancer gene, so as to bring in the transcriptomic activity as a property? If so, then shouldn't the methods in literature on connecting transcriptome and genetic mutations all be evaluated here? What those studies didn't do or say, which is claimed to be lacking here, was re-defining cancer gene, which is more conceptual than what the tools actually do. So what exactly is new here? Of course, if one expands the definition of cancer gene, one could show some new findings. But those findings need to be further validated.

Overall, the reviewer thinks the premise of the entire paper is a moving target, which is a major weakness.

We thank the reviewer for highlighting confusing and potentially misleading aspects in original submission. Below we address the key issues and how we have attempted to address them.

1. “There are a huge literature when it comes to incorporating transcriptomic information to annotate genetic mutation, as in eQTL, allele-specific expression, etc.”

This manuscript specifically deals with incorporating transcriptomic signals into assessments of whether a gene is a cancer gene or not. Importantly, this is different from, for example, an eQTL analysis in its scope, and to our knowledge, is the first such manuscript to do this. To the best of our knowledge, having searched the literature, there are no other manuscripts that leverage eQTL, ASE, or other transcriptomic analysis to identify cancer genes. We hope our revised manuscript has successfully conveyed this message.

2. “...does a formal definition of cancer gene exist? For example, DNA-based approaches are developed to identify cancer gene/driver mutations because they start with an assumption that cancer genes are those that are frequently mutated and those are driver mutations would lead to changes in tumor outcomes.”

We apologise for confusion regarding our definition and representation of cancer genes. Critically, we do not seek to in any way redefine or expand the scope of what a cancer gene is. A cancer gene is a gene that is causally implicated in cancer; a driver mutation reflects a mutation that confers a fitness advantage to the cancer cell within a given environment. Given how essential understanding this is to appreciating the aims and scope of the paper, we have rewritten the relevant sections of the introduction. We apologise for our previous lack of clarity. Please see below – we hope this addresses this Reviewer’s concern.

- “As such, multiple algorithms and computational tools have been developed to identify patterns of excessive mutagenesis and thus ‘cancer genes’, genes which are causally implicated in cancer.”
- “However, approaches that incorporate transcriptomic information as a window into tumour phenotype through which cancer genes can be identified are lacking.”

2. Other than mutation-level data, the total # of Cosmic Cancer Gene Census (CGC) genes and the total # of non-CGC genes are never shown.

Thank you for highlighting this – we agree it would be useful to include this information. We have added information about the number of CGC and non-CGC genes tested per cancer type by RVdriver to the plots in Figure 2B as shown below.

This prevents a proper evaluation of false positive and false negative rates across the paper. Along this line, none of the reported new putative cancer genes, as compared to existing methods, are independently validated to be cancer genes (based on the old+new definition, or what definition?).

This is a very reasonable point and we were glad to seek further independent validation to strengthen our assertions.

Firstly, we used available literature to support putative cancer genes discovered by RVdriver. Experimental studies that have identified our genes of interest within the same, or across different tumour types provide evidence to support our initial discoveries. This is similar to a validation approach leveraged by Dietlein et al., 2020 *Nat Genet*, where interestingly, several novel cancer genes were described without existing literature support. We assessed whether putative novel cancer genes had been linked to cancer in the cancer type of interest, had been linked to cancer in any cancer type, or had never previously been linked to cancer. The results of this analysis are pasted below and contained within Figures 4A and 4B.

Additionally, signals of positive selection uncovered by RVdriver should be maintained across cohorts within the same cancer type. As such, we interrogated the TRACERx 421 cohort (Martinez-Ruiz et al., 2023, *Nature*), for which paired multi-region DNA and RNAseq is available, to further validate our observations previously made within the lung adenocarcinoma and lung squamous cohorts of TCGA. This revealed significant concordance between the genes called as significant by the different approaches (among genes able to be tested in both cohorts, 22 of 23 had a restricted q value < 0.25 in TRACERx), highlighting that the method is able to produce consistent calls across cohorts.

See below plot, now contained within the manuscript as Figures 4E and 4F.

Finally, cancer genes, known to disrupt core cellular pathways, typically have higher ‘essentiality’ scores for cell viability compared to other genes. Databases such as gnomAD have extensively scored genes based on their tolerance to germline loss of function events, thereby inferring their necessity for cell viability. We investigated the essentiality scores, reflecting the tolerance of genes to missense and nonsense germline events and thereby their necessity for cell viability. Reassuringly, the putative novel cancer genes highlighted by RVdriver exhibited very low essentiality scores, suggesting independently and orthogonally that they are of high cellular importance, supporting a role in an aberrant tumour phenotype. Indeed, the mean score for nonsense mutations of *RVdriver* putative cancer genes was lower (0.55) than that of all other tools; whilst *RVdriver* putative cancer genes also had a low mean score for missense mutations (0.90). Please see the below related plots.

For example, no survival outcomes, which are available in TCGA, have been evaluated. Shouldn't driver mutations in cancer genes lead to some tractable biological/clinical outcome differences?

We would not expect differences in driver mutations or cancer genes to necessarily lead to tractable biological or clinical differences. Indeed, perhaps counterintuitively, recent work , such as from Smith et al (<https://www.cell.com/cell-reports/fulltext/S2211-1247(22)00313-8?_returnURL=https%3A%2F%2Flinkinghub.elsevier.com%2Fretrieve%2Fpii%2FS2211124722003138%3Fshowall%3Dtrue>), highlights that the presence or absence or particular cancer genes being mutated seldom correlates with clinical outcome in a statistically meaningful way. As such, we would not expect our putative novel cancer genes to behave differently in this regard.

3. Figure 1F is misleading as the sidebar represents 1 standard deviation, so the total length of the bar here corresponds to only ~70%CI, as compared to the usual 95%CI for measure of uncertainty. This means the variation of RNA VAF is much higher than what is shown in 1F.

We thank the Reviewer for pointing out this oversight and have replaced this with a more standard violin plot showing the full distribution of the data. Please see the updated plot below:

With that in mind, the reviewer is not convinced this conclusion: "clonal mutations, mutations in allelic imbalance and mutations showing allele-specific expression exhibited elevated RNA VAF (Figure 1F).

We understand that the manner in which we originally plotted Figure 1F might have raised questions about whether the underlying data show true differences as the Reviewer outlines. As the updated plot shows, these comparisons were all done with independent statistical tests (Wilcoxon rank tests) and so were not affected by the way the data was plotted. Each of these tests was significant to below p=0.0001. We hope that this is clear in the plot.

Furthermore with Figure 1H, even though the difference in RNA VAF between missense and synonymous mutations within CGC genes is statistically significant, there seem to a similarly wide range of RNA VAF for each category.

We completely agree that there are many mutations in CGC genes with low RNA VAF, and that the reverse is true for non-CGC genes. This is in keeping with what is known about cancer genes and non-cancer genes: both of these groups of genes contain driver and passenger mutations. What differs is the relative amounts of each. This is, in the case of Figure 1H, captured by the Wilcoxon rank test we have used to look for differences between the RNA VAF distributions. It is this difference that RVdriver leverages to highlight cancer genes using these subtle patterns. In addition, the effect size of this plot is approximately double that which would be seen using DNA VAF alone – this is strong evidence that use of RNA VAF adds value here. At the Reviewers suggestion, we have adapted the plot so that DNA VAF is included, and this added effect of RNA VAF is more obviously visible to the Reviewer and reader. Please see below and in Figure 1H and 1I.

This plot actually argues against defining only mutations that present statistically significantly higher/lower RNA VAF than the background (synonymous mutations) as driver mutations, as there are also a lot of mutations that would be missed that way. To the reviewer, this plot only suggests there are some mutations in cancer genes that will present strong signals in RNA VAF. This does not necessarily lead to the next step, to use RNA VAF to annotate functional vs. nonfunctional mutations.

We respectfully disagree. Critically, as discussed above, there is an important distinction between a cancer gene and a driver mutation. A driver mutation is context dependent. And, importantly, many cancer genes will harbour passenger mutations. **Not all mutations (whether driver or passenger) in cancer genes will behave as driver mutations.** dN/dS, for example, provides an indication of the proportion of mutations that would be expected to be drivers for each gene. This is vastly different between cancer genes, but is also very context-dependent. For example, VHL mutations are obligatory clonal events in ccRCC, but under minimal positive selection in NSCLC. Thus, assuming any mutation in VHL is necessarily a driver mutation would be erroneous.

Nonetheless, Figure 4 demonstrates the ability of RNA VAF (although N.B. not leveraging the tool RVdriver) to delineate driver from passenger mutations in cancer genes (particularly tumour suppressor genes). Furthermore, a significant proportion of CGC genes are cancer type specific, and subsequently mutations in these genes may well be passengers in other cancer types, likely reflected by a low RNA VAF.

4. Since RVdriver requires a minimum of 4 mutations within a given gene across all tumors and a q-value <0.01 as strong enough evidence, the reviewer would like to see in the TCGA data, in each cancer type, how many genes actually had enough data to run RVdriver and how many of them came out as non-cancer genes, i.e., q-value > 0.05. This data is not currently provided in the manuscript.

We thank the reviewer for this suggestion and have made this data more readily visible within Figure 2, by including the number of putative cancer genes identified by RVdriver, the number of genes definitively not identified as such, and the number of unevaluable genes within the updated plot.

5. "Across the 30 cancer types, it identified 136 instances of CGC genes." This number seems very low. How many "instances of CGC genes" are there in total as ground truth? Are we talking about a sensitivity of <10%?

As described in the above point, we have added plots to Figure 2B to make the number of CGC and non-CGC genes discovered by each tool, and the respective denominators, more obvious. We here would also underscore that it would not be reasonable to expect to discover all CGC genes within our dataset. The CGC gene list contains cancer type specific genes from a broad range of cancer types (indeed some of which are not present within the 30 analysed across the TCGA). A lack of a ground truth cancer gene list is common in these types of analysis, hence, the ability of *RVdriver* to detect comparable numbers of CGC genes to commonly used tools (figure 2A) suggests it is a sensitive approach. Moreover, of the 7 cancer gene detection tools we ran, when comparing the ‘tool-unique’ putative cancer genes, *RVdriver* identified the second-greatest fraction of CGC genes, highlighting it is also likely to be a relatively specific approach, capable of identifying novel *bona fide* cancer genes.

6. "Compared to 50 non-CGC genes (identified by RVdriver), highlight its ability to identify meaningful biological patterns". The reviewer does not follow the logic here. RVdriver has not been independently validated so what it finds may not be true.

The CGC list does not include all drivers. Indeed, no such list exists. However, there is significant literature to support leveraging CGC genes as an imperfect but useful set of ‘true positives’. For example, Dietlein et al., *Nat Genet* 2020 (<https://www.nature.com/articles/s41588-019-0572-y>) use the CGC as a gold standard in the evaluation of the performance of their tool, *mutpanning*; as do Sherman et al., *Nat Biotech* 2022 for their tool *Dig* (<https://www.nature.com/articles/s41587-022-01353-8>).

Nonetheless, we have added significant additional validation of RVdriver to the manuscript, as described above. In sum, these include an assessment of the degree of disruption to these genes in the germline (with functionally impactful genes typically tolerating less disruption), a literature search to evaluate whether other publications have suggested a role for the putative cancer genes in cancer, and an addition of a validation cohort in lung adenocarcinoma and lung squamous cell carcinoma to underscore that our method is capable of describing consistent hits across cohorts.

7. The reviewer has a lot of problems with Figure 2. 2A, it is really the insert small panel that is more meaningful as it shows the beginning of the curve, where the inclusion of non-CGC genes (as false positives) is less than 100. Across cancer types, RNA VAF does not do as well as dndscv (no.1) or mutpanning (no.2).

We apologise for the limitation of our original Figure 2. The intention of figure 2A is to show the comparable performance of RVdriver against the suite of commonly used and established methods for driver gene detection. We agree that the most relevant aspect of figure 2A is the small insert panel, and we have altered the axes of this figure to focus on the beginning of the curves. We also provide a pan-cancer figure (Figure S7).

Importantly, our approach does not seek to replace, or outperform all other existing DNA-based approaches. Rather, our approach seeks to supplement and complement them, and identify some different genes to those found using their approaches. Therefore, we take it as a sign that our approach has significant standalone utility that we were able to exhibit roughly comparable performance to other best-in-class DNA-based approaches such as *dndscv*, *mutpanning,* *oncodriveFML*, and *oncodriverclustl*.

We explored this further in a novel analysis suggested by Reviewer 2. There, we explicitly evaluated the relative impact of tools that assess mutation functional impact above and beyond that of frequentist tools, and where RVdriver is capable of adding to performance. We were reassured that RVdriver added to the ability of genomic+functional impact tools in terms of overall performance.

We also feel that an important aspect of RVdriver is its ability to identify new cancer genes that other tools miss. This is evidenced by the detection of new gene - cancer type pairs (Fig 2E and Supp Fig 10).

2B, all ROC curves look odd, as if very few data points were used. This can be proven by showing the actual number of genes (should be very small) and attach 95%CIs (should be very wide, hence really no power to compare across methods).

We have removed these cancer type specific plots which show a small number of data points as the Reviewer has identified.

2C, not clear how these ROC curves are calculated and why are they comparable since the n (not sure what this sample size is about) listed is quite different across methods.

We have removed the Area Under the ROC curve plots, with sensitivity and specificity for discovery of Cancer Gene Census genes as the basis for assessing performance, from the manuscript. We have changed our approach to evaluating performance. We now rank the gene cancer type combinations by their significance and plot the percentage of CGC genes across the top 500 genes for each method. We also compute a ‘CGC score’, utilised in the intogen pipeline to evaluate the performance of each tool. The CGC score is computed using the following equation:

$$E(R)=\Sigma_{i=1}^{N} \frac{Pi}{log(i+1)}$$

Where $Pi$is the proportion of genes with greater significance than $i$which are within the CGC list. N is a user defined threshold to consider only ‘top ranked’ genes. For our pan-cancer plot, we computed the CGC score using N=250.

2D, here non-CGC genes are switched from serving as true negatives, to serving as an unknown pool for discovery. If following such logic, then oncodrivefml and mutpanning find a lot more putative cancer genes than RNA VAF. Overall, It is odd to have the same data serving both logics. Analysis using independent truth data should be performed.

Non-CGC genes by necessity have to serve as both true negatives and an unknown pool in the absence of a ground truth, and have done so in previous studies as they contain both almost all non-cancer genes as well as undiscovered cancer genes. The point of these plots is to demonstrate that RVdriver adds additional insight into putative novel genes/cellular pathways that may be aberrantly active within a given cancer type.

Nonetheless, our additional validation analyses, described in our response to an earlier point, should hopefully provide some added confidence about specificity within these genes. In sum, these included an assessment of the degree of disruption to these genes in the germline (with functionally impactful genes typically tolerating less disruption), a literature search to evaluate whether other publications have suggested a role for the putative cancer genes in cancer, and an addition of a validation cohort in lung adenocarcinoma and lung squamous cell carcinoma to underscore that our method is capable of describing consistent hits across cohorts.

8. Figure 3 shows dndscv is doing consistently well, finding more CGC genes than RNA VAF. The reviewer counted the total number of cancer genes annotated by RNA VAF vs. dndscv and found that across 30 cancer types, dndscv found the same or more cancer genes than RNA VAF in 23 cancer types. It seems RNA VAF could further annotate more CGC genes that were missed by dndscv but by itself it does not do as well, hence not an independent tool to be used.

Our approach does not seek to replace, or outperform all other existing DNA-based approaches. Rather, our approach seeks to supplement and complement them, and identify some different genes to those found using their approaches. Therefore, we take it as a sign that our approach has significant utility that we were able to exhibit roughly comparable performance to other best-in-class DNA-based approaches such as *dndscv*, *mutpanning,* *oncodriveFML*, and *oncodriverclustl*. Moreover, we were encouraged that we discovered different genes to those tools - to us, this suggests that we might be uncovering signal of hitherto undiscovered cancer genes (of which we are certain there are many) - precisely the point of our approach. Please see the below plot.

Put another way, whilst dndscv is an excellent tool to discover cancer genes, it is not perfect (there are undiscovered cancer genes). Therefore, a hypothetical tool that identifies several new and *bona fide* cancer genes, even if it misses all of those identified by dndscv, would be very useful.

9. A similar logic loop as above was applied to studying driver vs. passenger mutations as compared to BoostDM, hence the results are not convincing.

BoostDM (Muinos et al., 2022, *Nature*) is a best-in-class *in silico* mutation saturation analytical approach; **no list of definitive true positive driver mutations exists**. It is therefore not unreasonable for us to investigate our results using BoostDM as a list of putative true positive driver mutations. However, we specifically investigate the instances where our results diverge, finding what we believe are important biological patterns, such as a novel stratification of *EGFR* driver mutations in glioblastoma and novel *KDM6A* driver mutations in urothelial cancer.

Reviewer #2:

Summary. The "RVdriver" algorithm employs RNA variant allele frequency (VAF) as a signal for positive selection to identify and classify mutations in cancer-associated genes. Black et al. first provide motivation for focusing upon instances of elevated RNA VAF. The authors proceed to validate, among canonical cancer and non-cancer genes, higher RNA VAFs in cancer versus non-cancer genes, and differences between RNA VAF from non-synonymous and synonymous mutations. A mathematical description of RVdriver is then presented. RVdriver is based upon fitting RNA VAF from nonsynonymous SNVs from a filtered MAF file with a linear mixed effects model. A mutation's function is given fixed effect size, and patient ID is given random effect size, for hierarchical dependence between RNA VAF and factors such as tumor purity and expression level. Each linear mixed model is fitted to 50 bootstrap samples of synonymous mutations, a t-value is calculated for mutation function effect size, and the median t-value is used to compute a p-value (from Student's t-distribution). After multiple test correction, a cancer-associated mutation is classified with high-confidence (q < 0.01) or low-confidence (0.01 < q < 0.05). RVdriver's performance is then evaluated (i) with AUC-ROC for cancer gene detection versus a panel of four DNA-based algorithms, and (ii) in terms of pan-cancer driver/passenger classification sensitivity/specificity versus BoostDM in silico saturation mutagenesis predictions (Muiños et al., 2020). RVdriver identified more COSMIC Cancer Gene Census (CGC) genes than non-CGC genes and produced comparable AUC versus the selected algorithms. Several gene-cancer type combinations recurred between RVdriver and DNA-based methods, and several novel combinations were also identified. Driver/passenger classification performance versus BoostDM revealed the following: (1) a strong correlation between driver/passenger annotation and RNA VAF among missense mutations in tumor suppressors; (2) notable disagreements between RNA-VAF and BoostDM (e.g., EGFR, PIK3CA); and (3) higher overall performance for driver gene identification among tumor suppressors than oncogenes.

Summary of Assessment. RVdriver offers several important contributions yet has several critical limitations. Discrepancies between RVdriver and Intogen's BoostDM for driver/passenger classification uncover closer correspondence between RNA VAF-based classification and tumor phenotype than for BoostDM-based classification. Greater VAF differences between nonsynonymous and synonymous mutations from RNA versus DNA suggest that transcriptomics provides additional information regarding mutation classification and phenotype. Black et al. also note the discovery of unique cancer-associated genes/phenotypes with RVdriver; e.g., reduced EGFR signaling in glioblastoma.

We are grateful to the Reviewer for their positive comments that RVdriver offers important contributions to the field.

RVdriver also has several impediments to publication in Genome Biology. First, higher performance has been observed from deep learning-based algorithms. It would enhance the manuscript to compare to the recent DIG method/tool (Sherman, Yaari et al., Nature Biotech, 2022), since it has also been applied to coding regions, as focused on in the current manuscript. DIG offers performance and flexibility advantages versus the analyzed method panel (e.g., dNdScv), enabling genome-wide mutation rate mapping and driver gene discovery for intergenic or noncoding elements. Use of convolutional neural networks in tandem with a Gaussian Process also allows for uncertainty quantification.

We have run DIG on our cohort using coding regions only, as the Reviewer suggests. It may have lost a degree of performance as it is our understanding that it leverages significant signal from non-coding regions (our TCGA cohort comprises paired whole exome-RNAseq data). Nonetheless it is now included in all of the relevant comparisons within Figures 2 and 3.

RVdriver may further benefit from more explicit modeling of a mutation's functional impact. Methods such as DriverPower incorporate both mutational burden and functional impact (averaged metrics from established methods), which can reweight burden-based driver gene identification (Shuai et al., Nat Comm, 2020). The RVdriver method may also benefit from combining genomic, transcriptomic, and functional impact data, as well as comparison of performance upon use of each data type alone.

We thank the Reviewer for this suggestion. It is definitely true that functional impact data acts orthogonally to the signal from RVdriver in the manner described by the Reviewer. To this end, we have provided plots displaying the relative performance of genomic, transcriptomic and functional impact-based analyses alone and in combination, and were pleased that RVdriver provided added value in each setting.

In the plots below, we compute the CGC score mentioned above for RVdriver, oncodriveFML and MutSig2CV (using only the CV pvalue), with these tools representing approaches to model the functional impact of mutations and mutational burden respectively. We also restrict to only the genes tested by RVdriver in each cancer type. The combined p values for each tool combination were then calculated using the fisher method. The combination of RVdriver with MutSig2CV and oncodriveFML improves the ordering of CGC to non CGC genes at the level of individual cancer types as well as pancancer.

We include the following section in results:

“Finally, we assessed strategies for recovering cancer genes that are ‘missed’ by *RVdriver*. Combining results from RVdriver and other tools generally resulted in an improved CGC score (Figure S11A-C). Furthermore, when genes that were identified by two or more other tools, but not by RVdriver were considered in isolation and subjected to restricted hypothesis testing, 84 of 210 genes became significant at q<0.25 (Figure S11D).”

We also spent some time attempting to incorporate functional impact information (we used CADD scores) more explicitly into the RVdriver model à la DriverPower. We found that this approach somewhat diluted the ‘transcriptomic’ signal, and would prefer RVdriver to remain orthogonal to approaches like oncodriveFML. We feel that the best approaches to cancer gene discovery cast a broad net and leverage multiple signals (e.g Intogen), and hope the Reviewer agrees that RVdriver demonstrates sufficient signal on its own to be a helpful addition to the armoury of cancer gene discovery tools currently available.

As the authors note, RVdriver may generate false positive results during cancer gene identification / driver mutation discovery. Lawrence et al., Nature (2013) note that assuming a constant background mutation rate generates numerous false positive findings due to somatic mutational heterogeneity. It would seem that RVdriver's aggregation of RNA VAF from bootstrap sampling of synonymous mutations assumes constant background RNA VAF and thus does not consider mutational heterogeneity or elevated false positive rate. Moreover, MutSigCV includes pooling of mutation counts and seq. coverage between genes that are proximal in high-dimensional covariate space. RVdriver's aggregation of sampled synonymous RNA VAFs does not incorporate such consideration.

This is a very helpful and sensible suggestion from the Reviewer and was something we had not considered prior to the initial submission of our manuscript. To that end we invested significant time in leveraging this suggestion to improve the performance of RVdriver.

With a method similar to that used in MutSig2cv, we constructed 3 ‘bagels’ of genes in similar covariate space using the published MutSig2cv gene covariates (HiC-based chromatin state estimation, cell line expression and DNA replication timing).

In the below plot, we show the distributions of RNA VAFs between bagels. There are indeed subtle differences. However, we were unable to leverage these differences to improve the performance of RVdriver despite a number of different approaches tried. For example, we attempted to restrict background synonymous mutations to only those within the bagel harbouring the gene of interest - this had the effect of worsening performance as synonymous mutations in infrequently mutated cancer types are already at a premium within our cohort. We then attempted more subtle approaches, including adding the ‘bagel’ as a random effect within the underlying model; and sampling preferentially from the ‘bagel’ harbouring the gene of interest where possible.

Unfortunately, none of these approaches improved the performance of our model, and some of them appeared to worsen it.

We think that this reflects the fact that differences in RNA VAF across ‘bagels’ are substantially more subtle than differences in mutation rate. To demonstrate this, we below provide estimates of RNA VAF and mutation rate across bagels. The differences between bagels in terms of mutation rate are more obvious - this probably underpins why they are so useful in the MutSig2cv approach.

Additionally, as can be seen below, while cluster 1 has the highest mutation rate and is associated with a high number of false positives in DNA based methods, we find that it has a lower average RNA VAF compared to clusters 2 and 3. Therefore, we anticipate that the propensity to call false positives as a result of these differences might be reduced when using RVdriver compared to tools such as MutSig2CV.

If it is of interest, we have added a bar to Figure 1G to highlight the mutation rates of the genes considered in that Figure.

In spite of this, we have made several auxiliary adjustments to RVdriver that we feel have added nuance to its approach and have definitely improved performance. Details can be found below or in the Methods.

-	Weighting for highly expressed mutations

-	This has the effect of controlling somewhat for the different expression levels between genes: for example, mutations in TTN (classical non-cancer gene harbouring a large number of passenger mutations) are typically ‘downweighted’ due to their low expression

-	Scaling for nonsense mutations

-	Here we are able to control for the expected difference in RNA VAF and scale RNA VAFs of nonsense mutations by an *a priori* assessment of how likely that mutation would be to undergo nonsense mediated decay (Supek et al., 2020)

-	No longer sampling with replacement from synonymous background

-	We have changed how we construct our background model. The most important new difference is that we no longer sample with replacement from our synonymous background – in cancer types with very small numbers of mutations we feel this has reduced our false positive rate considerably.

-	Altering the model used to determine statistical significance from a linear mixed effect model to a linear model.

-	Altering the number of synonymous mutations sampled to construct the background (from 10 to 4 per nonsynonymous mutation within the gene of interest - this number was selected to minimise false positive calls whilst not artificially inflating the p values from the statistical analysis).

Please see the relevant text from Methods:

**RVdriver**

RVdriver was run separately on individual cancer types and involved the following steps.

1. Mutation table preprocessing:
   a. Filter the MAF file for synonymous and nonsynonymous single nucleotide variants only (non-synonymous mutations comprised nonsense, missense, splice site and non-stop mutations).
   b. Detect and remove dinucleotide/trinucleotide mutations
   c. Scale the RNA VAF of nonsense mutations to account for nonsense mediated decay (see NMD scaling).
   d. Assign a weight to each mutation based on the RNA depth at the mutated position. This mitigates against potential false positives arising from mutations in lowly expressed genes. Additionally, to prevent biassing towards extremely highly expressed genes, the RNA depth of mutations are capped at the 90th percentile. Then the weight is calculated as:

$$log\_coverage = log2(capped\_RNA\_depth + 1)$$

$$mutation\_weight = log\_coverage / max(log\_coverage)$$

Finally, to ensure the RNA VAFs at positions with 0 RNA coverage still contribute to the model fitting, these mutations are assigned the same weight as a mutated position with an RNA depth of 1 non-duplicated read.

e. Calculate which genes to test within a given cancer type. A gene was tested if, within a single cancer type, it had > 3 non-synonymous mutations at an RNA depth > 7 non-duplicated reads.
f. The observed RNA VAFs within the background set of synonymous mutations differ systematically from the nonsynonymous mutations within genes analysed by *RVdriver.* To address this, we calculate a synonymous mutation scaling factor. This factor is calculated by dividing the median RNA VAF, derived from the mean RNA VAFs of missense mutations within the *RVdriver* geneset, by the median RNA VAF of all synonymous mutations at a depth of > 7 non-duplicated reads. To prevent extreme scaling, which can lead to significant deflation of the expected/observed p value distribution, this scaling factor is limited to values between 1 and 1.1.

2. Iterate through each of these genes, performing the following test.
   a. Filter for all non-synonymous mutations within the gene of interest
   b. Sample a consistent number (4) of synonymous mutations without replacement (at an RNA depth > 7 non-duplicated reads) from each tumour sample containing a nonsynonymous mutation within the gene of interest. The sampling process uses the following hierarchical stepwise approach:
      i. Consider synonymous mutations only within the tumour harbouring a mutation within the gene of interest
      ii. Consider synonymous mutations within all tumours harbouring a mutation within the gene of interest
      iii. Consider all synonymous mutations from tumours within the cancer type being considered
      iv. Consider mutations of the same type from within that sample.

This sampling approach ensures that, where possible, we control for differences in sample-sample variables such as estimated tumour content as well as differences in overall expression from the tumour compartment relative to the non-tumour compartment of the admixed bulk sample.

c. Generate a weighted linear model comparing the RNA VAF distribution of nonsynonymous mutations within the gene of interest against the background set of synonymous mutations using the following formula:

$$lm(RNA\_VAF\sim mutation\_function, weights = mutation\_weight)$$

This model includes a one-sided test to assess the directional relationship between the RNA VAF of the nonsynonymous mutations compared to the synonymous background.

d. Bootstrap the sampling and model fitting 25 times to limit the impact of sampling given RNA VAFs are variable across mutations.
e. Derive a p value by computing the geometric mean of the p values of the 25 previous tests.
f. In cases where a gene has a sufficient number of multiple mutation types, e.g nonsense and missense mutations, then these are tested separately, as well as together.

3. Perform multiple hypothesis testing correction on all gene results using the Benjamini-Hochberg approach. A gene is classified as a putative driver if the q value is < 0.25.

**NMD scaling**

Premature termination codons can result in the production of truncated proteins or the degradation of messenger RNAs by NMD. To account for NMD, we scaled the observed RNA VAFs of nonsense mutations by the degree in which it was predicted to lead to NMD. The degree of NMD, or ‘NMD score’ for a given mutation was derived from a previously published approach[^45^](https://paperpile.com/c/oYJhAl/pFg6). Then, nonsense mutations were binned according to increments of 0.1 in NMD score, and the mean RNA VAF from mutations within each bin was divided by the mean RNA VAF of missense mutations to derive a nonsense mutation scaling factor. The differences in NMD scores across the TCGA cohort and its influence on the RNA VAFs of nonsense mutations can be found within figures S6A and S6B.

Minor Comments:

*	Calculation of pan-chancer statistics for driver/passenger classification relative to the Cancer Gene Census, as well as relative to BoostDM, may be helpful for more clearly interpreting performance.

Here, the Reviewer is requesting an additional benchmark for our assessments of driver/passenger mutations. This is not possible using CGC as they only consider genes, rather than mutations. We did explore the ‘cancer mutation census’ as an alternative, but there isn’t data available for systematic driver/passenger assignment – this probably reflects that this is a very difficult task.

*	The Discussion might benefit from an additional portion that considers avenues for recovering cancer genes that were not resolved by RVdriver, e.g. PIK3CA.

We have added a short section in which we consider this more formally.

*“RVdriver* does not, then, provide an exhaustive list of cancer genes. However, our analysis suggests that through combining its results with those of other tools, a more comprehensive list would be defined. Importantly, this relationship appeared to be reciprocal, with *RVdriver* adding value (as measured with the CGC score) to other approaches. Similarly, bigger cohorts might help to uncover other bona fide cancer genes which were approaching significance in our analysis.”

*	The authors note higher performance (AUC-ROC) for missense mutations in tumor suppressor genes than in oncogenes. The implications of using RVdriver alone or with other methods could be considered in the context of such performance differences.

We agree that the differences in performance between TSGs and oncogenes are very striking. We have therefore expanded our discussion of the most readily identifiable cancer genes to include a section of how this reflects on RVdriver and its most appropriate use cases.

“The differences in the ability of RNA VAF to identify driver mutations among oncogenes and tumour suppressor genes might hold important implications for *RVdriver*. It suggests there may be asymmetry in its ability to identify putative oncogenes and putative tumour suppressor genes. Further work could characterise the relative ability of different tools to identify oncogenes and tumour suppressors; combinations leveraged strategically can help to maximise the biological insight from these approaches.”

**Second round of review**

**Reviewer 2**

Summary: This is a resubmission from Black et al. presenting the revised RVdriver algorithm to computationally nominate genes that may drive tumor growth (“cancer genes”). The authors have made substantial revisions to address reviewer comments, including 1) updating the RVdriver model; 2) modifying the metrics used to evaluate RVdriver performance and compare to DNA-based methods; 3) inclusion of orthogonal existing data to support putative genes nominated by RVdriver as drivers of cancer. The modifications have substantially improved the manuscript, and the authors present data supporting the utility of RVdriver. However, several major concerns remain, chiefly a lack of clear explanation for model design, hyperparameter choices, and parameter choices for comparing tools.

Major concerns:

1. The motivational crux of RVdriver appears to be that multiple genomic features supporting the functional nature of a mutation / gene could induce increased gene expression of the mutant allele relative to the wild type allele (increased RNA VAF). These features could include clonality, increased copy number, allele specific expression, and tumor-specific expression. This is a reasonable hypothesis. However, as Reviewer 1 previously noted, each of these underlying events has a long literature history. While the authors compare extensively to DNA-based mutation methods, the framing with respect to RNA-based and copy number-based methods remains limited. It may not be necessary to quantitatively compare methods; a more complete framing of the open problem in Introduction could suffice.

2. Fig 1H justifies the statement that nonsynonymous mutations have, on average, higher VAF than synonymous mutations within CGC genes. However, the comparison is shown only for COADREAD. What does the distribution look like in other cancer types and pan-cancer?

3. The authors state that the minimum threshold for evaluating a gene is “4 nonsynonymous mutations within a given gene at a minimum required depth of 8 non-duplicated reads, across all tumours of a specific cancer type.” How were these thresholds decided upon? Was it based on a power calculation assessing model performance? Empirical experience of the authors? Can the authors discuss how performance may change if these thresholds are altered?

4. The authors state that they now sample 4 synonymous mutations instead of 10 per nonsynonymous mutation “to false positive calls whilst not artificially inflating the p values from the statistical analysis.” Under the assumption that most nonsynonymous mutations should not be under selection relative to synonymous mutations, there should not be a need to subsample at all (a model could simply be fit on all relevant mutations simultaneously). The above statement suggests that there is an unaddressed / unexplained bias between synonymous RNA VAF and nonsynonymous RNA VAF within the genes tested. The authors discuss this somewhat in Methods, but two key questions remain open:
a. Can the authors please address the decision to subsample synonymous mutations and why sampling fewer decreases false positive rate?
b. How was the hyperparameter of 4 synonymous mutations decided upon? How does performance change as this hyperparameter is varied? Does the choice depend on cohort sample size? How should end users choose this parameter when they run RVdriver on their own data?

5. The decision to scale nonsense VAF based on propensity for NMD is reasonable. However, more details should be provided on the effect of this scaling on RVdriver performance. Can the authors provide performance metrics when scaling for NMD versus non scaling? How does performance change based on the choices of how to scale (e.g., what if NMD score were binned by quartile instead of decile)?

6. Choice of q-value for RVdriver. The authors choose q<0.25 as the threshold for RVdriver significance. This is quite a liberal threshold; q<0.1, q<0.05, or q<0.01 are more common choices. Can the authors discuss why q<0.25 was chosen as the threshold? How does the set of identified genes change as significance threshold is made more stringent and how does this effect performance metrics?

7. Choice of q-value for DNA-based methods. According to methods, different q-values thresholds were used for different tools. How were these q-values thresholds decided? Can the authors discuss how changes in q-value choices might affect reported performance metrics? (The reviewer is not suggesting the authors actually perform comparisons across different q-value sets, simply that the authors provide context for how these thresholds were chosen and how results should be interpreted when the thresholds are not consistent).

8. Comparison to existing DNA-based methods. According to Fig. 2B, RVdriver cannot evaluate most genes. DNA-based methods will typically analyze essentially all genes in the genome. As the authors themselves state in lines 299-300, relative performance could be confounded by the proportion of CGC and non-CGC genes in the gene set evaluated by each tool. However, this potential bias is not really addressed. How does the proportion of CGC to non-CGC genes compare across the gene sets used to evaluate each tool? Were analyses in Fig. 2 performed over a unified gene set across tools? If not, how does performance change if the comparison is restricted to a unified gene set?

9. Orthogonal validation of putative “cancer genes”. The reviewer appreciates the Catch-22 of “validating” potentially novel cancer genes. The authors have taken important steps to address this concern. The reproducibility within TRACERx is particularly important. However, the additional analyses raise additional questions:
a. The statement that cancer genes greater germline constraint is not supported with a literature reference or data. Can the authors provide a reference for the statement? Addition of the average constraint across CGC genes to Fig 4C and Fig. 4D would also be helpful.
b. Is it possible to compare effect sizes / odds ratios inferred from TRACERx compared to effect sizes from TCGA rather than just reproducibility by p-value?
c. Another approach is to evaluate the distance between newly identified genes and known cancer genes in a gene-gene or protein-protein interaction graph. The authors may consider pursuing such an analysis.
d. A common approach is to highlight several specific cancer-gene pairs identified by a tool and provide specific orthogonal evidence supporting the function of that gene in that cancer. Both Dietlein et al. 2020 and Sherman et al. 2022 have examples of such analyses. The authors may consider adding a similar analysis for cancer-gene pairs of particular interest.


Minor concerns:

10. We agree with Reviewer 1 that the term “cancer gene” may not be the best option. “Driver gene” is more commonly used to refer to a gene that causally contributes to the growth of an invasive tumor.

11. X-label missing on Fig. 1E.

12. Line 313. The reviewer suggests removing qualifiers such as “strikingly” from Results.

**Reviewer 3**

**Comments to author:**

The article “RNA allelic frequencies of somatic mutations encode substantial functional information
in cancers” by Black et al presents a novel method to identify cancer genes based on the RNA allele frequency of the variants observed across tumors. The method, called RVdriver, exploits differences in RNA allele frequency may reflect differences in the clonality of the mutations, but also processes affecting transcription (such as allele specific expression) or RNA stability (such as NMD). Since some of these processes may target protein-affecting mutations in cancer genes more systematically than silent mutations, the authors use a linear model to contrast the RNA VAF of protein-affecting mutations gene-wise vs a background set of silent mutations to identify potential cancer genes. Moreover, they use the RNA VAF of specific mutations in cancer genes to highlight potentially driver or passenger mutations. In comparison to several state-of-the-art methods to identify signals of positive selection across mutations in genes, RVdriver shows a good performance.

I find the article of general interest in the field of cancer genomics, and compellingly complementary to approaches designed to identify cancer genes and driver mutations. The answers of the authors to the comments raised by two previous reviewers are well reasoned. I think they have satisfactorily responded to all these points.

After reading the authors’ responses to this first round of comments, I have only a couple of remaining questions.

1. Is there any possible interference between mutational processes active across samples and the rate of synonymous/non synonymous mutations produced by them and the sampling of synonymous mutations to build the background? I wonder if sets of samples with an outsized number of synonymous mutations due to a particular combination of mutational processes active in these tumors would contribute more to the background.

2. Have the authors checked if the assignment of RNA depth of 1 non-duplicated read to mutations with 0 coverage biases the distribution of RNA VAF?

**Authors Response**

**Point-by-point responses to the reviewers’ comments:**

**Rebuttal for manuscript ‘RNA allelic frequencies encode substantial functional variation in cancers’**

We thank the Reviewers for their detailed comments. We were very pleased that our manuscript was viewed as being much improved by Reviewer 2, and as being compellingly complementary to other existing approaches by Reviewer 3, and with the general consensus that it offers an important contribution to the field.

We note various remaining criticisms which we have earnestly tried to address. In particular, Reviewer 2 focussed on the design of the *RVdriver* model, and highlighted our use of various, somewhat-arbitrary choices of hyperparameters, at certain steps of the algorithm. In scrutinising these in turn, we became convinced that further, minor modifications were necessary to circumvent the need for these thresholds.

As such, we have prepared a revised manuscript, detailing a slightly updated framework to *RVdriver*, which we hope will satisfy the outstanding comments from Reviewers 2 and 3.

The main alterations from the work we presented previously:

i) Reduced the threshold for the number of nonsynonymous mutations required within a gene of interest among a certain cancer type to be sampled, from 4 mutations to 2 (a model cannot be fit when only a single nonsynonymous mutation is present).This has allowed us to test a greater number of genes per cancer type (more than 3x), making *RVdriver* far more comparable to the DNA based approaches in the manuscript. This addresses one of Reviewer 2’s main criticisms.

ii) Redesigned the synonymous mutation sampling approach used to generate our background distribution. The number of synonymous mutations sampled per nonsynonymous mutation within a given gene (previously fixed at 4) is now scaled according to how many nonsynonymous mutations are present within that gene across the cohort. This effectively allows us to increase our confidence in our sampled background distribution as the number of mutations within a gene of interest in a cancer type increases. More details about this approach are provided in our point-by-point rebuttal below.

Other alterations were made in response to the comments from the two Reviewers. They are detailed below.

We once again thank the Reviewers for these detailed comments and hope our revised manuscript is viewed as satisfactory.

Reviewer #2: Summary: This is a resubmission from Black et al. presenting the revised RVdriver algorithm to computationally nominate genes that may drive tumor growth (“cancer genes”). The authors have made substantial revisions to address reviewer comments, including 1) updating the RVdriver model; 2) modifying the metrics used to evaluate RVdriver performance and compare to DNA-based methods; 3) inclusion of orthogonal existing data to support putative genes nominated by RVdriver as drivers of cancer. The modifications have substantially improved the manuscript, and the authors present data supporting the utility of RVdriver. However, several major concerns remain, chiefly a lack of clear explanation for model design, hyperparameter choices, and parameter choices for comparing tools.

We thank the Reviewer for their assessment of our previous revisions as having substantially improved the manuscript, and for their assessment of the utility of *RVdriver*.

Major concerns:

1. The motivational crux of RVdriver appears to be that multiple genomic features supporting the functional nature of a mutation / gene could induce increased gene expression of the mutant allele relative to the wild type allele (increased RNA VAF). These features could include clonality, increased copy number, allele specific expression, and tumor-specific expression. This is a reasonable hypothesis. However, as Reviewer 1 previously noted, each of these underlying events has a long literature history. While the authors compare extensively to DNA-based mutation methods, the framing with respect to RNA-based and copy number-based methods remains limited. It may not be necessary to quantitatively compare methods; a more complete framing of the open problem in Introduction could suffice.

We thank the Reviewer for this point. In our updated introduction, pasted below, we have acknowledged the existence of methods that attempt to classify drivers according to the above features of tumours - to the best of our knowledge, clonality and increased copy number have been explored in this context, but not allele-specific or tumour-specific expression. As such, we have amended our introduction to include the below sentence:

“Additionally, there have been other efforts to study genes according to whether their constituent mutations are more recurrently clonal, or are preserved or gained more frequently in the context of somatic copy-number alterations, than would be expected by chance[(McGranahan et al. 2015; Zapata et al. 2017; Calonaci et al. 2024)](https://paperpile.com/c/oYJhAl/SXlsp+0ydVA+a3jc).”

2. Fig 1H justifies the statement that nonsynonymous mutations have, on average, higher VAF than synonymous mutations within CGC genes. However, the comparison is shown only for COADREAD. What does the distribution look like in other cancer types and pan-cancer?

We have included all other cancer-type specific comparisons for the benefit of the Reviewer, as well as a pan-cancer comparison, in the newly added Supplementary Figures S5-S7. We find that across the majority of cancer types and pan cancer, the RNA VAFs of missense mutations are elevated relative to synonymous mutations (Figure S5 and S7). Additionally, the difference between synonymous mutations and missense mutations in CGC genes is more pronounced in the RNA VAFs compared to the DNA VAFs (Figure S7).

These figures are pasted below:

3. The authors state that the minimum threshold for evaluating a gene is “4 nonsynonymous mutations within a given gene at a minimum required depth of 8 non-duplicated reads, across all tumours of a specific cancer type.” How were these thresholds decided upon? Was it based on a power calculation assessing model performance? Empirical experience of the authors? Can the authors discuss how performance may change if these thresholds are altered?

The Reviewer rightly highlights our hyperparameter choices here. The threshold of 8 non-duplicated reads is a standard threshold in the field of measuring allelic expression (Castel et al., 2015; Castel et al., 2020; Martinez-Ruiz et al., 2023). We have made this clearer in the methods.

Nonetheless, our use of a minimum of 4 nonsynonymous mutations as a threshold above which genes were considered to be testable was indeed arbitrary. In addressing this comment, we deconstructed the RVdriver approach and the updated model now works with any gene with more than 1 nonsynonymous mutation in the cohort of interest. (With 1 nonsynonymous mutation it is not possible to obtain a p value for the comparison).

The changes, which are extensive, are outlined in full in response to the Reviewer’s next point, point 4.

4. The authors state that they now sample 4 synonymous mutations instead of 10 per nonsynonymous mutation “to false positive calls whilst not artificially inflating the p values from the statistical analysis.” Under the assumption that most nonsynonymous mutations should not be under selection relative to synonymous mutations, there should not be a need to subsample at all (a model could simply be fit on all relevant mutations simultaneously). The above statement suggests that there is an unaddressed / unexplained bias between synonymous RNA VAF and nonsynonymous RNA VAF within the genes tested. The authors discuss this somewhat in Methods, but two key questions remain open:

a. Can the authors please address the decision to subsample synonymous mutations and why sampling fewer decreases false positive rate?

b. How was the hyperparameter of 4 synonymous mutations decided upon? How does performance change as this hyperparameter is varied? Does the choice depend on cohort sample size? How should end users choose this parameter when they run RVdriver on their own data?

This is an essential observation from the Reviewer that we had not properly justified, or indeed explored, in our previous manuscript.

The rationale for this arises from the observation that ultimately many tumour samples contain tiny total numbers of synonymous mutations. Models that simply use all available data in a model that involves bootstrapping and/or resampling will be vulnerable to placing excessive weight on the small number of synonymous mutations available in a given sample. We observed that in the context of the surprisingly frequent cases where tumours harboured a small number (e.g. a total of two) synonymous mutations which both happened to have a RNA VAF of 0, repeatedly sampling (with or without replacement) from this distribution led to an excess of false positives in genes with small numbers of non-synonymous mutations across the cohorts.

We enclose a figure describing this phenomenon below:

Nevertheless, we were previously less than satisfied with having altered our hyperparameter choice to a no-less arbitrary value of 4 synonymous mutations sampled per nonsynonymous mutation within a gene of interest in a cancer type. As such, the Reviewer has provided us with an opportunity to alter this.

The following were the choices available to us:

- Construct background distribution using all available synonymous mutations within patients harbouring mutations in the gene of interest, in a cancer type
  - This risks biasing the results as the confidence in the result is determined primarily by the number of available synonymous mutations within those mutated samples (when ideally it would be determined by the number of available nonsynonymous mutations)
- Construct your background distribution from a fixed number of synonymous mutations
  - Risks false-positives if the hyperparameter is set to a high number; or failing to use all available data if the hyperparameter is set to a low number
- Construct your background distribution from a pre-determined but varying number of synonymous mutations
  - We reasoned this best balances necessary trade-offs and is most generalisable to varying cohort sizes

The threat of false-positives diminishes with increasing numbers of non-synonymous mutations.Therefore, to reduce the risk of false positives, we have implemented an approach that scales the number of synonymous mutations sampled with the number of nonsynonymous mutations within the gene of interest across the cohort. Specifically, we scale the number of synonymous mutations sampled per nonsynonymous mutation using an exponential function, where the rate of the exponential function is defined so that in a gene mutated in 5% of the cohort, 10 synonymous mutations are sampled per nonsynonymous mutation. This ensures that for genes with few mutations, which are more likely to be biased by random fluctuations, we do not erroneously assign significance. Importantly, this is also scalable to varying cohort sizes.

We have found that this approach allows *RVdriver* to be run on all of the cohorts we described in our previous manuscript, but also now the Pan Cancer TCGA cohort, without observing significant p-value inflation (Figure S14), something that was not true for our previous approach of 4 synonymous mutations sampled per patient. As such, we are confident that the change outlined above will allow users to run *RVdriver* on novel paired genomic-transcriptomic datasets of varying sizes, without having to be concerned with the hyperparameter choices the reviewer rightly criticised.

The methods section has been updated with the following description:

“To determine the number of synonymous mutations to be sampled per nonsynonymous mutation in the genes of interest, an exponential function ‘rate’ is derived. This rate is computed such that, for a gene mutated in 5% of the cohort, 10 synonymous mutations will be sampled per nonsynonymous mutation.

$$rate = log(10) / 0.05 * cohort size$$

To avoid an extreme rate in smaller cohorts, this rate is capped at 0.202 (this is the highest value that ensures 1 synonymous mutation is sampled per non-synonymous mutation when only 2 mutations are present within a gene across the cohort. The minimum of the above calculation and 0.202 is taken.

The rate is then applied to each gene being tested as described below.

Iterate through each gene being tested, performing the following test.

a. Filter for all non-synonymous mutations within the gene of interest
b. Calculate the number of synonymous mutations to be sampled per nonsynonymous mutation.

$$n = min(round(exp(rate\times nsamples), 2 nsamples)$$

Where *rate* is the cohort specific rate described above and *nsamples* is the number of samples with a nonsynonymous mutation in the gene being tested. The number of synonymous mutations sampled is capped at the minimum of the exponential curve function or double the number of mutated samples.”

Of note, we also tried several other approaches, such as fitting to all synonymous mutations simultaneously (as the reviewer suggested), and using Wilcoxon tests, as well as various iterations of linear and linear mixed effects models to test for gene significance. In general, we found that whilst the ordering of genes was maintained regardless of the approach taken, there was significant p value inflation with certain approaches. The QQ plot for a linear model, mixed effects linear model and Wilcoxon test using all synonymous mutations available from mutated samples are shown below, using the TCGA LUSC cancer type as an exemplar. The final QQ plot is for the updated *RVdriver* model we have included in our latest manuscript.

Mixed effects model fitting to all synonymous mutations in mutated samples and controlling for sample ID:

Linear model fitting to all synonymous mutations in mutated samples:

Wilcoxon fit to all synonymous mutations in mutated samples:

Final *RVdriver* approach:

5. The decision to scale nonsense VAF based on propensity for NMD is reasonable. However, more details should be provided on the effect of this scaling on RVdriver performance. Can the authors provide performance metrics when scaling for NMD versus non scaling? How does performance change based on the choices of how to scale (e.g., what if NMD score were binned by quartile instead of decile)?

We thank the reviewer for agreeing that the scaling of VAFs in nonsense mutations is a reasonable approach. We agree that justification for this change should have been made clear previously, and that we could have explored different approaches for binning mutations together to perform this scaling.

Pasted below is a comparison of the ordering of CGC and non-CGC genes in the requested iterations of the model in 6 cancer types. We find that no NMD scaling has worse ordering compared to the NMD scaled iterations, whereas there is very little difference between the quartile and decile scaled runs.

This improvement of ordering is confirmed through the calculation of the ‘CGC score’ as described in figure 2D. The pan cancer CGC score for both the scaling approaches is 0.88 (the same as figure 2D), whereas the non-scaled run gave a CGC score of 0.85.

Finally, across all cancer types, significant hits totalling 27 CGC genes and 8 non-CGC genes are lost by not performing NMD scaling, suggesting improved sensitivity, with maintained specificity, of *RVdriver* by utilising this approach.

Figure S9 has also been updated to include the RNA VAFs of nonsense mutations within NMD score quartiles.

6. Choice of q-value for RVdriver. The authors choose q<0.25 as the threshold for RVdriver significance. This is quite a liberal threshold; q<0.1, q<0.05, or q<0.01 are more common choices. Can the authors discuss why q<0.25 was chosen as the threshold? How does the set of identified genes change as significance threshold is made more stringent and how does this effect performance metrics?

q<0.25 was chosen because it was the lower bound of significance utilised by the DNA based approaches oncodriverFML and MutPanning. To compare performance across the suggested thresholds we paste below the number of CGC and non-CGC genes detected at these varying thresholds. We indeed observe that the proportion of CGC to non-CGC genes increases at more stringent q value thresholds, suggesting that the genes identified by *RVdriver* as exhibiting the strongest signal of being cancer genes are indeed *bona fide* cancer genes. However, we acknowledge that *RVdriver* should also be used to discover novel cancer genes, which are likely being identified at lower levels of significance (as evidenced by the additional 73 cancer genes discovered between q=0.01 and q=0.25; see below plot). In sum, we decided to continue to use a q-value of 0.25 for our analysis as it has been used previously in DNA based approaches and gave a reasonable trade off between sensitivity and specificity for *RVdriver.* Finally, we’d also like to emphasise that the q-value threshold is an adjustable parameter, which can be chosen by the user of RVdriver.

7. Choice of q-value for DNA-based methods. According to methods, different q-values thresholds were used for different tools. How were these q-values thresholds decided? Can the authors discuss how changes in q-value choices might affect reported performance metrics? (The reviewer is not suggesting the authors actually perform comparisons across different q-value sets, simply that the authors provide context for how these thresholds were chosen and how results should be interpreted when the thresholds are not consistent).

This could have been made more clear previously. The q values used per tool (DIGdriver: q = 0.01; dNdScv: q = 0.05; oncodriveFML: q = 0.25; oncodriveCLUSTL: q = 0.05; MutPanning: q = 0.25; MutSig2CV: q = 0.1) were chosen based on the cut-offs outlined in the respective publications for each tool. Where multiple q values were recommended/used, we chose the more liberal threshold. Importantly, whilst the q value will change the number of genes detected by each method, the ordering of statistical significance of putative hits is independent of this, and as shown in Figures 2A and 2D, *RVdriver* exhibits comparable performance to the DNA based approaches in this regard.

We agree that certain other results in the manuscript could be influenced by not having a consistent threshold, particularly Figure 2E. Here, the likely outcome of using a fixed q value of 0.05 or 0.01 would be a shift towards fewer uniquely called, non-CGC genes per tool, as the very high confidence genes detected across each method tend to be CGC genes (as shown above). Of note, however, among genes identified by one tool alone, *RVdriver* picks up a high proportion of CGC genes at the more liberal cut off of 0.25. This suggests that there is meaningful signal for cancer gene detection at the lower q value threshold of 0.25.

8. Comparison to existing DNA-based methods. According to Fig. 2B, RVdriver cannot evaluate most genes. DNA-based methods will typically analyze essentially all genes in the genome. As the authors themselves state in lines 299-300, relative performance could be confounded by the proportion of CGC and non-CGC genes in the gene set evaluated by each tool. However, this potential bias is not really addressed. How does the proportion of CGC to non-CGC genes compare across the gene sets used to evaluate each tool? Were analyses in Fig. 2 performed over a unified gene set across tools? If not, how does performance change if the comparison is restricted to a unified gene set?

The reviewer raises an important point. Whilst we added Figures 2B and S11 to highlight the number of genes being/not being tested by *RVdriver,* we did not explore how this affects performance relative to the DNA based approaches. Our alterations to the *RVdriver* methodology have removed the requirement for a minimum of 4 nonsyonymous mutations within the gene of interest in a cancer type. This change has significantly increased the number of gene:cancer type combinations tested across the TCGA cohort from 18,596 genes to 59,365.

Further, we have added a new Figure S12 (pasted below). This essentially recapitulates Figure 2A, but on a unified geneset studied across all tools. This shows that across the cancer types with > 50 eligible genes, *RVdriver* has comparable performance to the DNA based approaches using this unified geneset.

It is important to note that we are not the only tool to not test all genes in the genome: both *oncodriveFML* and *oncodriveCLUSTL* have mutation count requirements and *oncodriveCLUSTL* tests far fewer gene, cancer type combinations across TCGA (23,574) than *RVdriver* (59,365).

9. Orthogonal validation of putative “cancer genes”. The reviewer appreciates the Catch-22 of “validating” potentially novel cancer genes. The authors have taken important steps to address this concern. The reproducibility within TRACERx is particularly important. However, the additional analyses raise additional questions:

a. The statement that cancer genes greater germline constraint is not supported with a literature reference or data. Can the authors provide a reference for the statement? Addition of the average constraint across CGC genes to Fig 4C and Fig. 4D would also be helpful.

We thank the reviewer for pointing out the lack of a reference for this statement. Further, we have added a new Figure S16A to address this concern, which is pasted below.

We find that CGC genes have a significant reduction in the upper bound fraction of observed/expected counts for both missense and nonsense germline mutations, with this relationship being more pronounced in nonsense mutations. This Figure provides additional context and we hope that it gives the Reviewer further confidence that the putative cancer genes called by *RVdriver* are indeed high quality, with lower observed / expected counts of germline mutations relative to DNA based tools. Figures S16B and S16C provide additional information, plotting the overall distributions of scores for putative cancer genes from each tool (also pasted below). Finally, as suggested by the reviewer, the average score for CGC genes has been added to Figures 4C and 4D and Figures S16B and S16C.

b. Is it possible to compare effect sizes / odds ratios inferred from TRACERx compared to effect sizes from TCGA rather than just reproducibility by p-value?

We thank the reviewer for this suggestion, we agree that the Figure they describe would indeed be convincing. Figure 4F has been updated to reflect this (pasted below). We were encouraged to find that for both LUAD and LUSC samples, there was a significant correlation between the *RVdriver* gene effect sizes across TCGA and TRACERx, suggesting that the tool is able to produce consistent calls across cohorts.

c. Another approach is to evaluate the distance between newly identified genes and known cancer genes in a gene-gene or protein-protein interaction graph. The authors may consider pursuing such an analysis.

We sincerely appreciate this suggestion from the Reviewer. We truly hope that we have made sufficient alterations to our approach to have responded sufficiently to the Reviewer’s substantive criticisms of our previous manuscript. We further hope that we have provided sufficient evidence of the veracity of our putative novel cancer genes, in the form of: i) *RVdriver* identifying the greatest fraction of CGC genes among gene only identified by a single tool among all tools tested (see Figure 2E); ii) the substantial literature support for *RVdriver* putative hits (see Figure 4A and 4B); iii) the germline constraint of *RVdriver* putative cancer genes being more similar to *bona fide* cancer genes than the hits of other DNA-based approaches (see Figure 4C and 4D); the substantial similarity between TCGA NSCLC and TRACERx NSCLC *RVdriver* putative cancer gene hits (see Figure 4E and 4F).

We further have found that gene-gene and protein-protein interaction analyses in this setting can sometimes be low-yield, in the sense that they identify common pathways that occur recurrently across cancer types, and should be associated as much with known cancer genes as with putative novel cancer genes. With this in mind, we sincerely hope that the Reviewer agrees that such analyses might be considered beyond the scope of this manuscript.

d. A common approach is to highlight several specific cancer-gene pairs identified by a tool and provide specific orthogonal evidence supporting the function of that gene in that cancer. Both Dietlein et al. 2020 and Sherman et al. 2022 have examples of such analyses. The authors may consider adding a similar analysis for cancer-gene pairs of particular interest.

We thank the Reviewer for this suggestion. We have added a paragraph to this effect to the results section, in which we have overviewed some of the novel gene-cancer type combinations and outlined orthogonal evidence supporting their role in cancer.

“Importantly, *RVdriver* also implicated several novel cancer genes that would be worthy of further investigation and analysis to determine their role in cancer development and progression. These include but are not limited to:

*SMARCA1,* detected in kidney papillary cell carcinoma (KIRP) and liver hepatocellular carcinoma (LIHC). *SMARCA1* is a member of the SWI/SNF chromatin remodelling complex, which regulates the transcription of genes by altering chromatin structure . Other members of the SWI/SNF complex, namely *ARID1A/B* and *ARID2* are well-established tumour suppressor genes [^24^](https://paperpile.com/c/oYJhAl/Dxmf5)

In both KIRP and LIHC, epigenetic alterations are frequently observed. In KIRP, mutations in chromatin remodelling genes occur in ~ 53% of tumours [^38^](https://paperpile.com/c/oYJhAl/9raV). Similarly, in LIHC, ARID1A (7%), ARID2 (5%) and BAP1 (5%) mutations are frequent and contribute to disrupting the epigenetic landscape of hepatocellular carcinomas [^39^](https://paperpile.com/c/oYJhAl/azHW). The presence of *SMARCA1* mutations in both cancer types suggests a broader disruption of chromatin remodelling that drives tumour development through altered gene expression in tumour cells.

*HUWE1,* detected in head and neck squamous cell carcinoma (HNSC) and bladder adenocarcinoma (BLCA). *HUWE1* (HECT, UBA and WWE domain containing E3 ubiquitin protein ligase 1) encodes an E3 ubiquitin ligase that regulates the degradation of multiple proteins including *MYC, TP53* and components of the *WNT* signalling pathway. As such, it has been implicated in regulation of genomic stability and cellular proliferation [^40^](https://paperpile.com/c/oYJhAl/iN7I)^,^[^41^](https://paperpile.com/c/oYJhAl/VwgR). Given the core cancer related pathways governed by these genes, mutations in *HUWE1* could well be functionally relevant in cancer, and has previously been suggested to be a potential tumour suppressor gene specifically in HNSC [^24^](https://paperpile.com/c/oYJhAl/Dxmf5).

*IRF2,* detected in LIHC. *IRF2* (Interferon Regulatory Factor 2) disruption has been associated with immune evasion phenotypes through a lack of MHC-I antigen presentation [^42^](https://paperpile.com/c/oYJhAl/AoAJ) and was detected as a pan cancer tumour suppressor gene in Bailey et al., 2018 [^24^](https://paperpile.com/c/oYJhAl/Dxmf5). LIHC tumours frequently arise in the context of chronic inflammation driven by factors such as viral infections (e.g. hepatitis B and C) and alcohol consumption [^43^](https://paperpile.com/c/oYJhAl/Jy8K). Mutations in genes regulating the immune response such as *IRF2*, may weaken immune surveillance in this inflammatory environment, facilitating tumour formation and progression.

*PLK4,* detected in lung squamous cell carcinoma (LUSC). *PLK4* (Polo-Like Kinase 4) is a member of a broader family of genes which are critical regulators of the cell cycle, with *PLK4* required for centriole replication [^43,44^](https://paperpile.com/c/oYJhAl/Jy8K+rshv). Overexpression of PLK4 has been shown to induce supernumerary centrosomes, driving genomic instability and an invasive phenotype [^45^](https://paperpile.com/c/oYJhAl/LSbi). LUSC tumours are characterised by extreme genomic instability, with genome doubling events occurring in 83% of LUSC tumours within the TRACERx 421 cohort [^46^](https://paperpile.com/c/oYJhAl/TwXH). Activating mutations of *PLK4* may contribute to this instability, facilitating tumorigenesis and progression by enabling chromosomal missegregation and aneuploidy.“

Minor concerns:

10. We agree with Reviewer 1 that the term “cancer gene” may not be the best option. “Driver gene” is more commonly used to refer to a gene that causally contributes to the growth of an invasive tumor.

If the Reviewer would permit us, we would like to politely push back on this statement - we prefer to describe mutations as ‘driver’ or ‘passenger’, whilst describing genes as ‘cancer’ or ‘non-cancer’. We are of course happy to follow editorial advice on this though.

11. X-label missing on Fig. 1E.

Corrected.

12. Line 313. The reviewer suggests removing qualifiers such as “strikingly” from Results.

We have removed this from the text.

Reviewer #3: The article “RNA allelic frequencies of somatic mutations encode substantial functional information

in cancers” by Black et al presents a novel method to identify cancer genes based on the RNA allele frequency of the variants observed across tumors. The method, called RVdriver, exploits differences in RNA allele frequency may reflect differences in the clonality of the mutations, but also processes affecting transcription (such as allele specific expression) or RNA stability (such as NMD). Since some of these processes may target protein-affecting mutations in cancer genes more systematically than silent mutations, the authors use a linear model to contrast the RNA VAF of protein-affecting mutations gene-wise vs a background set of silent mutations to identify potential cancer genes. Moreover, they use the RNA VAF of specific mutations in cancer genes to highlight potentially driver or passenger mutations. In comparison to several state-of-the-art methods to identify signals of positive selection across mutations in genes, RVdriver shows a good performance.

I find the article of general interest in the field of cancer genomics, and compellingly complementary to approaches designed to identify cancer genes and driver mutations. The answers of the authors to the comments raised by two previous reviewers are well reasoned. I think they have satisfactorily responded to all these points.

We are extremely grateful for this generous characterisation of the manuscript.

After reading the authors’ responses to this first round of comments, I have only a couple of remaining questions.

1. Is there any possible interference between mutational processes active across samples and the rate of synonymous/non synonymous mutations produced by them and the sampling of synonymous mutations to build the background? I wonder if sets of samples with an outsized number of synonymous mutations due to a particular combination of mutational processes active in these tumors would contribute more to the background.

The reviewer raises an important point - it could well be that samples in which certain mutational processes are active, which in turn influence the mutational burden, could indeed contribute more synonymous mutations to the background distribution. This would be a particularly significant problem if both

i) the number of synonymous mutations sampled per patient to form the background was sufficiently high to require sampling from the additional pools of synonymous mutations

AND

ii) the RNA VAFs of synonymous mutations differed significantly across samples with varying numbers of synonymous mutations.

With regards to point i), Supplementary Figure S8 shows the proportion of tests in which the additional pools of sampling approaches are used per cancer type:

We find that in the majority of cancer types, most tests only utilise a matched number of synonymous mutations per sample. However, in certain cancer types (primarily those with smaller cohort sizes and lower TMBs, such as UVM, KICH, LAML and PCPG) the likelihood of sampling from other mutated samples as well as the non-mutated samples increases.

Were the RNA VAFs of synonymous mutations in these cancer types to differ as the number of synonymous mutations per sample changed, we might need to alter our methodology to reflect the Reviewer’s concerns. Reassuringly, however, we find fairly modest differences in the average RNA VAFs of synonymous mutations in the cohorts where sampling goes beyond the mutated tumours (See plot below).

Therefore:
1. Very few tests require additional sampling from other non-mutated samples, especially in more highly mutated cancer types.

2. Even when sampling does occur in the additional pools, there does not appear to be a significant bias toward lower/higher VAFs, suggesting that *RVdriver* will perform similarly regardless of the addition of these mutations to the background.

To confirm this further, we performed a ‘strict’ sampling iteration of the method where synonymous mutations could only be sampled from the mutated samples. If a sample did not have a sufficient number of synonymous mutations then all synonymous mutations were taken - but additional sampling from other mutated samples and beyond was not performed. We found that the overall ordering of CGC and non-CGC genes was consistent regardless of the sampling approach (CGC score of 0.88 for RVdriver and 0.87 with strict sampling).

Of note, though, the sensitivity of *RVdriver* is slightly reduced when the additional sampling approaches are not utilised:

Therefore, to accommodate this suggestion and allow flexibility to our approach and to users of *RVdriver*, we have added the strict sampling as an optional parameter that can be set.

2. Have the authors checked if the assignment of RNA depth of 1 non-duplicated read to mutations with 0 coverage biases the distribution of RNA VAF?

The choice to include mutations with a coverage of 0 reads ultimately reflects the signal we believe is contained within these mutations. As an example, we have pasted below Figure 1G, on the left, displaying the RNA VAFs of 5 classical non-cancer genes among the COADREAD cancer type. Here, very few of all mutations have coverage of >1 read (max 6% in *RYR2*) - there is clear value in incorporating zero-coverage mutations in triaging these genes as a passenger. This is the reason we have incorporated this feature in the model. To further emphasise this point, we also provide an alternate Figure 1G, on the right, where mutations with a depth of 0 reads have been removed. A greater proportion of mutations are now considered to be expressed (max 16% in *RYR2* and 13% in *OBSCN*), and there is substantially less information available to assign the probability of these genes as passengers, especially in the case of *CSMD3* where only one mutation remains.

**Third round of review**

**Reviewer 2**

The authors have done an admirable job addressing our concerns, particularly reflected in the changes they have made to the model's implementation. I recommend the article for publication without further concerns.
